# *C. elegans* Cytoplasmic Isocitrate Dehydrogenase Neomorphic G98N and R133H Mutants Produce the Oncometabolite 2-Hydroxyglutarate

**DOI:** 10.3390/ijms26178238

**Published:** 2025-08-25

**Authors:** Melissa Bouchard, Anne McAllister, Noah S. Bourlett, Chelsea Hoyt, Laurent Calcul, Katherine M. Walstrom

**Affiliations:** 1 Division of Natural Sciences, New College of Florida, Sarasota, FL 34243, USA; 2 Department of Chemistry, University of South Florida, Tampa, FL 33620, USA; calcul@usf.edu

**Keywords:** dehydrogenase, enzyme kinetics, isocitrate, 2-hydroxyglutarate, *C. elegans*

## Abstract

Isocitrate dehydrogenase (IDH) catalyzes the conversion of NAD(P)^+^ and isocitrate to NAD(P)H and α-ketoglutarate (αKG). The cytoplasmic enzyme IDH1 is important for producing NADPH for biosynthesis and for protecting against oxidative stress. IDH1 mutants, such as R132H found in glioblastomas and other types of human cancers, have a neomorphic activity that uses NADPH to reduce αKG to 2-hydroxyglutarate (2HG). 2HG interferes with the activity of important enzymes such as histone demethylases and TET demethylases. We hypothesized that *Caenorhabditis elegans* could be a good model system for studying oncogenic properties of mutant IDH1. To test this, we purified *C. elegans* cytoplasmic IDH-1 and two mutants, G98N and R133H, which correspond to human IDH1 mutants G97N and R132H, respectively. We found that the wild-type IDH-1 had similar kinetic properties to human IDH1, and it could produce small amounts of 2HG. We also found that the R133H mutant had a lower K_M_ for αKG than human R132H in steady-state enzyme kinetic experiments, and it produced almost exclusively 2HG in the presence of NADPH and αKG. The G98N mutant had a higher k_cat_ in the forward direction than the comparable human G97N mutant, and the G98N mutant produced a smaller amount of 2HG compared to the R133H mutant. These results suggest that *C. elegans* strains with IDH-1 mutations could be a good model system for studying the effects of 2HG in eukaryotic organisms.

## 1. Introduction

**Figure 1:**
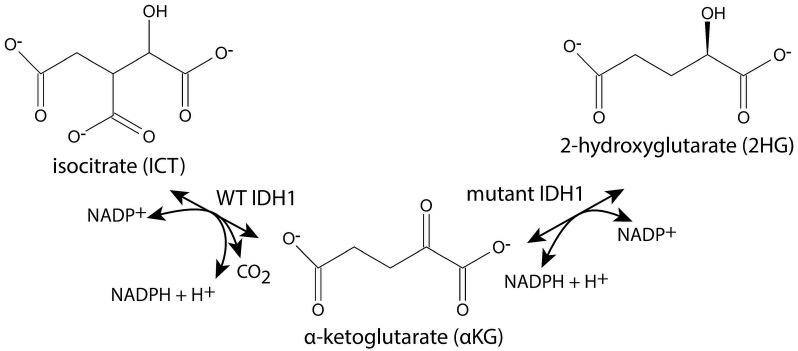
IDH1 reactions. Wild-type (WT) IDH1 catalyzes the reaction shown on the left, and some mutants of IDH1 catalyze both of the reactions shown.

Cytoplasmic isocitrate dehydrogenase (IDH1, EC 1.1.1.42) uses NADP^+^ for the reversible oxidative decarboxylation of isocitrate (ICT) to α-ketoglutarate (αKG) and the production of NADPH (Figure 1). While the pentose phosphate pathway is the main source of cellular NADPH [1,2], NADPH from IDH1 is important for the biosynthesis of lipids in specific cell types [1,3]. In addition, NADPH protects against oxidative stress by acting as a cofactor in the regeneration of the thiol form of glutathione, which protects cells from reactive oxygen species. αKG can be converted to glutamate and subsequently to glutamine, an important source of nitrogen during de novo nucleic acid biosynthesis [4,5]. Under stressful conditions such as hypoxia, cells depend on IDH1 to convert αKG to ICT, which is further metabolized to acetyl-CoA for lipid synthesis [6].

Human IDH1 mutants were originally identified in colorectal cancer and then at a high frequency in gliomas [7,8,9,10,11]. Since then, numerous mutants in IDH1 and in the structurally and functionally similar mitochondrial enzyme IDH2 have been found in various cancers [12,13,14,15]. As shown in Figure 1, the mutated enzymes have a neomorphic activity that converts αKG and NADPH to D-2HG ((*R*)-2HG) and NADP^+^ [16,17]. The consumption of NADPH reduces its availability for the biosynthetic and anti-oxidative processes described above, making the cells more susceptible to oxidative stress [1,18,19]. The production of 2HG inhibits enzymes that require αKG to function, such as Jumonji C domain–containing histone lysine demethylases and the ten-eleven translocation (TET) oxygenases that are required for DNA demethylation (reviewed in [20,21,22,23]). Mutant IDH1 also disrupts metabolic pathways because its production of αKG is low, and in the reverse direction, it produces 2HG from αKG, which reduces ICT production and requires glutaminolysis to replenish αKG [24,25,26,27]. Eukaryotic cells contain 2HG dehydrogenase, but the enzyme activity is inadequate to prevent the accumulation of 2HG in cells with mutant IDH1 [28].

The most prevalent IDH1 mutation found in human cancers is R132H, and this residue is strictly conserved [29]. Arg 132 is one of the arginine residues that bind to isocitrate, so its conversion to His lowers the K_M_ for isocitrate [30,31]. The production of 2HG by R132H mutants seems to be well conserved because comparable mutants of *E. coli* and yeast IDH produce 2HG [32,33]. A IDH1 G97D mutant was found in astrocytomas and colorectal cancer cell lines [9,34]. Rendina et al. tested the G97D mutant and found that a G97N mutant produced more 2HG [30]. The reverse of the normal IDH1 reaction requires CO_2_, and the G97N mutant was hypothesized to restrict a tunnel in IDH1 that allows CO_2_ to diffuse back into the active site. However, other experiments suggested that a lack of CO_2_ could not completely explain the neomorphic production of 2HG by G97N [30].

We produced comparable mutations (G98N and R133H) in *C. elegans* IDH-1 to determine the kinetic properties of the enzymes and test whether they produced 2HG, and we found that they do. Since mutant *C. elegans* strains can easily be produced via CRISPR and other transgenic methods [35,36], and since there is a high level of homology between worm and human proteins [37], we propose that *C. elegans* could be a good model system to further study IDH-1 mutants and their effects on cells and body systems. In fact, a recent study in *C. elegans* heterozygous for an R133H mutation showed that the worms contained 2HG [38], and our results demonstrate that homodimers of both G98N and R133H mutants produce 2HG.

## 2. Results

### 2.1. IDH-1 Structure Prediction

**Figure 2:**
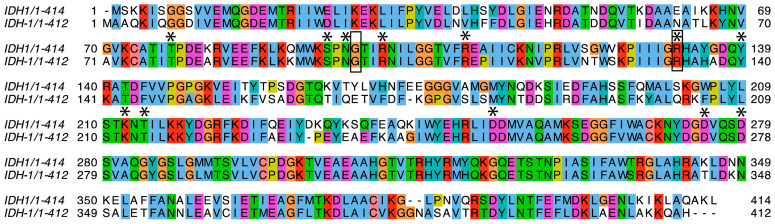
Protein sequence alignment. A sequence alignment between human IDH1 (top line) and *C. elegans* IDH-1 (bottom line) is shown. The amino acids mentioned in the text that are important for binding and/or catalysis are marked with an asterisk above. The amino acids that were mutated in IDH-1 for this project are surrounded by a box. The colored boxes are based on the ClustalW color scheme and indicate conserved amino acids as well as the G and P residues.

**Figure 3:**
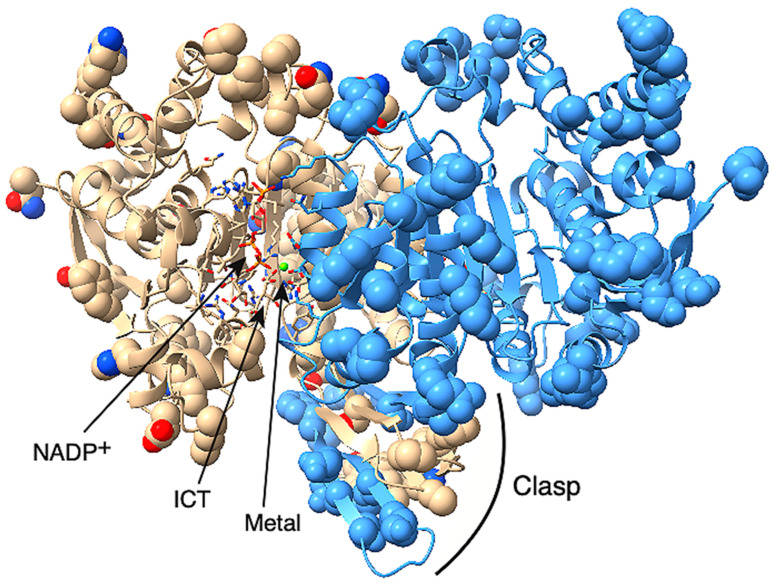
Homology model of *C. elegans* IDH-1. The IDH-1 dimer is shown with the opening of the left-hand active site facing forward, and the opening of the right-hand active site facing backward. The active site on the left contains NADP^+^, ICT, and a metal ion (green sphere). The substrates and the amino acid side-chains involved in substrate binding and catalysis are shown in stick mode and are colored based on the element. The amino acids that are different between human IDH1 and *C. elegans* IDH-1 are shown in space-filling mode. The rest of the protein is shown as a cartoon. One dimer is shown in beige, and the visible side-chains are colored by element. The other subunit is colored blue except that the amino acids from this subunit that are in the left-hand active site are colored by element. The clasp region is also labeled.

We studied *C. elegans* cytoplasmic isocitrate dehydrogenase (IDH-1, NP_001255393.1) to determine if it could be a good model for studying the effects of dominant mutants of human IDH1 (NP_001269316.1) in cancer development. We did a sequence alignment and found that IDH-1 is a member of IDH Superfamily II [29], which includes dimeric eukaryotic and some prokaryotic enzymes (Supplementary Figure S1: IDH-1 sequence analysis). The protein sequences of the human and worm proteins are 75% identical and 85% similar (Figure 2). To find the locations of the amino acid differences between the human and worm proteins, we made a homology model of *C. elegans* IDH-1 and used space-filling view to visualize the changed amino acids (Figure 3).

IDH-1 is a dimer based on its homology to proteins that have already been crystalized (such as [39]) and based on its predicted structure determined by the AlphaFold server (see Supplementary data Structure S1: AlphaFold model of IDH-1). Currently, AlphaFold can only incorporate a few specific ligands into a protein model, and these ligands do not include ICT. A large conformational change occurs in the presence of all the ligands (discussed below), so AlphaFold could only be used to model IDH-1 in the open form without ICT bound. There are published crystal structures of human IDH1 with all the substrates bound, so we used a homology model for IDH-1 to show where the substrates would bind (Figure 3, Supplementary data Structure S2: Homology model of IDH-1). To check if the subunit interactions in the IDH-1 model were similar to those in the human IDH1 dimer, we compared the intersubunit contacts and hydrogen bonds in both proteins and found that they were very similar (Supplementary Figure S2: IDH-1 structure analysis).

Each IDH1 dimer has two active sites (one on each side of Figure 3), and each active site involves residues from both subunits. The amino acid residues discussed in this paragraph are given the number of the amino acid in human IDH1. The amino acid numbers in the rest of the article and in the figure legends and methods are based on the protein (either *C. elegans* IDH-1 or human IDH1) being discussed. The metal (either Mg^2+^ or Mn^2+^, see below) is bound to Asp 252, Asp 275, and Asp 279, [40,41], and ICT binding depends on Arg 100, Arg 109, and Arg 132 [42,43]. In addition, Thr 77, Ser 94, Asn 96, Thr 214, Tyr 139, and Lys 212 are required for ICT binding and/or enzyme activity [39,42,44,45,46]. Figure 2 shows that these active site residues are conserved in IDH-1, and Figure 3 shows that most of the changed amino acids between human IDH1 and *C. elegans* IDH-1 are around the periphery of the dimer. Our analysis of the hydrogen bonds between the IDH-1 model and human IDH1 showed that the same amino acids are involved in ICT binding (Supplementary Figure S2).

### 2.2. Enzyme Kinetics

**Figure 4:**
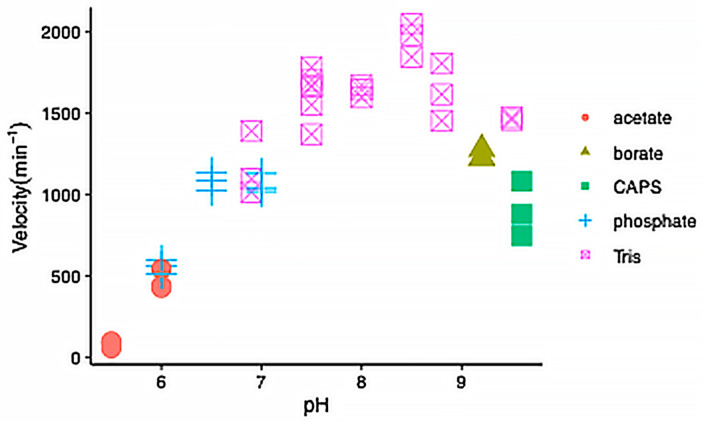
pH dependence of wild-type IDH-1 activity. The velocity versus pH is shown with each symbol representing an individual reaction. The reactions were performed at 25 °C and contained 40 mM of the buffer indicated in the legend, 1 mM MnCl_2_, 2 mM ICT, and 500 μM NADP^+^, and 15 nM IDH-1.

We expressed wild-type IDH-1 and the mutants G98N and R133H, corresponding to the G97N and R132H mutants in human IDH1, respectively. We first characterized the wild-type enzyme. In preliminary experiments, we confirmed that it had 35-fold higher activity in the presence of NADP^+^ compared to NAD^+^, and we measured a 3-fold higher activity in the presence of Mn^2+^ compared to Mg^2+^. We also tested its pH profile and found that it had the highest activity between pH 7.5–8.8 (Figure 4).

**Figure 5:**
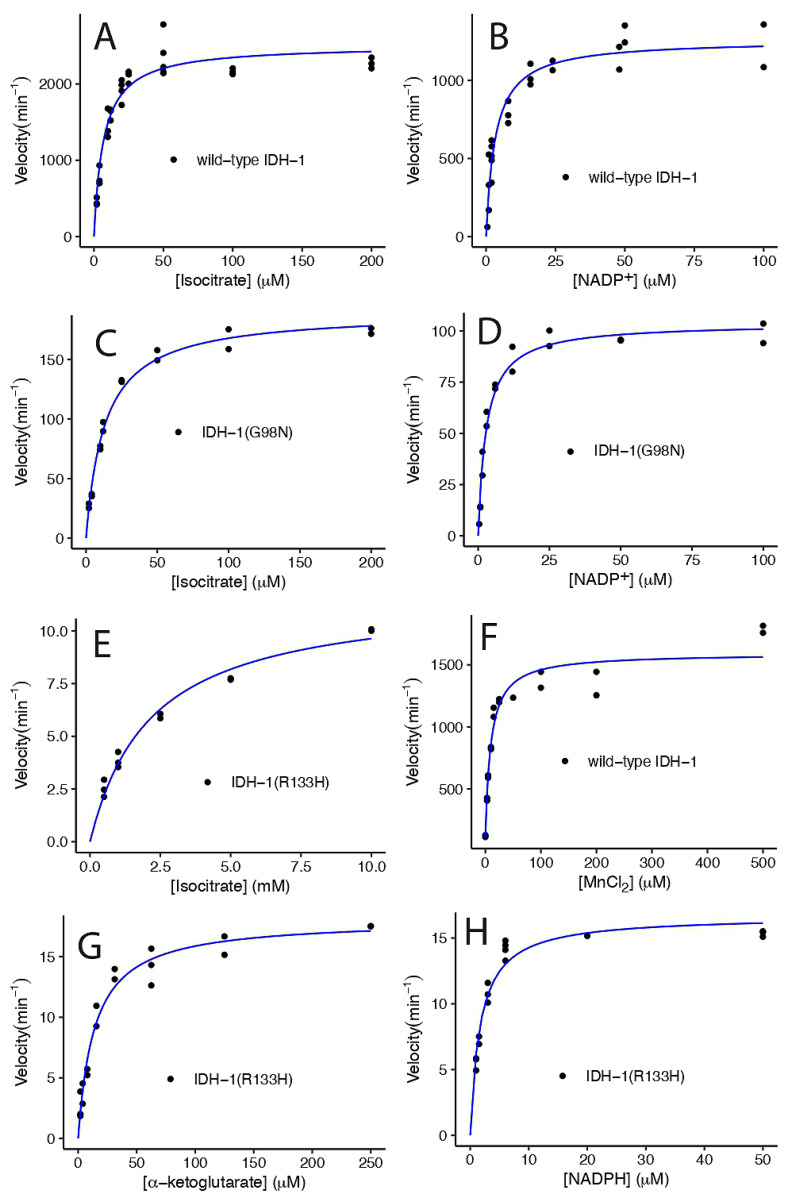
Michaelis-Menten graphs of wild-type and mutant IDH-1 at 25 °C. The wild-type IDH-1 results are shown in (**A**,**B**,**F**). The G98N results are shown in (**C**,**D**), and the R133H results are shown in (**E**,**G**,**H**). Each panel shows the velocity versus the concentration of the substrate shown on the *x*-axis. All the velocities were divided by the total enzyme concentration added to the sample, so the maximum velocities indicate the k_cat_ value. The points represent individual measurements, and the blue lines show the best fit to the Michaelis-Menten equation v = (k_cat_ × [S])/([S] + K_M_). For panel F, the data vs. MnCl_2_ were fit to the equation v = (k_cat_ × ([S] + C_0_))/(([S] + C_0_) + K_M_), where C_0_ is the amount of Mn^2+^ present when no MnCl_2_ was added to the reaction. The results of the fits are shown in Table 1, and the statistical analyses of the fits are shown in Supplementary Table S1: Kinetics statistics. The reaction conditions for each set of data are described in the methods.

While optimizing the conditions for the reactions with R133H, we measured 40% higher activity at 2 mM MnCl_2_ (k_cat_ = 18 min^−1^) compared to 1 mM (13 min^−1^) at 25 °C for the forward reaction and obtained similar results for the reverse reaction (1 mM, 13 min^−1^; 2 mM, 17 min^−1^). Therefore, the kinetics experiments with R133H shown in Figure 5 were done with 2 mM MnCl_2_.

Using the optimized enzyme reaction conditions, we determined the K_M_ and k_cat_ for each enzyme for a variety of substrates (Figure 5, Table 1). Wild-type IDH-1 had a similar K_M_ for ICT and NADP^+^ and a higher k_cat_/K_M_ compared to human IDH1 (Table 1 and Table 2). The K_M_ for Mn^2+^ that we measured for IDH-1 was 4.5-fold higher than that reported for human IDH1. Some researchers have used EDTA to remove the metal from purified IDH1 before measuring metal binding [30,31], but we did not do this, so this may be one reason for the difference. When we performed steady-state kinetics by varying MnCl_2_, we found that the enzyme had a small amount of activity (7.5% of k_cat_) when no divalent ion was added, suggesting that it bound to a metal in the *E. coli* where it was expressed (Figure 5F). We accounted for the additional metal ions in our analysis by adding a constant term to [MnCl_2_] in the Michaelis-Menten equation, and we obtained 0.6 ± 0.6 μM, which is a small but imprecise correction compared to the K_M_ of 9 ± 2 μM.

We found that the G98N and R133H mutants had a reduced k_cat_, 8% and 0.5% of the wild-type IDH-1 k_cat_, respectively, when ICT was varied. The G98N IDH-1 mutant had a 16-fold higher k_cat_ compared to the R133H mutant. In contrast, the human G97N mutant had a comparable k_cat_ to the human R132H mutant [30]. We also found that the G98N mutant had similar K_M_ values for ICT and NADP^+^ as wild-type IDH-1, while human G97N had lower K_M_ values for both substrates compared to human wild-type IDH1 (Table 1 and Table 2). The K_M_ for ICT for the R133H mutant was much higher than for the wild-type enzyme (Table 1). This was expected because crystal structures of human IDH1 showed that R132 bound to ICT in the active site [30,39], and the R132H mutant had a high K_M_ for ICT (Table 2). The *C. elegans* wild-type IDH-1 homology model also has H-bonds between R133 and ICT (see Supplementary Structure S2 and Figure S2). We found that the R133H mutant had a lower K_M_ for NADPH compared to the wild-type IDH-1 K_M_ for NADP^+^ (Table 1), and this was expected based on results with human IDH1 suggesting that NADPH remains bound to the R132H enzyme, and that this is one reason that it produces more 2HG [30].

### 2.3. Analysis of G98N and R133H

The G98N and R133H mutants had two main differences when compared with the corresponding human enzymes. The G98N enzyme had a higher k_cat_, and the R133H mutant had a lower K_M_ for αKG in the reverse reaction (Table 1 and Table 2). Comparisons of the homology model of IDH-1 to the crystal structures of human IDH1 indicated that there were no differences in amino acid residues in and around the active site (Figure 2, Figure 3 and Figure S2). Therefore, we investigated some theories about why there were these differences.

We looked at the amino acids involved in contacts between the two subunits, and homology models of both mutants indicated that they were very similar to the wild-type enzyme (Figure S2). Since R133 points into the active site, it was not involved in intersubunit interactions. Interestingly, in the G98N mutant, the N98 residue in one subunit makes a very close contact with I215 in the other subunit (Figure S2), and I215 is conserved in human IDH1 (Figure 2). Since Gly is so much smaller than Ile, G98 is not involved in intersubunit interactions in the wild-type enzyme. This suggests that there could be some steric hindrance in the G98N mutant. The stress from this interaction could propagate through the structure so that amino acid changes far from the active site could affect enzyme activity.

**Figure 6:**
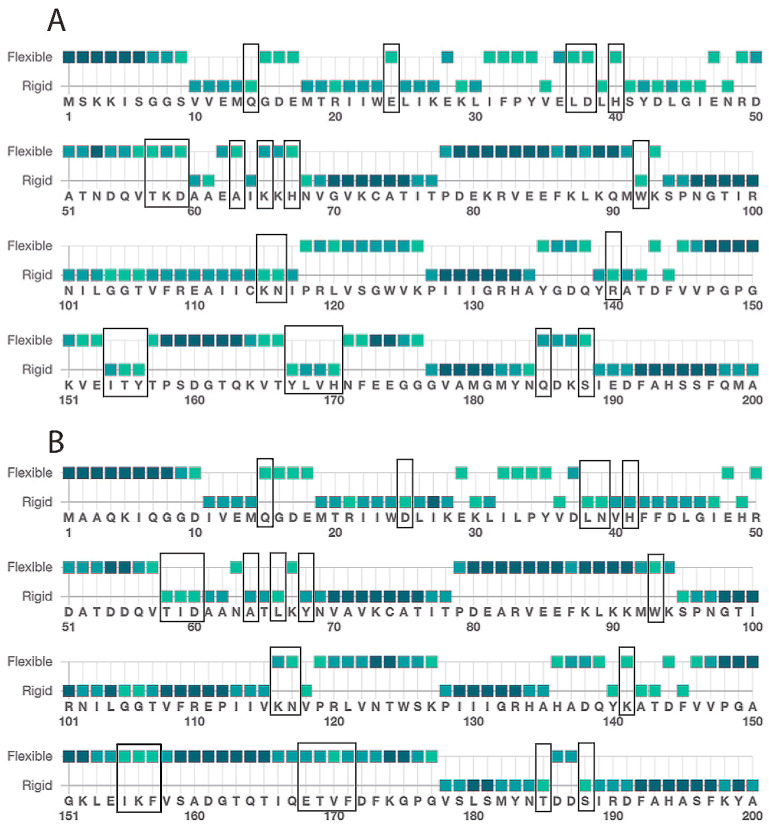
Medusa flexibility analysis. The amino acid sequences of (**A**) human IDH1 and (**B**) *C. elegans* IDH-1 were submitted to the Medusa server (https://www.dsimb.inserm.fr/MEDUSA/, accessed on 25 September 2024), and the predicted flexibility is shown using a two-point scale. This figure shows predictions for amino acids 1–200, and the full analyses are shown in the Supplementary Figure S3: MEDUSA analysis of IDH1 and IDH-1. The amino acids with changed flexibility are indicated with a black box in (**A**,**B**). The color of the square represents the confidence of the prediction, with light green < 0.5, medium green 0.5–0.6, and dark green > 0.6.

**Figure 7:**
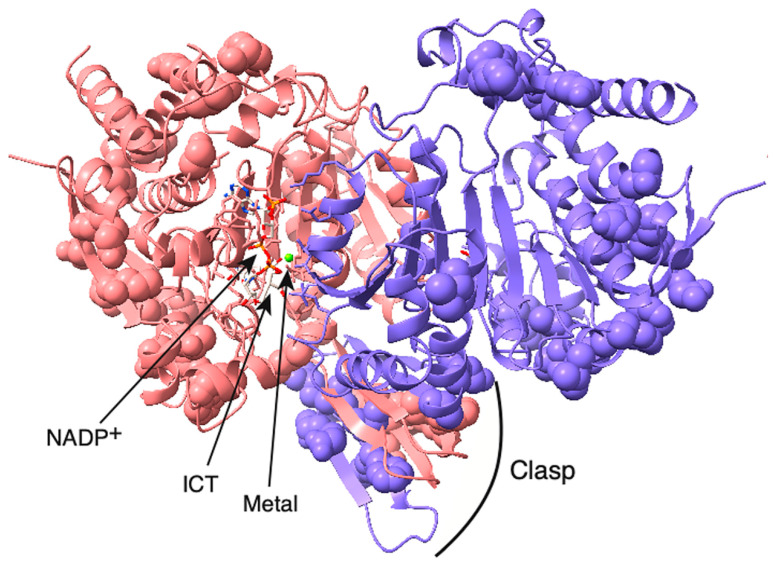
Amino acids in IDH-1 with different predicted flexibility compared to human IDH1. The amino acids with different flexibilities determined using MEDUSA and indicated in Figure 6 are shown here in space-filling view. The clasp region is at the bottom of the structure, and the two active sites are on the top left and right. This structural model is the same homology model as shown in Figure 3, and it is in the same orientation. One subunit is pink, and the other is purple. The substrates are colored by element. The green sphere is the metal.

The IDH1 enzyme undergoes a large conformational change upon ligand binding [39], so the flexibility of the enzyme is important for its ability to close productively over the substrates. Therefore, amino acid changes in the hinge regions may affect the enzyme’s activity. To investigate the protein flexibility, we submitted the human IDH1 and *C. elegans* IDH-1 sequences to the Multiclass flExibility preDiction from seqUenceS of Amino acids (MEDUSA) server (https://www.dsimb.inserm.fr/MEDUSA/, accessed on 25 September 2024) [48] (Figure 6 and Supplementary Figure S3: MEDUSA analysis of IDH1 and IDH-1). We found that the flexibility of the amino acids in most of the two proteins were similar, but there were two regions of difference. The amino acids with different predicted flexibility are shown in the IDH-1 homology model in space-filling view in Figure 7. The two subunits of the human IDH1 dimer are held together on the bottom by a clasp region (involving amino acids 140–185 in IDH-1) with two pairs of beta sheets that wrap around each other, and these are important for the conformational changes [39,42]. This region in IDH-1 is more flexible, in general, compared to human IDH1. In contrast, some amino acids surrounding the active sites (amino acids 15–68 in IDH-1) are generally more flexible in human IDH1.

Another possible reason for the lower K_M_ for αKG for R133H is because we used MnCl_2_ instead of MgCl_2_ in our enzyme assays because IDH-1 preferred Mn^2+^ over Mg^2+^. Others have reported a similar preference for Mn^2+^ over Mg^2+^ for the human enzyme, and some of these results are shown in Table 2. Specifically, the k_cat_ values reported in the same studies were all higher in the presence of Mn^2+^ compared to Mg^2+^. The K_M_ values for ICT for wild-type IDH1 and R132H were not much lower in the presence of Mn^2+^ compared to Mg^2+^, but the K_M_ value for αKG for the R132H mutant was ~4-fold lower (Table 2).

### 2.4. 2-Hydroxyglutarate Production

**Figure 8:**
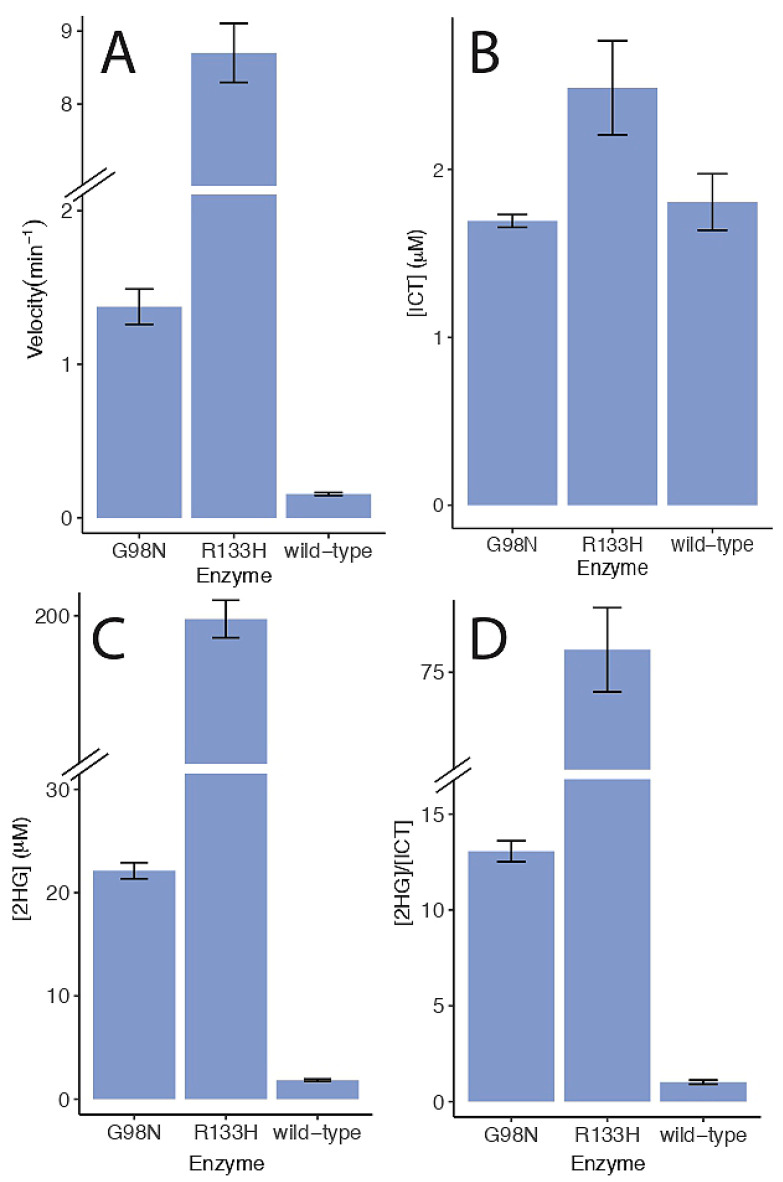
Reverse reaction rates and 2-HG production. (**A**) Reaction progress was measured discontinuously at 340 nM, and the velocities are shown. (**B**) The [ICT] produced in each reaction is shown. (**C**) The [2HG] produced in each reaction is shown. (**D**) The ratio of 2HG to ICT produced is shown. The values in (**B**,**C**) were adjusted for the length of time for each reaction (wild-type: 60 min, G98N: 30 min, R133H: 20 min). Reactions were carried out at 22 °C and contained 50 mM Tris pH 8.0, 1 mM MnCl_2_, 50 μM NADPH, 200 μM αKG, and 230 nM enzyme. Each reaction was stopped using 2 mM EDTA and processed for LC-MS as described in the methods. Each set of data shows the average and standard error. Note that the *y*-axis in (**A**,**C**,**D**) has a break as indicated. The G98N velocity data was done in duplicate, and all the other data sets were done in triplicate.

To determine if the IDH-1 mutants produced 2-HG, we analyzed reactions with LC-MS. In our steady-state kinetic experiments lasting less than a few minutes, we were not able to detect the reverse reaction for wild-type IDH-1 or the G98N mutant. For the LC-MS experiments, the reverse reactions were performed over a longer time, allowing us to determine a reverse reaction rate for the three enzymes under one set of conditions (Figure 8A). We found that wild-type IDH-1 had a very low reverse reaction rate and produced little ICT or 2HG (Figure 8A–C). The G98N and R133H mutants had increasing reverse reaction rates (Figure 8A). While both enzymes were capable of producing small amounts of ICT, they produced much more 2HG compared to ICT (Figure 8D). The concentrations of ICT shown in Figure 8B and the concentrations of 2HG shown in Figure 8C were adjusted for the length of time for each reverse reaction. In our preliminary experiments, we observed that the R133H reverse reaction was not linear after 20 min as it approached equilibrium, and this is why we stopped that reaction at 20 min. The G98N reaction was linear up to 30 min, the wild-type reaction was linear up to 60 min, and we did not test longer time points for these two enzymes.

## 3. Discussion

### 3.1. Enzyme Kinetics Results

Our enzyme kinetic results for wild-type *C. elegans* IDH-1 were similar to those found for human IDH1. Others measured a similar pH profile for human IDH1 and porcine cytoplasmic IDH1, with the velocity increasing from pH 5–7 and leveling off between pH 7.5–8 [43,49]. Table 1 and Table 2 showed that the K_M_ values for ICT and NADP^+^ were similar to those for human IDH1. There was some variation in the published k_cat_ values for human IDH1, and the k_cat_ for IDH-1 was similar to the higher published values (Table 1 and Table 2).

For *C. elegans* wild-type IDH-1, we measured a velocity of 0.16 ± 0.01 min^−1^ in the reverse reactions we used for our LC-MS measurements (Figure 8A). These reactions were performed with 200 μM αKG. Published values for the k_cat_ and K_M_ for ICT for wild-type human IDH1 for the reverse reaction varied widely (Table 2), and our velocity for *C. elegans* IDH-1 was on the low end. We measured the same amount of 2HG as ICT produced by IDH-1 for the reverse reaction (Figure 8). This is likely because we did not add bicarbonate to our reactions as an extra source of CO_2_. Others have found that human wild-type IDH1 homodimers could produce 2HG [30,47], but it produced more ICT in the reverse reaction if bicarbonate was added to the reaction [30,50].

The *C. elegans* G98N mutant had some differences compared to human G97N (Table 1 and Table 2). In particular, the forward k_cat_ was higher (Table 1 and Table 2). We measured a reverse velocity of 1.4 ± 0.1 min^−1^ (Figure 8A) at 200 μM αKG. This is the same order of magnitude as the reverse k_cat_ for human G97N (Table 2). It’s possible that the increased flexibility of the clasp region in IDH-1 allows the G98N mutant to function better in the forward direction than human G97N despite any steric hindrance from the N98-I215 interaction.

The *C. elegans* R133H mutant had a similar K_M_ for ICT and forward k_cat_ compared to human R132H (Table 1 and Table 2). However, the *C. elegans* R133H k_cat_ for the reverse reaction was lower, as was the K_M_ for αKG, compared to the human R132H mutant. In the active site of human IDH1, ICT binds to Arg 100, Arg 109, and Arg 132 as well as to the metal [39]. Since αKG is smaller with one less carboxyl group, it only binds to Arg 100, Arg 109, and the metal in the R132H mutant [30]. This suggests that αKG binding to IDH1 and *C. elegans* IDH-1 may be more affected by the choice of metal than ICT because a higher percentage of the bonds between αKG and the enzyme involve the metal. In addition, NADP^+^ doesn’t interact with the metal, so the binding of NADP^+^ and NADPH may be less affected by the choice of metal in the reactions.

### 3.2. Implications for In Vivo Experiments

In order for *C. elegans* to be a good model system, mutant IDH-1 would need to produce 2HG in vivo. The cytoplasmic concentration of αKG in a variety of human cancer cell lines is 20–40 μM [51]. Since this is above the K_M_ for αKG that we measured for the R133H mutant (Table 1), it suggests that this mutant would produce 2HG in vivo. In experiments with whole worms, a recent study of metabolism in *C. elegans* found that a genomic R133H mutation in IDH-1 did produce 2HG [38]. (These authors based their amino acid numbering scheme on a different isoform of IDH-1, but based on the homology figure shown in the article, they mutated R133 of isoform a.) These authors also left a wild-type copy of IDH-1 in the genome.

One issue related to human cancers with R132H mutations is that numerous studies have shown that more 2HG is produced if the cells are heterozygous (wild-type IDH1/R132H) compared to cells that are homozygous R132H [52,53] One possible explanation is that a mixture of homodimeric and heterodimeric enzymes form. In vitro experiments with heterodimers found that the human R132H homodimer produced more 2HG than a wild-type:R132H heterodimer at pH 6 [50]. One complicating factor is that the amount of 2HG produced by heterodimers in vitro depended on pH. In a mixture of both wild-type and R132 subunits, a human wild-type:R132H heterodimer formed more easily at pH 7.0 compared to pH 7.5. Therefore, the heterodimer formed more 2HG at pH 7 compared to pH 7.5, but the heterodimer still only produced similar amounts of 2HG at pH 7.0 as a R132H homodimer. [54]. These results suggest that heterodimeric IDH1 is not required to produce more 2HG in heterozygotes.

Another factor affecting how much 2HG is produced in vivo is the availability of αKG. Human R132H has a high K_M_ for αKG, and since human R132H has such a low k_cat_ in the forward direction, a wild-type copy of IDH1 may be required to produce enough αKG to allow the R132H reverse reaction that produces 2HG to occur in the cytoplasm [53]. Another source of αKG is glutamine, and in heterozygous human cells with a R132G mutation with ^13^C-labeled glutamine added to the media, most of the 2HG was produced from glutamine [25]. Since the *C. elegans* R133H mutant has a lower K_M_ for αKG compared to human R132H, it’s possible that the production of 2HG by the R133H mutant is less reliant on a wild-type IDH-1 enzyme being present.

In a study of different human IDH1 mutants at position R132, the IDH1 mutations with lower K_M_ values for αKG produced more 2HG [55]. The lowest K_M_ for αKG was about 10 μM for a R132L mutant, which was similar to the K_M_ we measured for R133H (Table 1). Cells overexpressing a human R132L mutant contained twice as much 2HG compared to cells overexpressing a R132H mutant [55]. The study in heterozygous *C. elegans* found that a R133C mutation resulted in worms with more 2HG than a R133H mutation [38]. We found that the G98N mutant produced less 2HG than a R133H mutant. These results suggest that the amount of 2HG in *C. elegans* could be controlled by the specific IDH-1 mutation used.

As mentioned in the introduction, the presence of 2HG in cells inhibits histone lysine demethylases and the TET oxygenases that are required for DNA demethylation (reviewed in [20,21,22,23]). Most histone modifications in human cells are conserved in *C. elegans* [56,57]. *C. elegans* does not have methylation on position 5 of cytosine in DNA [58]. However, it does have another epigenetic mark, methylation at N^6^ of adenosine in DNA (6 mA) [59] as well as a conserved αKG-dependent AlkB-family DNA demethylase, NMAD-1, that acts on 6 mA [59,60]. Therefore, the effects of 2HG inhibition of histone demethylases and at least one DNA demethylase could be studied in *C. elegans*.

### 3.3. Metal Binding

The metal binding site in IDH1 has been frequently investigated because ICT binding to IDH1 requires a divalent metal [61]. Studies going back to 1972 have shown that IDH enzymes from many species have a preference for Mn^2+^ over Mg^2+^ [31,61]. However, since the free cytosolic [Mg^2+^] is 0.25–1 mM [62], some authors argued that human IDH1 is likely predominantly bound to Mg^2+^. Therefore, many in vitro studies of IDH1 have only involved reactions with Mg^2+^ (Table 2).

Numerous studies of Mn^2+^ in *C. elegans* have been done because soaking worms in millimolar concentrations of Mn^2+^ induces neurodegeneration of *C. elegans*, and this is a model for Parkinsons Disease [63,64,65]. Two different studies found that worms grown on standard solid media (nematode growth medium agar plates with bacteria as the food source) in the absence of added Mn^2+^ had a similar Mn^2+^ content as worms grown in the same way but treated with 2.5–3.0 mM MnCl_2_ for 30 min [64,66]. The divalent metal transporter SMF-3 is required for Mn^2+^ update in *C. elegans* [66]. Therefore, while the specific cytoplasmic concentration of Mn^2+^ in *C. elegans* hasn’t been determined, worms grown in the absence of added Mn^2+^ contained measurable quantities of the ion. We found that IDH-1 had a 3-fold preference for Mn^2+^ and a low K_M_ for Mn^2+^ (Table 1), and this K_M_ is over 2 orders of magnitude lower than the concentration of MnCl_2_ used outside the worm to induce neurodegeneration. Nevertheless, *C. elegans* IDH-1 could use Mg^2+^, and Mg^2+^ is found in higher concentrations in the cytoplasm than Mn^2+^, so it seems likely that IDH-1 binds to both Mg^2+^ and Mn^2+^ in vivo.

### 3.4. Potential for C. elegans as a Cancer Model

Since there is a high sequence conservation between *C. elegans* IDH-1 and human IDH1, and since the *C. elegans* G98N and R133H mutants produce 2HG, it appears that *C. elegans* could be a good model system for studying the effects of 2HG on proteins, cells and organs. It might also be a good system for studying ways to reduce the harms caused by excess 2HG. However, since the enzyme kinetic results were different between G98N and R133H and the corresponding human enzymes, it seems unlikely that *C. elegans* would serve as a good model specifically for human cancers involving IDH1 mutations or for developing drugs that bind to IDH1.

## 4. Materials and Methods

### 4.1. Sequence Analysis

There are three transcripts predicted from the *idh-1* locus. Based on RNASeq data in WormBase (wormbase.org), isoform a is highly expressed. The protein sequence for *C. elegans* IDH-1, isoform a (F59B8.2a, NP_001255393.1) was aligned with human IDH1 (NP_001269316.1) using EMBOSS Needle at EMBL-EBI [67,68] (https://www.ebi.ac.uk/jdispatcher/psa/emboss_needle, accessed on 2 May 2024). The mutation sites were identified using the homologous regions around the human mutation sites. The protein sequences for Q9YE81_AERPE, *Aeropyrum pernix*; IDH_BACSU, *Bacillus subtilis*; IDH_ECOLI, *Escherichia coli*; Q63WJ4_BURPS, *Burkholderia pseudomallei*, Q9X0N2_THEMA, *Thermotoga maritima*; IDHP_YEAST, *Saccharomyces cerevisiae*; IDH1_MYCTU, *Mycobacterium tuberculosis*; Q21032_CAEEL, *C. elegans*; IDHC_MOUSE, *Mus musculus*; IDHC_HUMAN, *Homo sapiens*; I3LDC7_PIG, *Sus scrofa*, and IDH_THET8, *Thermus thermophilus*, were aligned using Clustal Omega, accessed 7 August 2024 [69], and the alignments in Figure 2 and Figure S1 were made using JalView (Ver. 2.11) [70].

### 4.2. Plasmid Construction

The *idh-1* gene was amplified from a mixed population of wild type (Bristol N2) *C. elegans* (NCBI: txid6239) cDNA using primers idh-1_for (5′-TATAACCATGGCTGCTCAAAAGATCCAAGGAGGAGAC-3′) and idh-1_rev (5′-GGGCAACTAGTGCATCTCCCGTGATGCAATGGGCTTGCTTCTTGGCG-3′). Primers were obtained from Integrated DNA Technologies (IDT, Coralville, IA, USA), and restriction enzymes were purchased from New England Biolabs (NEB, Ipswich, MA, USA). The PCR product was cut with NcoI and SpeI and ligated into the plasmid pTXB3 (NEB) that was also cut with NcoI and SpeI to produce the IDH1_WT plasmid. The resulting protein has an intein cleavage site and a chitin-binding domain (CBD) on the C-terminus. After cleavage, the protein has the authentic wild-type IDH-1 sequence.

The IDH1_WT plasmid was mutated using PCR. The G98N mutant (IDH1_G98N plasmid) was made using two overlapping and diverging PCR primers. The sequences were idh-1_G98Nfor (5′-GTCACCAAACaacACTATCCGTAACATTCTTGGAGGAAC-3′) and idh-1_G98Nrev (5′-GATAGTgttGTTTGGTGACTTCCACATTTTCTTaAGCTTGAAC-3′). The resulting PCR product contained the entire plasmid with the GGA-AAC mutation and an additional silent mutation that added a HindIII site. The R133H mutation (IDH1_R133H plasmid) was made by using two sets of primers to amplify the plasmid in two pieces using PCR and then assembling these two fragments into the plasmid using HiFi Assembly (NEB). One PCR reaction used the primers fragmentA_for (5′-CTC GCC GAA ACG TTT GGT GGC GGG ACC AGT GAC GAA GGC T-3′) and fragmentA_rev (5′-ACA AAG TCG GTG GCC TTG TAT TGA TCA GCA TGc GCG TGA tGT CCA ATG ATG ATT GGC TTC-3′), and the second reaction used the primers fragment B_for (5′-CAA CAC CTG GTC GAA GCC AAT CAT CAT TGG ACa TCA CGC gCA TGC TGA TCA ATA CAA GGC-3′) and fragmentB_rev (5′-AGC CTT CGT CAC TGG TCC CGC CAC CAA ACG TTT CGG CGA G-3′). The resulting plasmid has a silent mutation to add a SphI site in addition to the desired mutation (CGT-CAT). All three plasmid sequences were verified using dideoxynucleotide sequencing. Subsequently the plasmids were sent to Addgene where they were sequenced again, and they are available at https://www.addgene.org/Katherine_Walstrom/.

### 4.3. Recombinant Enzyme Expression and Purification

All recombinant enzymes were expressed in freshly transformed Rosetta (DE3) or T7 Express *E. coli* cells (NEB) grown in terrific broth (1.2% tryptone, 2.4% yeast extract, 0.4% glycerol, 0.017 M KH_2_PO_4_, 0.072 M K_2_HPO_4_) and 0.1 mg/mL ampicillin to a density of OD600 = 0.4–0.5. The proteins were expressed using 0.01 mM IPTG in cells grown for 16 h at 20–25 °C. The cells were pelleted and stored at −20 °C until the enzymes were purified. Both enzymes were expressed as a fusion protein with a self-cleaving intein and a chitin-binding domain on the C-terminus. The fusion proteins were purified on chitin resin using the IMPACT^TM^ expression system (NEB) according to the manufacturer’s instructions. Cleavage was performed over 20–24 h at 4–6 °C. Protein samples were analyzed using 10% Laemmli SDS-PAGE gels stained with Coomassie blue [71]. (See Supplementary Figure S4: SDS-PAGE gels of purified RHA-1 proteins). Enzyme fractions with comparable purity were combined and dialyzed into dialysis buffer (20 mM Tris-HCl, pH 7.5, 0.2 M KCl, and 40% glycerol) before storage at −20 °C. Protein concentrations were determined using a Coomassie Plus (Bradford) Assay kit (Pierce^TM^, ThemoFisher, Waltham, MA, USA) and bovine serum albumin (Sigma) as the standard. The concentrations of the dialyzed enzymes in mg/mL were wild-type IDH-1, 1.36 ± 0.06 (prep. 1), 1.14 ± 0.01 (prep. 2); G98N, 0.31 ± 0.01; and R133H, 0.44 ± 0.01 (prep. 1), 1.05 ± 0.03 (prep. 2).

In our preliminary experiments, we found that wild-type IDH-1 was very stable, but the G98N and R133H mutants lost activity over a few months. The data shown here were collected shortly after protein purification, and the enzyme activity was checked each day to determine if there was a loss of activity.

### 4.4. Steady-State Enzyme Kinetics Measurements

The reagents were purchased from Sigma-Aldrich (St. Louis, MO, USA) or ThermoFisher (Waltham, MA, USA). Substrate stock solutions were prepared fresh or stored at −20 °C for no more than two weeks. The MnCl_2_ stock solution was made fresh at least every week to reduce the oxidation of Mn^2+^. The reaction solutions were prepared fresh each day from the stock solutions. The reaction conditions for wild-type IDH-1 were optimized as described in the Section 2, and enzyme kinetics assays were performed in assay buffer (0.05 M Tris buffer, pH 8.0, 1 mM MnCl_2_) that had been pre-incubated to 25 °C. The enzyme stocks were kept on ice before being diluted 1:100 or 1:200 into assay buffer at 25 °C. To measure the appearance or disappearance of NADPH, the solutions were monitored continuously at 340 nm for 30–60 s using an Olis-upgraded Cary 14 (Olis, Athens, GA, USA) or a Jasco V-750 spectrophotometer (Oklahoma City, OK, USA), and the slopes of the lines were measured using the spectrophotometer software (Olis GlobalWorks, Jasco Spectral Manager 2). Experiments with ICT varying from 0–200 μM included 50 μM NADP^+^ for the wild-type and G98N reactions. For the R133H reactions, ICT varied from 0 to 10 mM and the reactions included 5 μM NADP^+^. Experiments with NADP^+^ varying from 0–100 μM included 2 mM ICT for wild-type IDH-1 and 200 μM ICT for G98N. Experiments with αKG varying from 0 to 250 μM included 5 μM NADPH, and experiments with NADPH varying from 0 to 50 μM included 100 μM αKG. Enzyme concentrations for these reactions were 1–7 nM for wild-type IDH-1, 34 nM for G98N, and 100–230 nM for R133H. All enzyme kinetics parameters were determined from non-linear least-squares fits of the data to the Michaelis-Menten equation using RStudio (version 2025.05.0+496). The statistics related to the fits are shown in Table S1. The k_cat_ values were calculated based on the V_max_ values for each curve and the amount of enzyme used. The graphs show individual data points, and the standard errors are reported in the tables and text.

### 4.5. Liquid Chromatography—Mass Spectrometry (LC-MS) Procedure

The enzyme reactions were performed at 22 °C and contained 50 mM Tris pH 8.0, 1 mM MnCl_2_, 50 μM NADPH, 200 μM αKG, and 230 nM enzyme. The wild-type IDH-1 reaction ran for 60 min, the G98N reaction ran for 30 min, and the R133H reaction ran for 20 min. The absorbance at 340 nm was monitored every 5–10 min during the reactions. At the final time point, the reactions were put on ice, and then 2 mM EDTA was added to stop the reactions and prevent oxidation of Mn^2+^. The samples were centrifuged through a 10K microcon (Merck, MilliporeSigma, Burlington, MA, USA) filter at 13K× *g* for 75 min to remove protein from the samples. Three filtered samples for each enzyme were prepared.

Tandem mass spectrometry was performed in negative mode to analyze isocitrate (ICT) and 2-hydroxyglutarate (2HG) by multiple-reaction monitoring (MRM) using the Agilent LC-MS QqQ 6460 equipped with an Agilent Jet Stream (AJS) technology electrospray ionization (ESI) source (Agilent, Santa Clara, CA, USA). The nitrogen gas temperature was 300 °C, the gas flow rate at 10 L/min, the nebulizer at 20 psi, and the capillary voltage at 3500 V. Data acquisition was controlled by Agilent Mass hunter acquisition software B05 version, and the MRM transitions (precursor ion—>product ion) of 2HG and ICT were optimized at a fragmentor voltage of 56 V and a collision energy of 9 V for 2-HG *m*/*z* 147.03—>129.00, and at a fragmentor voltage of 60 V and a collision energy of 13 V for ICT *m*/*z* 191.02—>111.00.

The chromatography for each injection was realized on a Kinetex Polar C18 column (2.6 μm, 100 × 4.6 mm; Phenomenex, Torrance, CA, USA) held at 35 °C. The injection volume was 5 μL, and the flow rate was 0.60 mL/min under LC-MS grade aqueous acetonitrile gradient (+0.1% formic acid) condition of 2% over 2 min, 2 to 95% over 3 min, 95% over 2 min, 95 to 5% over 0.5 min, 2% over 0.5 min with a 2 min equilibration time.

Quantification of the molecules was done with Agilent MassHunter Software for Quantitative Analysis, version B05. Standard samples were prepared in 50 mM Tris pH 8.0 buffer in concentrations from 0.1 to 50 μM ICT and 0.1 to 100 μM 2HG. Blank samples contained 50 mM Tris pH 8.0. The peak areas were determined, and the sample areas were compared to the standards using linear standard curves. (The LC-MS quantification results are provided in Supplementary Figure S5: LC-MS quantification of ICT and 2HG.)

### 4.6. Protein Molecular Modeling and Structure Analysis

The IDH-1 wild-type, G98N, and R133H sequences were submitted to SWISS-MODEL (accessed 29 August and 11 September 2024) [72,73], and the predicted structures were built using ProMod3 (version 3.4.1, [74]). The templates were the crystal structures 6BL0 [75] for wild-type IDH-1, 4L03 [30] for G98N, and 5YZH [76] for R133H. The models had GMQE and QMEANDisCo Global scores [77] of 0.89 and 0.84 ± 0.05, respectively, for wild-type IDH-1; 0.9 and 0.84 ± 0.05 for G98N; and 0.89 and 0.84 ± 0.05 for R133H. ChimeraX 1.9 [78] was used to produce the structure figures. The IDH-1 wild-type sequence was also submitted to the AlphaFold 3 Server (accessed 2 January 2025) [79]. The CIF and PDB files for the AlphaFold model and all of the homology models are in Supplementary Structures S1–S4.

Generative artificial intelligence was not used in any way in the preparation of this manuscript or the figures.

## 5. Conclusions

We characterized wild-type *C. elegans* IDH-1 and two mutants, G98N and R133H, that are homologous to human IDH1 mutants that are prevalent in cancer cells. We found that the *C. elegans* proteins have quite a few similarities to the human version, but the G98N was more active with ICT as a substrate compared to human G97N. We also found that the R133H mutant had a lower K_M_ for αKG compared to the human R132H mutant. We discussed the implications for these results and the possibility and potential complications of using *C. elegans* as a model system for studying 2HG-induced effects on cells.

## Figures and Tables

**Table 1:** Steady-state Enzyme Kinetics Results for IDH-1 at 25 °C.

Enzyme	Varied Substrate	k_cat_, min^−1^	K_M_, μM	k_cat_/K_M_, μM^−1^ min^−1^
Wild-type IDH-1	ICT	2510 ± 70	6.9 ± 0.8	360 ± 40
Wild-type IDH-1	NADP^+^	1260 ± 70	3.3 ± 0.5	380 ± 60
Wild-type IDH-1 *	Mn^2+^	1590 ± 90	9 ± 2	176 ± 30
G98N	ICT	190 ± 6	13 ± 1	15 ± 1
G98N	NADP^+^	104 ± 3	2.8 ± 0.3	37 ± 4
R133H	ICT	11.7 ± 0.6	2160 ± 250	0.0054 ± 0.0007
R133H	αKG	18.1 ± 0.7	14 ± 2	1.3 ± 0.2
R133H	NADPH	16.7 ± 0.7	1.7 ± 0.2	10 ± 1

* The steady-state fit of the Mn^2+^ data required a constant added to the [MnCl_2_] term of 0.6 ± 0.6 μM. See text.

**Table 2:** Published Steady-state Enzyme Kinetics Results for human IDH1 at 25 °C.

Enzyme	Varied Substrate	Metal	k_cat_, min^−1^	K_M_, μM	k_cat_/K_M_, μM^−1^ min^−1^	Citation
Wild-type IDH1	ICT	Mg^2+^	517 ± 9	5.0 ± 0.3	103 ± 6	[30]
Wild-type IDH1	ICT	Mg^2+^	749	7	107	[47]
Wild-type IDH1	ICT	Mg^2+^	3000 ± 240	38 ± 6	78 ± 18	[31]
Wild-type IDH1	ICT	Mn^2+^	4860 ± 660	35 ± 4	138 ± 36	[31]
Wild-type IDH1	NADP^+^	Mg^2+^	3000 ± 240	27 ± 2	114 ± 18	[31]
Wild-type IDH1	NADP^+^	Mg^2+^	517 ± 9	6.2 ± 0.3	83 ± 4	[30]
Wild-type IDH1	Mn^2+^	Mn^2+^	4860 ± 660	2.0 ± 0.3	2460 ± 720	[31]
Wild-type IDH1	aKG	Mg^2+^	0.07	43	0.0015	[47]
Wild-type IDH1	aKG	Mg^2+^	134 ± 5	138 ± 38	0.97 ± 0.27	[30]
Wild-type IDH1	aKG	Mg^2+^	126 ± 6	1100 ± 300	0.11 ± 0.04	[31]
G97N	ICT	Mg^2+^	6.1 ± 0.3	0.46 ± 0.07	13 ± 2	[30]
G97N	NADP^+^	Mg^2+^	6.1 ± 0.3	0.06 ± 0.03	10.2 ± 0.5	[30]
G97N	aKG	Mg^2+^	2.52 ± 0.05	295 ± 13	0.0090 ± 0.0004	[30]
R132H	ICT	Mg^2+^	7.7 ± 0.4	6600 ± 860	0.0012 ± 0.0002	[30]
R132H	ICT	Mg^2+^	6.0 ± 0.6	257 ± 50	0.023 ± 0.007	[31]
R132H	ICT	Mn^2+^	47 ± 5	219 ± 35	0.22 ± 0.05	[31]
R132H	αKG	Mg^2+^	42.8 ± 0.7	1080 ± 67	0.040 ± 0.003	[30]
R132H	αKG	Mg^2+^	37 ± 4	652 ± 116	0.06 ± 0.02	[31]
R132H	αKG	Mn^2+^	62 ± 3	175 ± 26	0.35 ± 0.07	[31]
R132H	NADPH	Mg^2+^	37 ± 4	15 ± 2	2.5 ± 0.6	[31]
R132H	NADPH	Mg^2+^	45 ± 0.9	0.43 ± 0.04	105 ± 10	[30]

## Data Availability

Data is contained within the article and Supplementary Materials.

## Supplementary Materials

The following supporting information can be downloaded at: https://www.mdpi.com/article/10.3390/ijms26178238/s1.

## Abbreviations

The following abbreviations are used in this manuscript:

αKG	α-ketoglutarate
CBD	chitin binding domain
G97N	mutation in IDH1
G98N	mutation in IDH-1
ICT	isocitrate
IDH1	human cytoplasmic isocitrate dehydrogenase
IDH-1	*C. elegans* cytoplasmic isocitrate dehydrogenase
LC-MS	liquid chromatography—mass spectrometry
6 mA	methylation at N^6^ of adenosine
NAD^+^	oxidized nicotinamide adenine dinucleotide
NADP^+^	oxidized nicotinamide adenine dinucleotide phosphate
NADPH	reduced nicotinamide adenine dinucleotide phosphate
R132H	mutation in IDH1
R133H	mutation in IDH-1
TET	ten-eleven translocation (TET) oxygenase
TCA	tricarboxylic acid
