# Supplementary material for: C. elegans Cytoplasmic Isocitrate Dehydrogenase Neomorphic G98N and R133H Mutants Produce the Oncometabolite 2-Hydroxyglutarate
Source: Int J Mol Sci. 2025 Aug 25;26(17):8238. doi: 10.3390/ijms26178238 (PMC12427979; doi:10.3390/ijms26178238)
Supplement: Supplementary file 1 [file ijms-26-08238-s001.zip › FigureS1.pdf]

Figure S1: Sequence Analysis of IDH-1

A

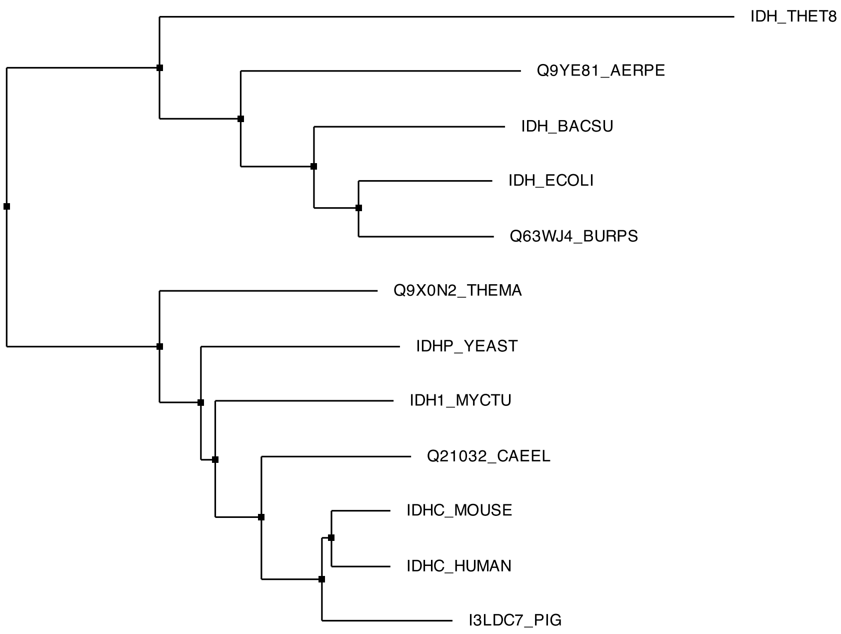

B

	T. MAR.	*S. CER.*	*M. TUB.*	*C. ELE.*	*M. MUS.*	*H. SAP.*	*S. SCRO.*	*T. THERM.*	*A. PER.*	*B. SUB.*	*E. COLI*	*B. PSEUD.*
**Q9X0N2_THEMA**	100	56.2	55.5	51.1	53.1	53.4	52.4	22.1	18.6	23.6	23.4	24.2
**IDHP_YEAST**	56.2	100	61.9	62.8	65.3	65.5	62.8	21.7	18.0	19.8	17.2	19.7
**IDH1_MYCTU**	55.5	61.9	100	64.9	65.0	65.5	64.3	21.8	20.7	20	21.9	19.9
**Q21032_CAEEL**	51.1	62.8	64.9	100	76.5	75.8	75.9	21.3	18.1	20	19.8	20
**IDHC_MOUSE**	53.1	65.3	65.0	76.5	100	95.7	95.2	22.0	19.8	20	18.1	18.9
**IDHC_HUMAN**	53.4	65.5	65.5	75.8	95.7	100	95.7	21.7	19.3	19.4	17.6	18.9
**I3LDC7_PIG**	52.4	62.8	64.3	75.8	95.2	95.7	100	21.6	18.6	19.4	17.8	18.7
**IDH_THET8**	22.1	21.7	21.8	21.3	22.0	21.7	21.6	100	37.1	39.2	39.7	36.6
**Q9YE81_AERPE**	18.6	18.0	20.7	18.1	19.8	19.3	18.6	37.1	100	48.9	48.2	44.6
**IDH_BACSU**	23.6	19.8	20	20	20	19.4	19.4	39.2	48.9	100	68.8	67.4
**IDH_ECOLI**	23.4	17.2	21.9	19.8	18.1	17.6	17.8	39.7	48.2	68.8	100	74.8
**Q63WJ4_BURPS**	24.2	19.7	19.9	20	18.9	18.9	18.7	36.6	44.6	67.4	74.8	100

C

```
Q9X0N2_THEMA/1-399    1 -------------------------------------------------MEKVKVKNPIV-ELDGDEMARVMWKMI  26
IDHP_YEAST/1-428      1 ------------------------------MSMLSRRL---FSTSRLAAFSKIKVKQPVV-ELDGDEMTRIIWDKI  42
IDH1_MYCTU/1-409      1 ----------------------------------------------MSNAPKIKVSGPVV-ELDGDEMTRVIWKLI  29
Q21032_CAEEL/1-412    1 ------------------------------------------------MAAQKIQGGDIV-EMQGDEMTRIIWDLI  27
IDHC_MOUSE/1-414      1 -------------------------------------------------MSRKIQGGSVV-EMQGDEMTRIIWELI  26
IDHC_HUMAN/1-414      1 -------------------------------------------------MSKKISGGSVV-EMQGDEMTRIIWELI  26
I3LDC7_PIG/1-463      1 MTIKSKRVSGVPVVAQWLTSPTRNHEVAGSISGLAQWKDPALLFIKAKMSQKIQGGSVV-EMQGDEMTRIIWELI  75
IDH_THET8/1-496       1 ---------------MPLITTETGKK----MHVL-------------E------DGRKLITVIPGDGIGPECVEAT  38
Q9YE81_AERPE/1-432    1 -------MASPPCTTEELS-PPPGGS----LVEY-------------SGGSLRVPDNPVVAFIRGDGVGPEVVESA  51
IDH_BACSU/1-423       1 --------------------MAQGEK----ITVS--------------NGVLNVPNNPIIPFIEGDGTGPDIWNAA  38
IDH_ECOLI/1-416       1 -------------MESKVVVPAQGKK----ITLQ--------------NGKLNVPENPIIPYIEGDGIGVDVTPAM  45
Q63WJ4_BURPS/1-419    1 ------------MPYQHIKVPEGGDK----ITVN-------------KDFSLNVSDQPIIPYIEGDGTGFDITPVM  47

Q9X0N2_THEMA/1-399   27 KEKLI----LPY-LDIQLVYFDL--GIK--KRDETDDQITIEAAKAIKKYGVGVKCATITPDAERVKEYNLKKAWK  93
IDHP_YEAST/1-428     43 KKKLI----LPY-LDVDLKYYDL--SVE--SRDATSDKITQDAAEAIKKYGVGIKCATITPDEARVKEFNLHKMWK 109
IDH1_MYCTU/1-409     30 KDMLI----LPY-LDIRLDYYDL--GIE--HRDATDDQVTIDAAYAIKKHGVGVKCATITPDEARVEEFNLKKMWL  96
Q21032_CAEEL/1-412   28 KEKLI----LPY-VDLNVHFFDL--GIE--HRDATDDQVTIDAANATLKYNVAVKCATITPDEARVEEFKLKKMWK  94
IDHC_MOUSE/1-414     27 KEKLI----LPY-VELDLHSYDL--GIE--NRDATNDQVTKDAAEAIKKYNVGVKCATITPDEKRVEEFKLKQMWK  93
IDHC_HUMAN/1-414     27 KEKLI----FPY-VELDLHSYDL--GIE--NRDATNDQVTKDAAEAIKKHNVGVKCATITPDEKRVEEFKLKQMWK  93
I3LDC7_PIG/1-463     76 KEKLI----FPY-VELDLHSYDL--GIE--NRDATNDQVTKDAAEAIKKYNVGVKCATITPDEKRVEEFKLKQMWK 142
IDH_THET8/1-496      39 LKVLEAAKAPLAY--------EVREAGASVFRRGIASGVPQETIESIRKTRVVLKGPLETPVGYG---------EK  97
Q9YE81_AERPE/1-432   52 LKVVDAAVKKVYGGSRRIVWWELLAGHLAREKC--GELLPKATLEGIRLARVALKGPLETPVGTG---------YR 116
IDH_BACSU/1-423      39 SKVLEAAVEKAYKGEKKITWKEVYAGEKAYNKT--GEWLPAETLDVIREYFIAIKGPLTTPVGGG---------IR 103
IDH_ECOLI/1-416      46 LKVVDAAVEKAYKGERKISWMEIYTGEKSTQVYGQDVWLPAETLDLIREYRVAIKGPLTTPVGGG---------IR 112
Q63WJ4_BURPS/1-419   48 IKVVDAAVEKAYGGKKKIHWMEIYAGEKATKVYGPDVWLPEETLQVLKEYVVSIKGPLTTPVGGG---------IR 114

Q9X0N2_THEMA/1-399   94 SPNATIRAYLDGTVFRKPIM-VKNVPPLVKR-WKKPIIIGRHAYGDIYNAVEAKVEGPAEVELVVRNKENK----- 162
IDHP_YEAST/1-428    110 SPNGTIRNILGGTVFREPIV-IPRIPRLVPR-WEKPIIIGRHAHGDQYKATDTLIPGPGSLELVYKPSDPTTAQPQ 183
IDH1_MYCTU/1-409     97 SPNGTIRNILGGTIFREPIV-ISNVPRLVPG-WTKPIVIGRHAFGDQYRATNFKVDQPGTVTLTFTPADGSAPI-- 168
Q21032_CAEEL/1-412   95 SPNGTIRNILGGTVFREPII-VKNVPRLVNT-WSKPIIIGRHAHADQYKATDFVVPGAGKLEIKFVSADGTQTI-- 166
IDHC_MOUSE/1-414     94 SPNGTIRNILGGTVFREAII-CKNIPRLVTG-WWKPIIIGRHAYGDQYRATDFVVPGPGKVEITYTPKDGTQKV-- 165
IDHC_HUMAN/1-414     94 SPNGTIRNILGGTVFREAII-CKNIPRLVSG-WWKPIIIGRHAYGDQYRATDFVVPGPGKVEITYTPSDGTQKV-- 165
I3LDC7_PIG/1-463    143 SPNGTIRNILGGTVFREAII-CKNIPRLVSG-WWKPIIIGRHAYGDQYRATDFVVPGPGKVEISYTPRDGSPKM-- 214
IDH_THET8/1-496      98 SANVTLRKLFETYANVRPVREFPNVPTPYAG-RGIDLVVVRENVEDLYAGIEHMQTPSVAQ--------------- 157
Q9YE81_AERPE/1-432  117 SLNVAIRQALDLYANIRPVRYY-GQPAPHKYADRVDMVIFRENTEDVYAGIEWPHDSPEAARIRRFLAEEF----- 186
IDH_BACSU/1-423     104 SLNVALRQELDLFVCLRPVRYFTGVPSPVKRPEDTDMVIFRENTEDIYAGIEYAKGSEEVQKLISFLQNEL----- 174
IDH_ECOLI/1-416     113 SLNVALRQELDLYICLRPVRYYQGTPSPVKHPELTDMVIFRENSEDIYAGIEWKADSADAEKVIKFLREEM----- 183
Q63WJ4_BURPS/1-419  115 SLNVALRQELDLYVCLRPIQYFKGVPSPVREPEKTNMVIFRENSEDIYAGIEWAAESEQAKKVIKFLQEEM----- 185

Q9X0N2_THEMA/1-399  163 TLLVHKF-EGNGVVMAMHNLEKSIRSFAQSCINYAISEKV-DIWFATKDTISKVYHAYFKDIFQEEVDK-RKEELE 235
IDHP_YEAST/1-428    184 TLKVYDY-KGSGVAMAMYNTDESIEGFAHSSFKLAIDKKL-NLFLSTKNTILKKYDGRFKDIFQEVYEAQYKSKFE 257
IDH1_MYCTU/1-409    169 VHEMVSIPEDGGVVLGMYNFKESIRDFARASFSYGLNAKW-PVYLSTKNTILKAYDGMFKDEFERVYEEEFKAQFE 243
Q21032_CAEEL/1-412  167 QETVFDFK-GPGVSLSMYNTDDSIRDFAHASFKYALQRKF-PLYLSTKNTILKKYDGRFKDIFAEIYP-EYEAEFK 239
IDHC_MOUSE/1-414    166 TYMVHDFEEGGGVAMGMYNQDKSIEDFAHSSFQMALSKGW-PLYLSTKNTILKKYDGRFKDIFQEIYDKKYKSQFE 240
IDHC_HUMAN/1-414    166 TYLVHNFEEGGGVAMGMYNQDKSIEDFAHSSFQMALSKGW-PLYLSTKNTILKKYDGRFKDIFQEIYDKQYKSQFE 240
I3LDC7_PIG/1-463    215 TYMVHDFTDCGGVAMGMYNQDKSIEDFAHSSFQMALSKGW-PLYLSTKNTILKKYDGRFKDIFQEIYDKQYKSQFE 289
IDH_THET8/1-496     158 -------------TLKLISW-KGSEKIVRFAFELARAEGRKKVHCATKSNIMKLAEGTLKRAFEQVAQE-Y----- 213
Q9YE81_AERPE/1-432  187 GI---SIREDAGIGVKPISR-FATRRLMERALEWALRNGNTVVTIMHKGNIMKYTEGAFMRWAYEVALEKFREHVV 258
IDH_BACSU/1-423     175 NVNKIRFPETSGIGIKPVSE-EGTSRLVRAAIDYAIEHGRKSVTLVHKGNIMKFTEGAFKNWGYELAEKEYGDKVF 249
IDH_ECOLI/1-416     184 GVKKIRFPEHCGIGIKPCSE-EGTKRLVRAAIEYAIANDRDSVTLVHKGNIMKFTEGAFKDWGYQLAREEFGGELI 258
Q63WJ4_BURPS/1-419  186 GVKKIRFPQTSGIGIKPVSK-EGTERLVRKAIQYAIDNDRKSVTLVHKGNIMKFTEGAFRDAGYALAQKEFGAELI 260

Q9X0N2_THEMA/1-399  236 KAG--------------------------VNYRYMLIDDAAAQILRSEGGM-LWACMNYEGDIMSDMIASGFGSLG 284
IDHP_YEAST/1-428    258 QLG--------------------------IHYEHRLIDDMVAQMIKSKGGF-IMALKNYDGDVQSDIVAQGFGSLG 306
IDH1_MYCTU/1-409    244 AAG--------------------------LTYEHRLIDDMVAACLKWEGGY-VWACKNYDGDVQSDTVAQGYGSLG 292
Q21032_CAEEL/1-412  240 AAG--------------------------IWYEHRLIDDMVAQAMKSDGGF-VWACKNYDGDVQSDSVAQGYGSLG 288
IDHC_MOUSE/1-414    241 AQK--------------------------ICYEHRLIDDMVAQAMKSEGGF-IWACKNYDGDVQSDSVAQGYGSLG 289
IDHC_HUMAN/1-414    241 AQK--------------------------IWYEHRLIDDMVAQAMKSEGGF-IWACKNYDGDVQSDSVAQGYGSLG 289
I3LDC7_PIG/1-463    290 AQK--------------------------IWYEHRLIDDMVAQAMKSEGGF-IWACKNYDGDVQSDSVAQGYGSLG 338
IDH_THET8/1-496     214 ---------------------------PDIEAVHIIVDNAAHQLVKRPEQFEVIVTTNMNGDILSDLTSGLIGGLG 262
Q9YE81_AERPE/1-432  259 TEQEVQE------------KYGGVRPEGKILVNDRIADNMLQQIITRPWDYQVIVAPNLNGDYISDAASALVGGIG 322
IDH_BACSU/1-423     250 TWAQYDRIAEEQGKDAANKAQSEAEAAGKIIIKDSIADIFLQQILTRPNEFDVVATMNLNGDYISDALAAQVGGIG 325
IDH_ECOLI/1-416     259 DGGPWLKVK-------------NPNTGKEIVIKDVIADAFLQQILLRPAEYDVIACMNLNGDYISDALAAQVGGIG 321
Q63WJ4_BURPS/1-419  261 DGGPWMKFK-------------NPKTGNEIVVKDSIADAFLQQILLRPAEYDVIATLNLNGDYISDALAAQVGGIG 323

Q9X0N2_THEMA/1-399  285 LMTSVLVSPDGV-YEFEAAHGTVRRHYYRYLKGEKTSTNPTASIFAWTGAIRKRGELDGTPEVCEFADKLEKAVIN 359
IDHP_YEAST/1-428    307 LMTSILVTPDGKTFESEAAHGTVTRHYRKYQKGEETSTNSIASIFAWSRGLLKRGELDNTPALCKFANILESATLN 382
IDH1_MYCTU/1-409    293 LMTSVLMTADGKTVEAEAAHGTVTRHYRQYQAGKPTSTNPIASIFAWTRGLQHRGKLDGTPEVIDFAHKLESVVIA 368
Q21032_CAEEL/1-412  289 LMTSVLVCPDGKTVEAEAAHGTVTRHYRMHQKGQETSTNPIASIFAWSRGLAHRATLDKNSALETFANNLEAVCIE 364
IDHC_MOUSE/1-414    290 MMTSVLICPDGKTVEAEAAHGTVTRHYRMYQKGQETSTNPIASIFAWSRGLAHRAKLDNNTELSFFAKALEDVCIE 365
IDHC_HUMAN/1-414    290 MMTSVLVCPDGKTVEAEAAHGTVTRHYRMYQKGQETSTNPIASIFAWTRGLAHRAKLDNNKELAFFANALEEVSIE 365
I3LDC7_PIG/1-463    339 MMTSVLICPDGKTVEAEAAHGTVTRHYRMYQKGQETSTNPIASIFAWTRGLAHRAKLDSNKELSIFANALEEVCIE 414
IDH_THET8/1-496     263 FAPSANIGN--EVAIFEAVHGSAPKYA------GKNVINPTAVLLSAVMML-------RYLEEFATADLIENALLY 323
Q9YE81_AERPE/1-432  323 MAAGMNMGD--GIAVAEPVHGTAPKYA------GKDLINPSAEILSASLLIG------EFMGWREVKSIVEYAIRK 384
IDH_BACSU/1-423     326 IAPGANINYETGHAIFEATHGTAPKYA------GLDKVNPSSVILSGVLLL-------EHLGWNEAADLVIKSMEK 388
IDH_ECOLI/1-416     322 IAPGANIGD--ECALFEATHGTAPKYA------GQDKVNPGSIILSAEMML-------RHMGWTEAADLIVKGMEG 382
Q63WJ4_BURPS/1-419  324 IAPGANLSD--SVAMFEATHGTAPKYA------GKDYVNPGSEILSAEMML-------RHLGWTEAADVIISAMEK 384
```

C continued

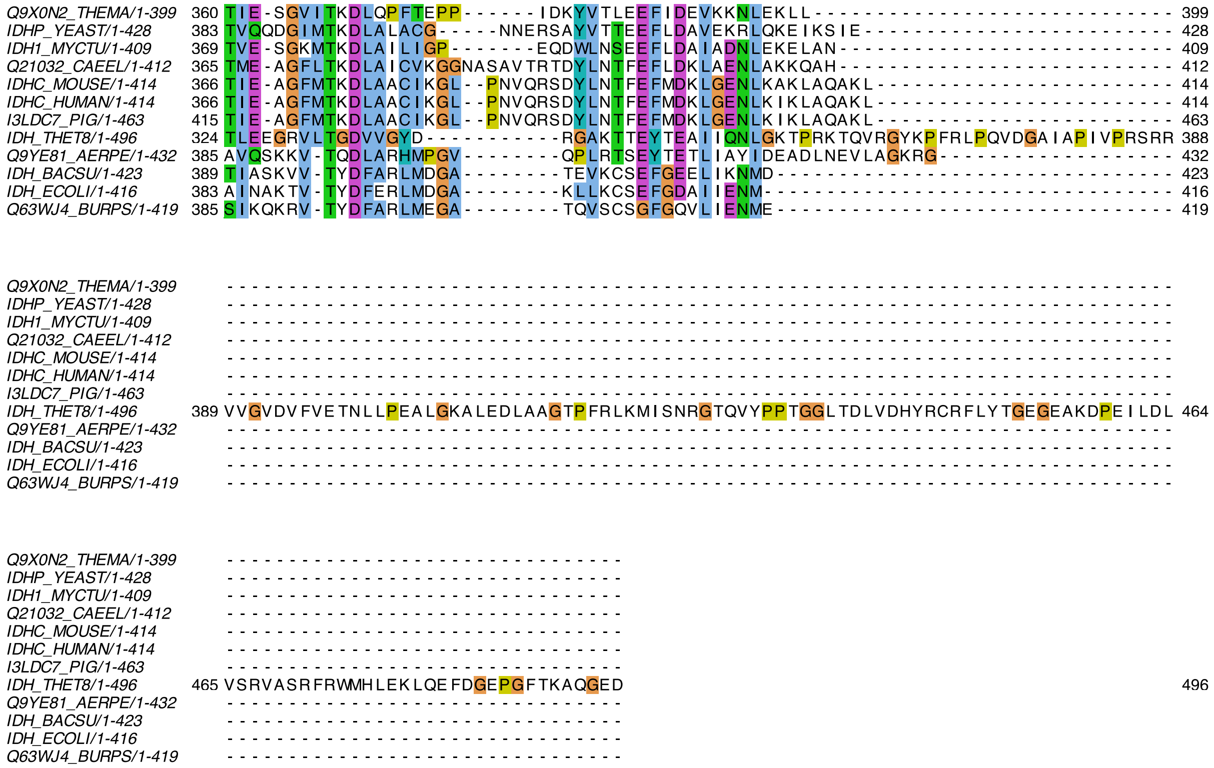

Figure S1. Protein sequence analysis of IDH-1. A. Phylogenetic tree of *C. elegans* IDH-1 (Q21032_CAEEL) is shown with $NADP^+$-dependent IDH enzymes from three IDH subfamilies. Subfamily I consists of dimeric bacterial enzymes (Q9YE81_AERPE, *Aeropyrum pernix*; IDH_BACSU, *Bacillus subtilis*; IDH_ECOLI, *Escherichia coli*; Q63WJ4_BURPS, *Burkholderia pseudomallei*). Subfamily II consists of dimeric eukaryotic enzymes and some dimeric bacterial enzymes (Q9X0N2_THEMA, *Thermotoga maritima*; IDHP_YEAST, *Saccharomyces cerevisiae*; IDH1_MYCTU, *Mycobacterium tuberculosis*; Q21032_CAEEL, *C. elegans*; IDHC_MOUSE, *Mus musculus*; IDHC_HUMAN, *Homo sapiens*; I3LDC7_PIG, *Sus scrofa*). Subfamily IV consists of longer dimeric proteins with larger clasp regions (IDH_THET8, *Thermus thermophilus*). Subfamily III enzymes bind to NAD^+ and are not included. The names in the diagram are the UniProt IDs. B. Percent sequence identity matrix for the IDH sequences shown in A and C. Yellow highlights Subfamily I, and green highlights Subfamily II. The UniProt IDs are shown on the left, and the genus and species abbreviations are shown on the top. C. Sequence alignment of the IDH sequences shown in A and B. The proteins are named using their UniProt IDs. The amino acids are colored using a ClustalW coloring scheme. The sequence alignment and percent identity matrix were determined using Clustal Omega at EMBL-EBI. The phylogenetic tree was determined from the alignment in JalView using neighbor joining with BLOSUM62.
