# Supplementary material for: C. elegans Cytoplasmic Isocitrate Dehydrogenase Neomorphic G98N and R133H Mutants Produce the Oncometabolite 2-Hydroxyglutarate
Source: Int J Mol Sci. 2025 Aug 25;26(17):8238. doi: 10.3390/ijms26178238 (PMC12427979; doi:10.3390/ijms26178238)
Supplement: Supplementary file 1 [file ijms-26-08238-s001.zip › FigureS2.pdf]

Figure S2. IDH-1 structure analysis

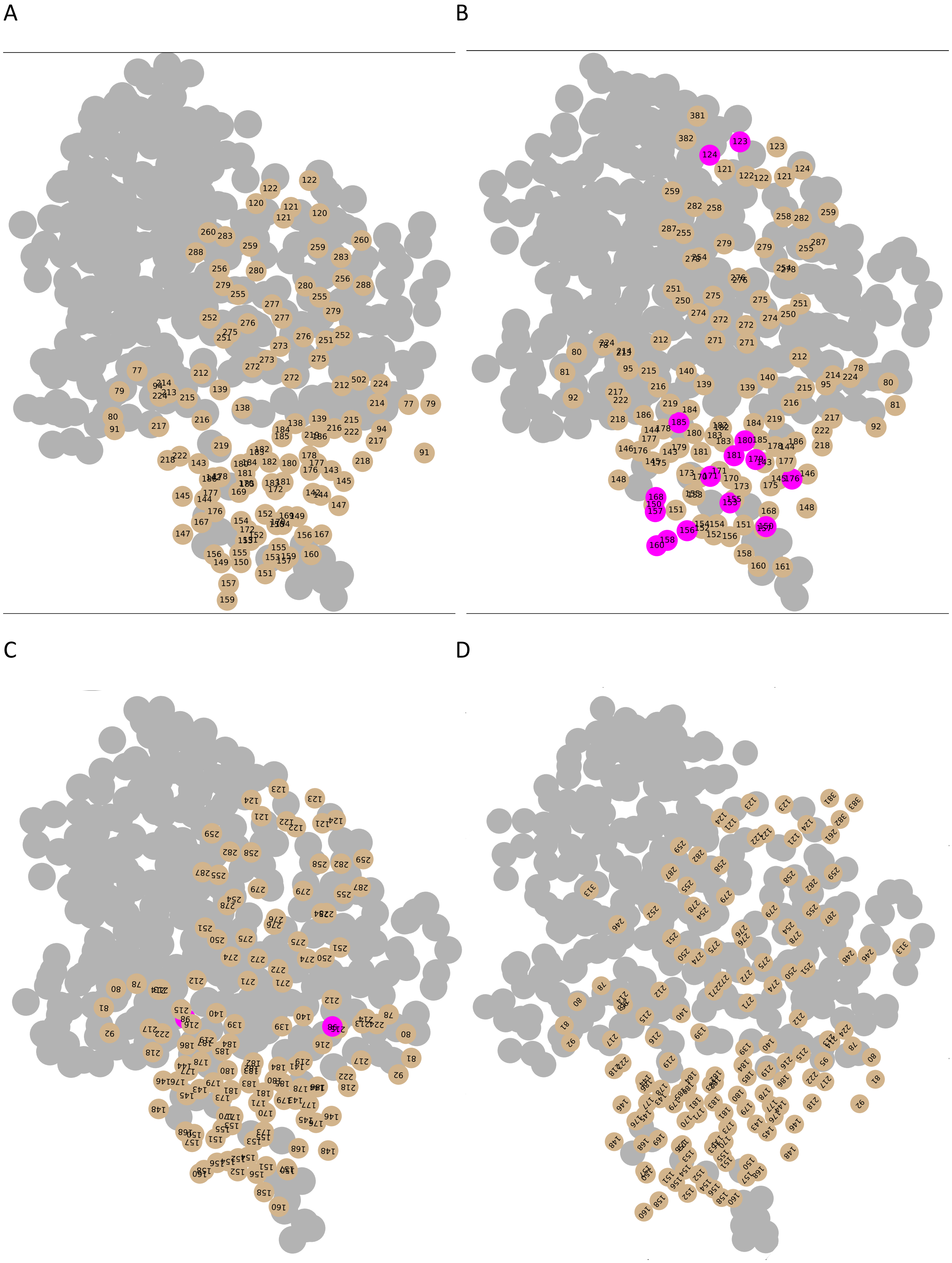

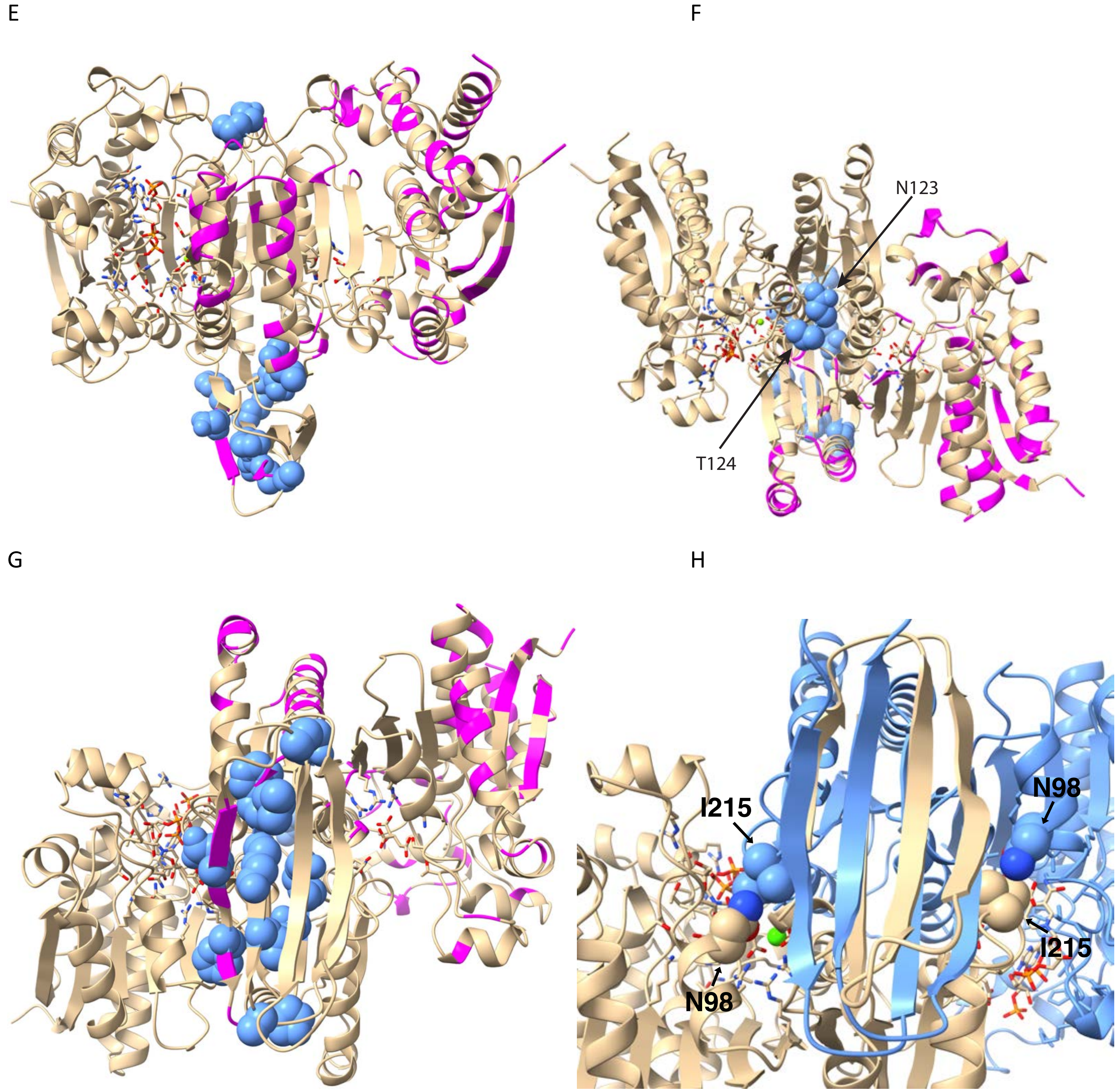
E
F
N123
T124
G
H
I215
N98
N98
I215

I

Human wild-type IDH1, PDB 6BL0								*C. elegans* wild-type IDH-1 homology model							
subunit	donor	AA#	atom	subunit	accept.	AA#	atom	subunit	donor	AA#	atom	subunit	accept.	AA#	atom
/A	SER	122	N	/B	LEU	120	O	/A	ASN	123	N	/B	LEU	121	O
								/A	ASN	123	ND2	/B	ASN	123	OD1
/A	THR	142	OG1	/B	LEU	168	O	/A	THR	143	OG1	/B	THR	169	O
/A	LYS	151	N	/B	THR	157	O	/A	LYS	152	N	/B	VAL	158	O
/A	GLU	153	N	/B	THR	155	O	/A	GLU	154	N	/B	LYS	156	O
/A	THR	155	N	/B	GLU	153	O	/A	LYS	156	N	/B	GLU	154	O
/A	TYR	156	OH	/B	GLY	148	O								
/A	TYR	156	OH	/B	PRO	149	O								
/A	THR	157	N	/B	LYS	151	O	/A	VAL	158	N	/B	LYS	152	O
/A	SER	159	N	/B	PRO	149	O	/A	ALA	160	N	/B	ALA	150	O
/A	SER	159	OG	/B	PRO	149	O								
/A	VAL	178	N	/B	ASN	184	O	/A	VAL	178	N	/B	ASN	184	O
								/A	SER	179	OG	/B	MET	182	O
/A	MET	180	N	/B	MET	182	O	/A	LEU	180	N	/B	MET	182	O
/A	MET	182	N	/B	MET	180	O	/A	MET	182	N	/B	LEU	180	O
/A	ASN	184	N	/B	VAL	178	O	/A	ASN	184	N	/B	VAL	178	O
/A	ASP	186	N	/B	GLY	176	O	/A	ASP	186	N	/B	PRO	176	O
/A	LYS	212	NZ	/B	ASP	275	OD2	/A	LYS	212	NZ	/B	ASP	274	OD2
/A	ASN	213	ND2	/B	ASP	79	OD2	/A	ASN	213	ND2	/B	ASP	80	OD2
/A	LYS	217	NZ	/B	PRO	78	O	/A	LYS	217	NZ	/B	PRO	79	O
/A	LYS	217	NZ	/B	TRP	92	O	/A	LYS	217	NZ	/B	TRP	93	O
/A	LYS	218	N	/B	ASP	143	OD2	/A	LYS	218	N	/B	ASP	144	OD2
/A	TYR	219	N	/B	ASP	143	OD2	/A	TYR	219	N	/B	ASP	144	OD2
/A	TYR	272	OH	/B	ASP	273	OD1	/A	TYR	271	OH	/B	ASP	272	OD1
/A	TYR	272	OH	/B	ASP	273	OD2	/A	TYR	271	OH	/B	ASP	272	OD2
/B	THR	77	OG1	/A	ICT		O3	/B	THR	78	OG1		ICT	5	O3
/B	SER	94	OG	/A	ICT		O4	/B	SER	95	OG		ICT	5	O3
/B	ASN	96	ND2	/A	ICT		O4	/B	ASN	97	ND2		ICT	5	O4
/B	ARG	100	NH1	/A	ICT		O2	/B	ARG	101	NH1		ICT	5	O2
/B	ARG	100	NH1	/A	ICT		O6	/B	ARG	101	NH1		ICT	5	O6
/B	ARG	100	NH2	/A	ICT		O2	/B	ARG	101	NH2		ICT	5	O2
/B	ARG	109	NH1	/A	ICT		O1	/B	ARG	110	NH1		ICT	5	O1
/B	ARG	109	NH2	/A	ICT		O1	/B	ARG	110	NH2		ICT	5	O1
/B	ARG	109	NH2	/A	ICT		O2	/B	ARG	110	NH2		ICT	5	O2
								/B	ASN	123	ND2	/A	ASN	123	OD1
/B	ARG	132	NH1	/A	ICT		O1	/B	ARG	133	NH1		ICT	5	O1
/B	ARG	132	NH1	/A	ICT		O2	/B	ARG	133	NH1		ICT	5	O2
/B	ARG	132	NH1	/A	ICT		O6	/B	ARG	133	NH1		ICT	5	O6
/B	ARG	132	NH2	/A	ICT		O6	/B	ARG	133	NH2		ICT	5	O6
/B	THR	142	OG1	/A	LEU	168	O	/B	THR	143	OG1	/A	THR	169	O
/B	LYS	151	N	/A	THR	157	O	/B	LYS	152	N	/A	VAL	158	O
/B	GLU	153	N	/A	THR	155	O	/B	GLU	154	N	/A	LYS	156	O
/B	THR	155	N	/A	GLU	153	O	/B	LYS	156	N	/A	GLU	154	O
/B	TYR	156	OH	/A	GLY	148	O								
/B	TYR	156	OH	/A	PRO	149	O								
/B	THR	157	N	/A	LYS	151	O	/B	VAL	158	N	/A	LYS	152	O
								/B	SER	159	OG	/A	ALA	150	O
/B	SER	159	N	/A	PRO	149	O	/B	ALA	160	N	/A	ALA	150	O
/B	VAL	178	N	/A	ASN	184	O	/B	VAL	178	N	/A	ASN	184	O
								/B	SER	179	OG	/A	MET	182	O
/B	MET	180	N	/A	MET	182	O	/B	LEU	180	N	/A	MET	182	O
/B	MET	182	N	/A	MET	180	O	/B	MET	182	N	/A	LEU	180	O
/B	ASN	184	N	/A	VAL	178	O	/B	ASN	184	N	/A	VAL	178	O

I cont.

**Human wild-type IDH1, PDB 6BL0**								***C. elegans* wild-type IDH-1 homology model**							
subunit	donor	AA#	atom	subunit	accept.	AA#	atom	subunit	donor	AA#	atom	subunit	accept.	AA#	atom
/B	ASP	186	N	/A	GLY	176	O	/B	ASP	186	N	/A	PRO	176	O
/B	LYS	212	NZ	/A	ASP	275	OD2	/B	LYS	212	NZ	/A	ASP	274	OD2
/B	LYS	212	NZ	/A	ICT		O6	/B	LYS	212	NZ		ICT	2	O6
/B	LYS	217	NZ	/A	PRO	78	O	/B	LYS	217	NZ	/A	PRO	79	O
/B	LYS	218	N	/A	ASP	143	OD2	/B	LYS	218	N	/A	ASP	144	OD2
/B	TYR	219	N	/A	ASP	143	OD2	/B	TYR	219	N	/A	ASP	144	OD2
/B	TYR	272	OH	/A	ASP	273	OD1	/B	TYR	271	OH	/A	ASP	272	OD1
/B	TYR	272	OH	/A	ASP	273	OD2	/B	TYR	271	OH	/A	ASP	272	OD2

Figure S2. IDH-1 structure analysis. A-D. These are interface residue plots generated by ChimeraX. They are a 2D representation of the amino acids involved in the 3D interactions between the two subunits in each protein structure. The structures shown are: A. human IDH1 from PDB 6BL0, B. homology model of wild-type *C. elegans* IDH-1, C. homology model of IDH-1 G98N, and D. homology model of IDH-1 R133H. The plots in A and B are in the same orientation because the homology model of wild-type IDH-1 was built using the human structure shown in A. The other two models were generated using different template structures (see methods), so those plots were rotated to put them in a similar orientation to A and B. In B, the amino acids that are changed in *C. elegans* IDH-1 compared to human IDH1 are colored magenta. The changes are in two areas – the very top of the protein and in the clasp region on the bottom. The clasp region consists of beta sheets with the main interactions along the backbone (see hydrogen bond list in I). In C, the mutated amino acid G98N is colored magenta. It makes a close contact with I215 in the other subunit (see H). The R133 amino acid is pointed into the active site, and it is not involved in intersubunit interactions. E. Homology model of C. elegans IDH-1 in the same orientation as in the figures in the manuscript. In the right subunit, the locations of the changed amino acids between human IDH1 and *C. elegans* IDH-1 that are not involved in intersubunit interactions are colored magenta. The changed amino acids in the right-hand subunit that are involved in intersubunit interactions (and shown in magenta in B) are shown in blue in space-filling view. F. The structure in E is rotated forward to show the amino acids at the top of the structure. G. The structure in E is rotated backward to show the amino acids in the clasp at the bottom of the structure. H. The structure of the *C. elegans* G98N homolog model with one subunit in blue and one in beige is shown in a similar orientation as in G. The clasp region is the beta-sheet structure in the middle. Amino acids N98 and I215 are in spacefilling view and labeled. The nitrogen in N98 is closest to I215, and it is dark blue. I. Hydrogen bonds in human IDH1 from PDB 6BL0 are listed on the left and hydrogen bonds in the homology model of wild-type *C. elegans* IDH-1 are on the right. The hydrogen bonds involving the same amino acids in each subunit are highlighted in yellow. The hydrogen bonds involving amino acids in the same position of the sequence alignment are shown in blue. The hydrogen bonds that are only found in one structure or the other are not highlighted. The columns show the amino acid and amino acid number (AA #) for the donor atom first and the amino acid and AA# for the acceptor (accept.) atom second.
