# Supplementary material for: C. elegans Cytoplasmic Isocitrate Dehydrogenase Neomorphic G98N and R133H Mutants Produce the Oncometabolite 2-Hydroxyglutarate
Source: Int J Mol Sci. 2025 Aug 25;26(17):8238. doi: 10.3390/ijms26178238 (PMC12427979; doi:10.3390/ijms26178238)
Supplement: Supplementary file 1 [file ijms-26-08238-s001.zip › FigureS3.pdf]

A

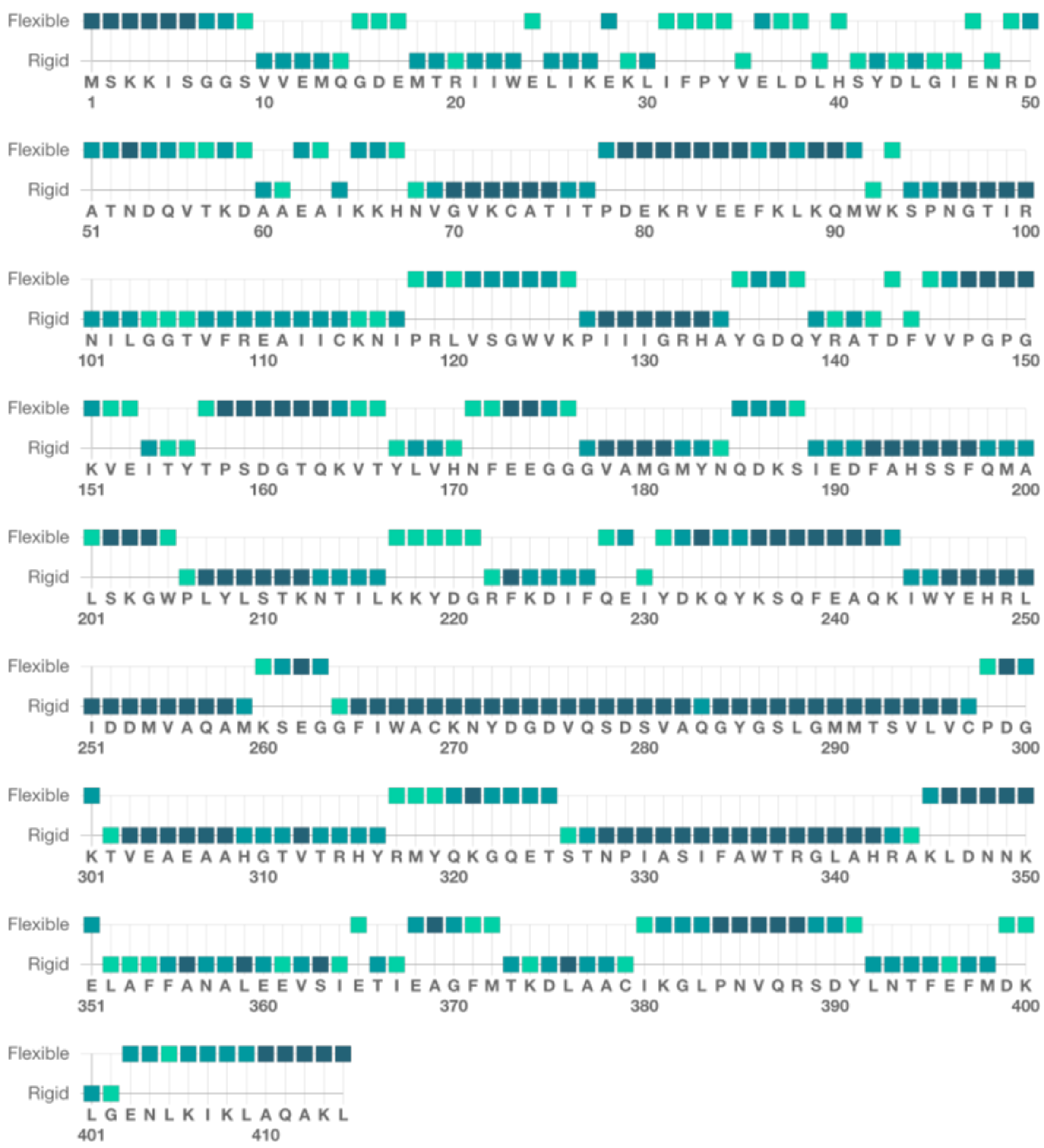
Flexible
Rigid
M S K K I S G G S V V E M Q G D E M T R I I W E L I K E K L I F P Y V E L D L H S Y D L G I E N R D
1 10 20 30 40 50
A T N D Q V T K D A A E A I K K H N V G V K C A T I T P D E K R V E E F K L K Q M W K S P N G T I R
51 60 70 80 90 100
N I L G G T V F R E A I I C K N I P R L V S G W V K P I I I G R H A Y G D Q Y R A T D F V V P G P G
101 110 120 130 140 150
K V E I T Y T P S D G T Q K V T Y L V H N F E E G G G V A M G M Y N Q D K S I E D F A H S S F Q M A
151 160 170 180 190 200
L S K G W P L Y L S T K N T I L K K Y D G R F K D I F Q E I Y D K Q Y K S Q F E A Q K I W Y E H R L
201 210 220 230 240 250
I D D M V A Q A M K S E G G F I W A C K N Y D G D V Q S D S V A Q G Y G S L G M M T S V L V C P D G
251 260 270 280 290 300
K T V E A E A A H G T V T R H Y R M Y Q K G Q E T S T N P I A S I F A W T R G L A H R A K L D N N K
301 310 320 330 340 350
E L A F F A N A L E E V S I E T I E A G F M T K D L A A C I K G L P N V Q R S D Y L N T F E F M D K
351 360 370 380 390 400
L G E N L K I K L A Q A K L
401 410

B

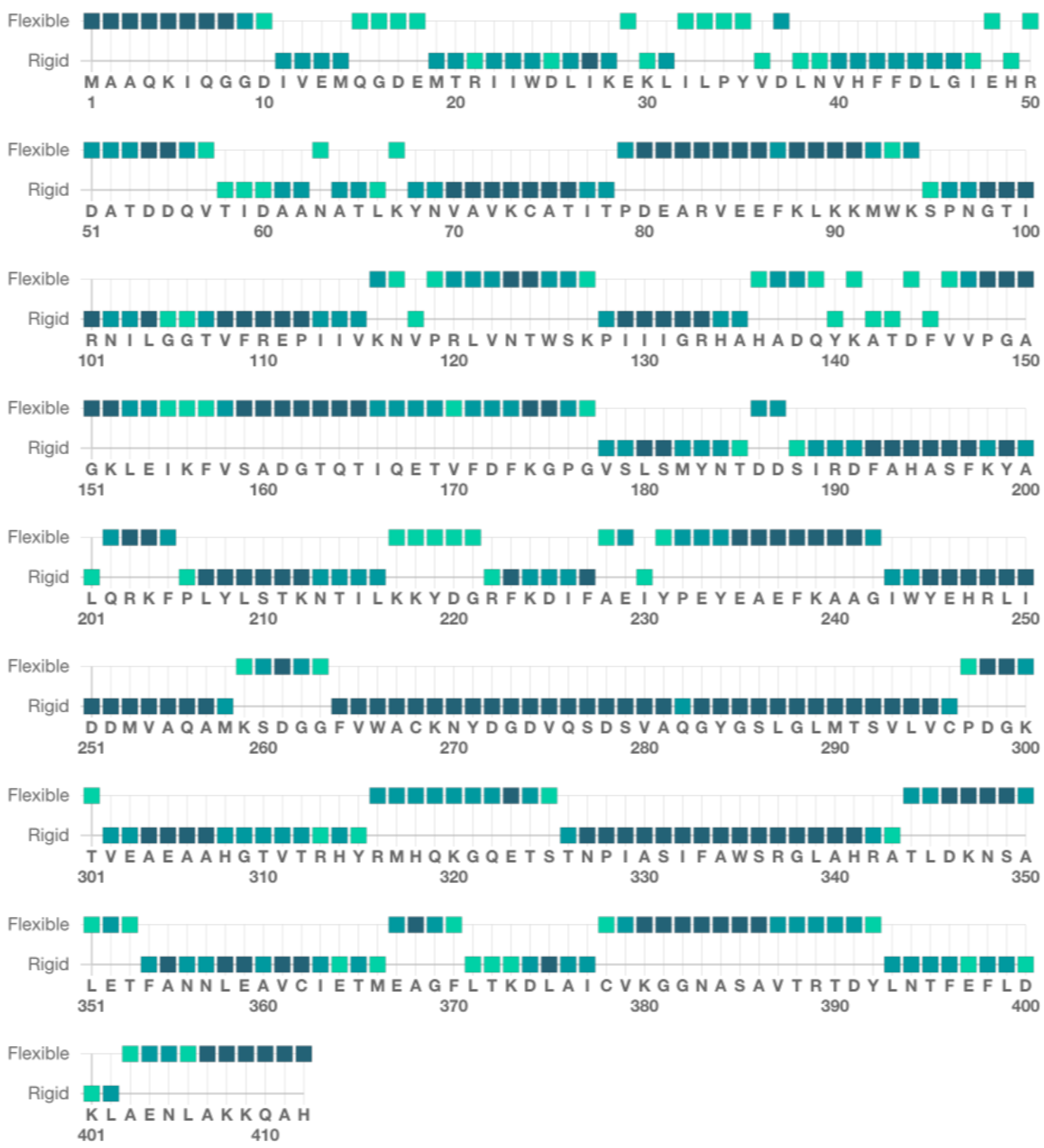

Supplemental Figure S3. MEDUSA analysis of IDH1 and IDH-1. The amino acid sequences of (A) human IDH1 and (B) *C. elegans* IDH-1 were submitted to the Medusa server (https://www.dsimb.inserm.fr/MEDUSA/), and the predicted flexibility is shown using a strict two-point scale.
