# Supplementary material for: C. elegans Cytoplasmic Isocitrate Dehydrogenase Neomorphic G98N and R133H Mutants Produce the Oncometabolite 2-Hydroxyglutarate
Source: Int J Mol Sci. 2025 Aug 25;26(17):8238. doi: 10.3390/ijms26178238 (PMC12427979; doi:10.3390/ijms26178238)
Supplement: Supplementary file 1 [file ijms-26-08238-s001.zip › FigureS4.pdf]

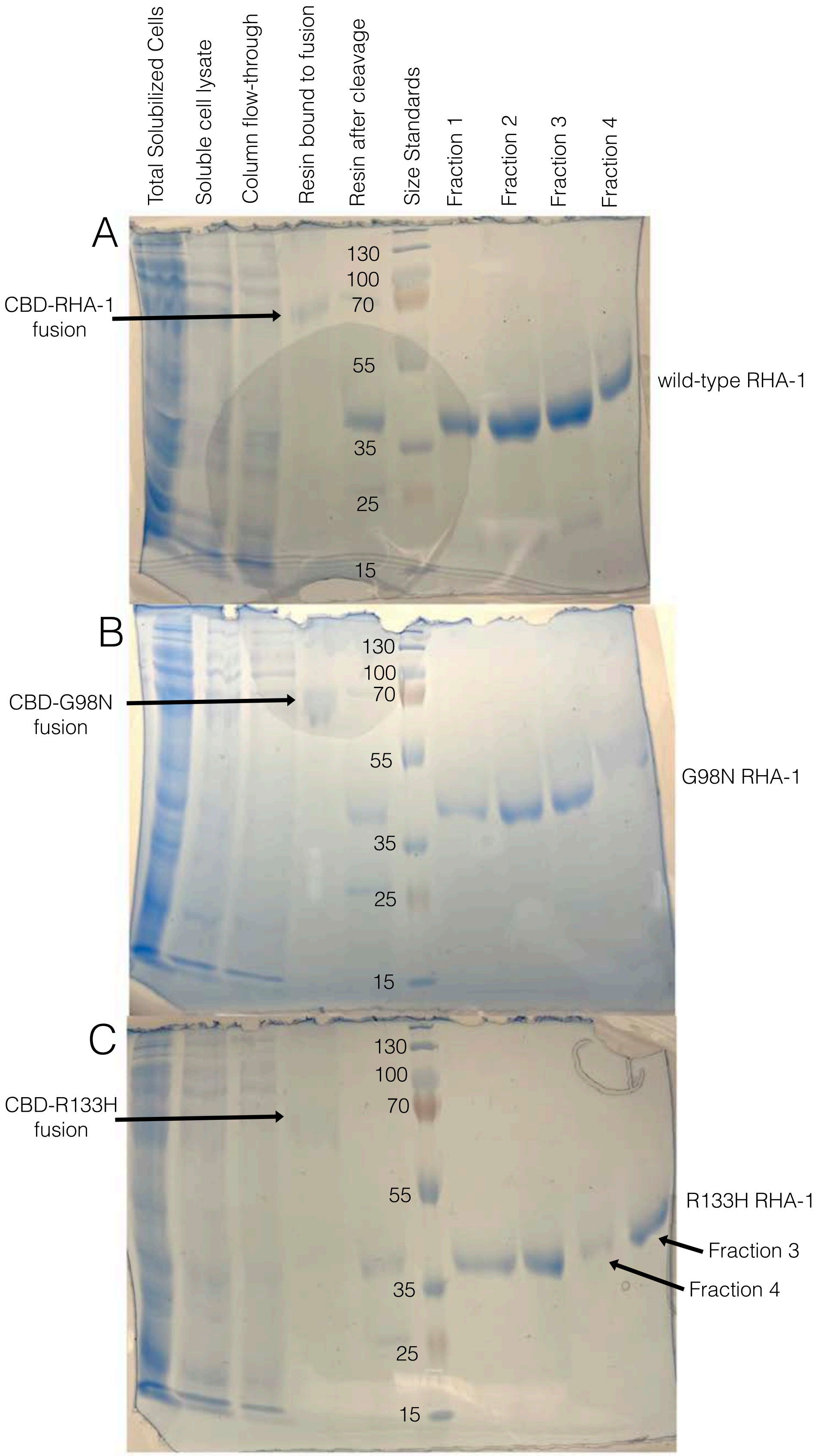

Total Solubilized Cells
Soluble cell lysate
Column flow-through
Resin bound to fusion
Resin after cleavage
Size Standards
Fraction 1
Fraction 2
Fraction 3
Fraction 4
A
CBD-RHA-1 fusion
130
100
70
55
35
25
15
wild-type RHA-1
B
CBD-G98N fusion
130
100
70
55
35
25
15
G98N RHA-1
C
CBD-R133H fusion
130
100
70
55
35
25
15
R133H RHA-1
Fraction 3
Fraction 4

Supplemental Figure S4. SDS-PAGE gels of purified RHA-1 proteins. The 10% Laemmli SDS-PAGE gels stained with Coomassie blue show (A) wild-type RHA-1, (B) G97N mutant, and (C) R133H mutant. All gels are loaded in the same order except that the last two fractions are reversed in (C) as labeled. The soluble cell lysates containing the proteins fused to a chitin binding domain (CBD) were loaded onto a chitin resin as described in the methods. The bound protein (73.8 kD) is shown in lane 4 with an arrow. Then DTT was added to cleave off the CBD, and the resin after cleavage is shown in lane 5. The purified proteins (45.9 kD) are shown in the four lanes on the right. For each protein, fractions 1-3 were combined for dialysis. The size standards are labeled in kD. These are the original photos of the gels, and the contrast of each image was adjusted slightly in the entire image using “levels” in Photoshop version 26.8.1.
