# Supplementary material for: C. elegans Cytoplasmic Isocitrate Dehydrogenase Neomorphic G98N and R133H Mutants Produce the Oncometabolite 2-Hydroxyglutarate
Source: Int J Mol Sci. 2025 Aug 25;26(17):8238. doi: 10.3390/ijms26178238 (PMC12427979; doi:10.3390/ijms26178238)

Quantitative Analysis Summary Report

**Batch Results**	D:\MassHunter\Data\Users\062023\Walstrom\LCMS6_13\QuantResults\ICT_6_13analysis_batch.batch.bin		
**Analysis Time**	6/14/2023 11:03 AM	**Analyst Name**	admin
**Report Time**	6/14/2023 11:04 AM	**Reporter Name**	admin
**Last Calib Update**	6/14/2023 11:03 AM	**Batch State**	Processed
**Quant Batch Version**	B.05.01	**Quant Report Version**	B.05.01

Sequence Table

Data File	Sample Name	Sample Type	Position	Inj. Volume	Level	Acq Method File
ICT_0_1uM.d	ICT_0_1	Calibration	P1-F1	Per method	1	NCF_LCMS_neg_IDH.m
ICT_0_5uM.d	ICT_0_5	Calibration	P1-F2	Per method	2	NCF_LCMS_neg_IDH.m
ICT_1uM.d	ICT_1	Calibration	P1-E1	Per method	3	NCF_LCMS_neg_IDH.m
ICT_2uM.d	ICT_2	Calibration	P1-E2	Per method	4	NCF_LCMS_neg_IDH.m
ICT_5uM.d	ICT_5	Calibration	P1-E3	Per method	5	NCF_LCMS_neg_IDH_Vial_Wash2.m
ICT_12uM.d	ICT_12	Calibration	P1-E4	Per method	6	NCF_LCMS_neg_IDH.m
ICT_25uM.d	ICT_25	Calibration	P1-E5	Per method	7	NCF_LCMS_neg_IDH.m
blank13_50mM_no_wash.d	blank_50mM_13	Blank	P1-A3	Per method		NCF_LCMS_neg_IDH.m
blank15_50mM_no_wash.d	blank_50mM_15	Blank	P1-A7	Per method		NCF_LCMS_neg_IDH.m
blank17_50mM_no_wash.d	blank_50mM_17	Blank	P1-B3	Per method		NCF_LCMS_neg_IDH.m
blank18_50mM_no_wash.d	blank_50mM_18	Blank	P1-B7	Per method		NCF_LCMS_neg_IDH.m
WT_1a.d	WT_1a	Sample	P1-A7	Per method		NCF_LCMS_neg_IDH.m
G98_1a.d	G98_1a	Sample	P1-A8	Per method		NCF_LCMS_neg_IDH.m
R133_1.d	R133_1	Sample	P1-A9	Per method		NCF_LCMS_neg_IDH.m
G98_1a_slow.d	G98_1a	Sample	P1-A8	Per method		NCF_LCMS_neg_IDH.m
WT_1a_2nd_run.d	WT_1a_2	Sample	P1-A1	Per method		NCF_LCMS_neg_IDH.m
WT_1b.d	WT_1b	Sample	P1-A4	Per method		NCF_LCMS_neg_IDH.m
WT_2.d	WT_2	Sample	P1-A5	Per method		NCF_LCMS_neg_IDH.m
G98_1b.d	G98_1b	Sample	P1-A8	Per method		NCF_LCMS_neg_IDH.m
G98_2.d	G98_2	Sample	P1-B1	Per method		NCF_LCMS_neg_IDH.m
R133_1_2nd_run.d	R133_1	Sample	P1-B4	Per method		NCF_LCMS_neg_IDH.m
R133_2.d	R133_2	Sample	P1-B5	Per method		NCF_LCMS_neg_IDH.m
R133_3.d	R133_3	Sample	P1-B6	Per method		NCF_LCMS_neg_IDH.m
rxnmix_div_2.d	rxn_mix_div_2	Sample	P1-B8	Per method		NCF_LCMS_neg_IDH.m

Quantitation Results

Target Compound *isocitrate*

Data File	Compound	ISTD	Sample Type	Response	ISTD Resp	Resp Ratio	Conc.	Exp Conc	Accuracy
ICT_0_1uM.d	isocitrate		Calibration	125			0.24	0.10	239.03
ICT_0_5uM.d	isocitrate		Calibration	663			0.74	0.50	147.40
ICT_1uM.d	isocitrate		Calibration	948			1.00	1.00	100.13
ICT_2uM.d	isocitrate		Calibration	2015			1.99	2.00	99.55

Quantitative Analysis Summary Report

Data File	Compound	ISTD	Sample Type	Response	ISTD Resp	Resp Ratio	Conc.	Exp Conc	Accuracy
ICT_5uM.d	isocitrate		Calibration	5089			4.84	5.00	96.80
ICT_12uM.d	isocitrate		Calibration	12324			11.55	12.00	96.22
ICT_25uM.d	isocitrate		Calibration	27102			25.24	25.00	100.98
blank13_50mM_no_wash.d	isocitrate		Blank				0.00		
blank15_50mM_no_wash.d	isocitrate		Blank				0.00		
blank17_50mM_no_wash.d	isocitrate		Blank	17			0.14		
blank18_50mM_no_wash.d	isocitrate		Blank	22			0.14		
WT_1a.d	isocitrate		Sample	1236			1.27		
G98_1a.d	isocitrate		Sample	786			0.85		
R133_1.d	isocitrate		Sample	765			0.83		
G98_1a_slow.d	isocitrate		Sample	1134			1.17		
WT_1a_2nd_run.d	isocitrate		Sample	1456			1.47		
WT_1b.d	isocitrate		Sample	2041			2.01		
WT_2.d	isocitrate		Sample	1949			1.93		
G98_1b.d	isocitrate		Sample	743			0.81		
G98_2.d	isocitrate		Sample	814			0.88		
R133_1_2nd_run.d	isocitrate		Sample	568			0.65		
R133_2.d	isocitrate		Sample	908			0.96		
R133_3.d	isocitrate		Sample	808			0.87		
rxnmix_div_2.d	isocitrate		Sample	733			0.80		

Quantitative Analysis Calibration Report

**Batch Results**	D:\MassHunter\Data\Users\062023\Walstrom\LCMS6_13\QuantResults\ICT_6_13analysis_batch.batcl		
**Analysis Time**	6/14/2023 11:03 AM	**Analyst Name**	admin
**Report Time**	6/14/2023 11:04 AM	**Reporter Name**	admin
**Last Calib Update**	6/14/2023 11:03 AM	**Batch State**	Processed

Calibration Info

Target Compound *isocitrate*

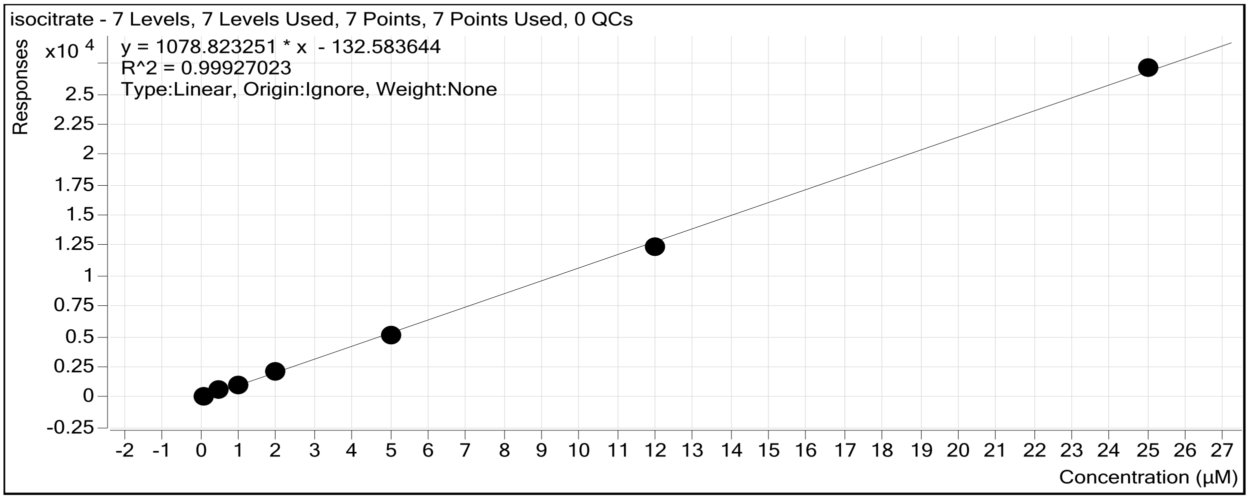

Calibration STD	Cal Type	Level	Enabled	Response	Exp Conc	RF
D:\MassHunter\Data\Users\062023\Walstrom\LCMS6_13\ICT_0_1uM.d	Calibration	1	☑	125	0.1000	1252.8787
D:\MassHunter\Data\Users\062023\Walstrom\LCMS6_13\ICT_0_5uM.d	Calibration	2	☑	663	0.5000	1325.0538
D:\MassHunter\Data\Users\062023\Walstrom\LCMS6_13\ICT_1uM.d	Calibration	3	☑	948	1.0000	947.6246
D:\MassHunter\Data\Users\062023\Walstrom\LCMS6_13\ICT_2uM.d	Calibration	4	☑	2015	2.0000	1007.6395
D:\MassHunter\Data\Users\062023\Walstrom\LCMS6_13\ICT_5uM.d	Calibration	5	☑	5089	5.0000	1017.8254
D:\MassHunter\Data\Users\062023\Walstrom\LCMS6_13\ICT_12uM.d	Calibration	6	☑	12324	12.0000	1027.0089
D:\MassHunter\Data\Users\062023\Walstrom\LCMS6_13\ICT_25uM.d	Calibration	7	☑	27102	25.0000	1084.0921

Quantitative Analysis Sample Report

**Batch Results**	D:\MassHunter\Data\Users\062023\Walstrom\LCMS6_13\QuantResults\ICT_6_13analysis_batch.batch.bin		
**Analysis Time**	6/14/2023 11:03 AM	**Analyst Name**	admin
**Report Time**	6/14/2023 11:04 AM	**Reporter Name**	admin
**Last Calib Update**	6/14/2023 11:03 AM	**Batch State**	Processed

Analysis Info

**Acq Time**	2023-06-13 23:01	**Data File**	ICT_0_1uM.d
**Position**	P1-F1	**Sample Name**	ICT_0_1
**Dilution**	1	**Sample Info**	
**Inj. Volume**	Per method	**Acq Method File**	NCF_LCMS_neg_IDH.m
**Sample Type**	Calibration	**Comment**	

Sample Chromatogram

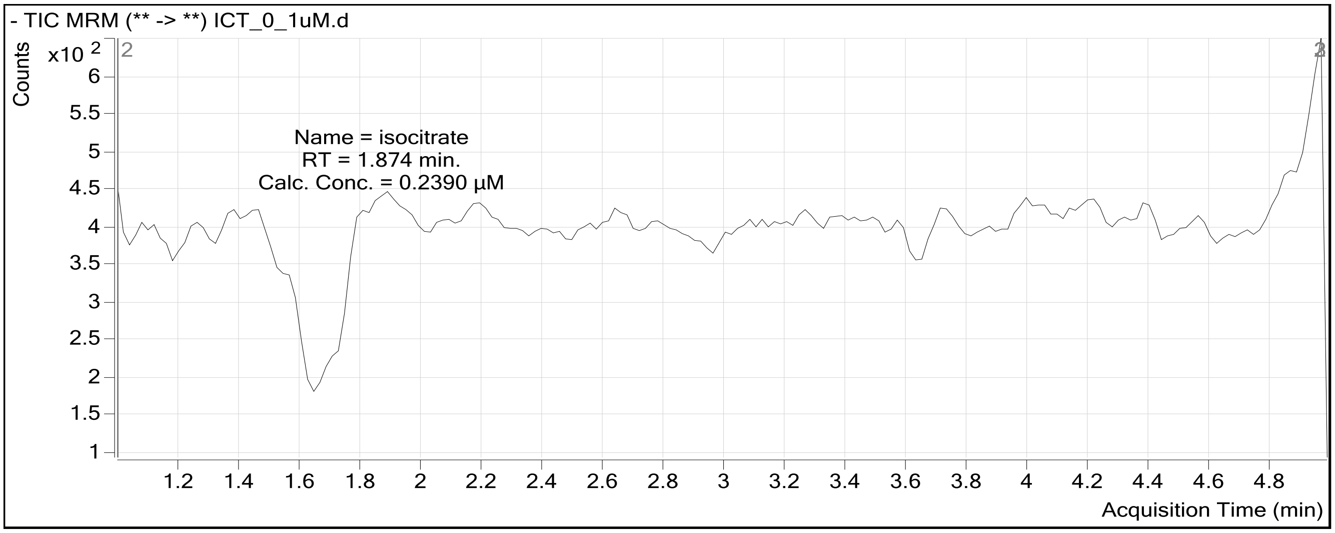

Quantitation Results

Compound	ISTD	RT	Response	ISTD Resp	RR	Conc.	Accuracy
isocitrate		1.874	125			0.24	239.03

Compound Graphics

Target Compound *isocitrate*

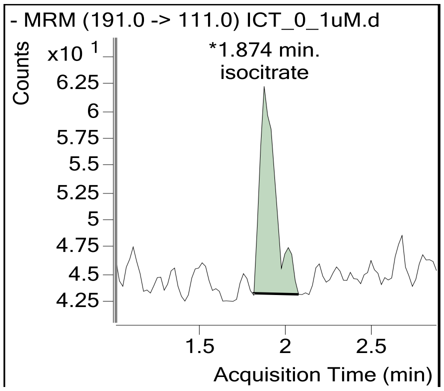

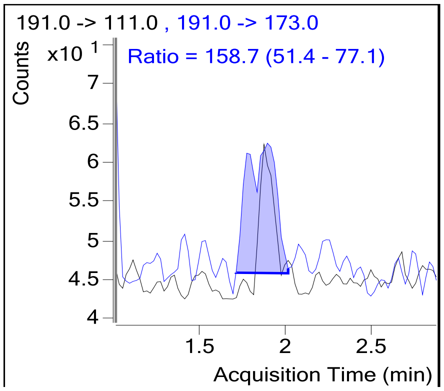

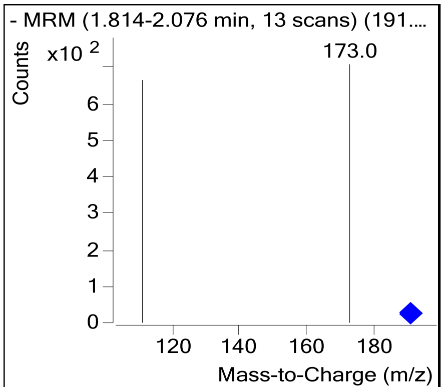

Quantitative Analysis Sample Report

**Batch Results**	D:\MassHunter\Data\Users\062023\Walstrom\LCMS6_13\QuantResults\ICT_6_13analysis_batch.batch.bin		
**Analysis Time**	6/14/2023 11:03 AM	**Analyst Name**	admin
**Report Time**	6/14/2023 11:04 AM	**Reporter Name**	admin
**Last Calib Update**	6/14/2023 11:03 AM	**Batch State**	Processed

Analysis Info

**Acq Time**	2023-06-13 23:12	**Data File**	ICT_0_5uM.d
**Position**	P1-F2	**Sample Name**	ICT_0_5
**Dilution**	1	**Sample Info**	
**Inj. Volume**	Per method	**Acq Method File**	NCF_LCMS_neg_IDH.m
**Sample Type**	Calibration	**Comment**	

Sample Chromatogram

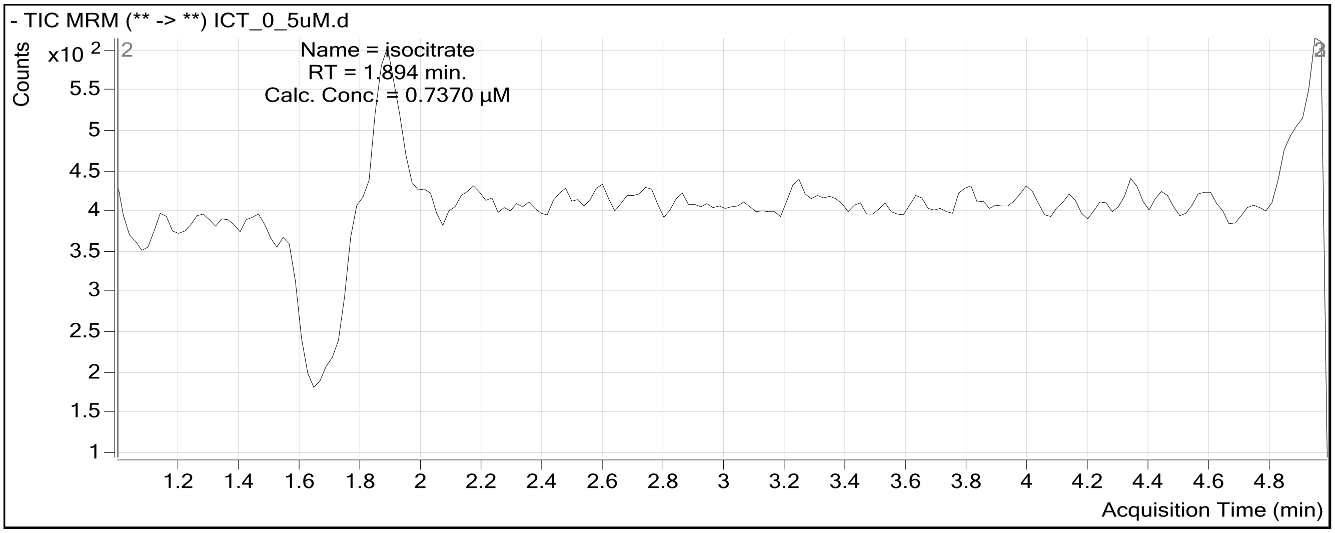

Quantitation Results

Compound	ISTD	RT	Response	ISTD Resp	RR	Conc.	Accuracy
isocitrate		1.894	663			0.74	147.40

Compound Graphics

Target Compound *isocitrate*

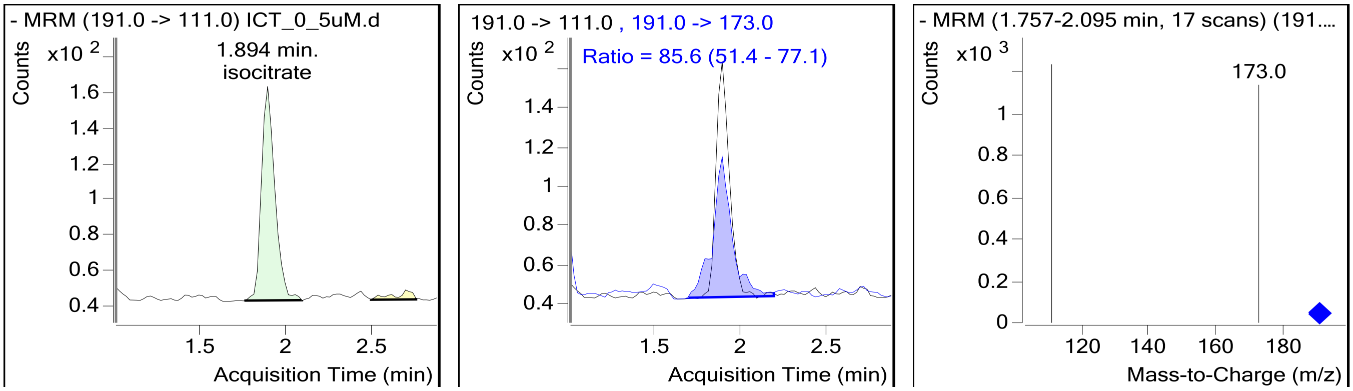

Quantitative Analysis Sample Report

**Batch Results**	D:\MassHunter\Data\Users\062023\Walstrom\LCMS6_13\QuantResults\ICT_6_13analysis_batch.batch.bin		
**Analysis Time**	6/14/2023 11:03 AM	**Analyst Name**	admin
**Report Time**	6/14/2023 11:04 AM	**Reporter Name**	admin
**Last Calib Update**	6/14/2023 11:03 AM	**Batch State**	Processed

Analysis Info

**Acq Time**	2023-06-13 23:23	**Data File**	ICT_1uM.d
**Position**	P1-E1	**Sample Name**	ICT_1
**Dilution**	1	**Sample Info**	
**Inj. Volume**	Per method	**Acq Method File**	NCF_LCMS_neg_IDH.m
**Sample Type**	Calibration	**Comment**	

Sample Chromatogram

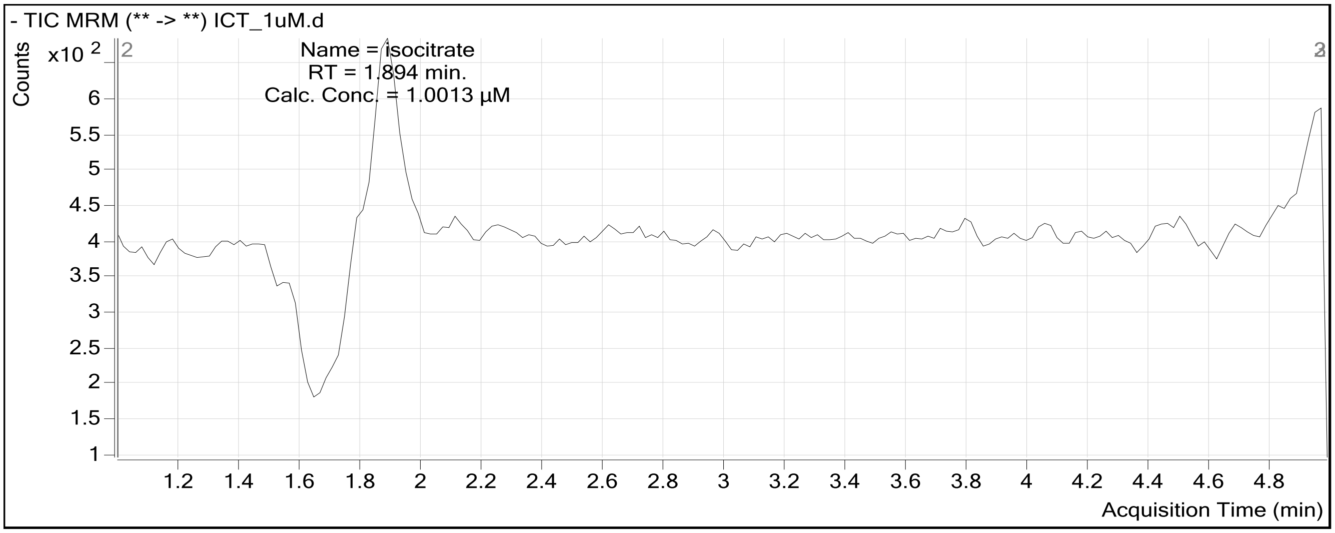

Quantitation Results

Compound	ISTD	RT	Response	ISTD Resp	RR	Conc.	Accuracy
isocitrate		1.894	948			1.00	100.13

Compound Graphics

Target Compound *isocitrate*

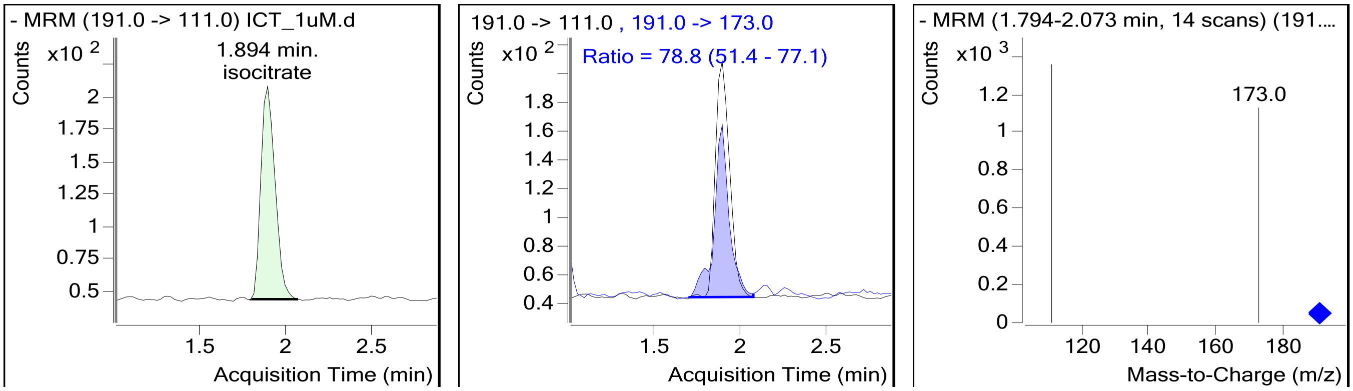

Quantitative Analysis Sample Report

**Batch Results**	D:\MassHunter\Data\Users\062023\Walstrom\LCMS6_13\QuantResults\ICT_6_13analysis_batch.batch.bin		
**Analysis Time**	6/14/2023 11:03 AM	**Analyst Name**	admin
**Report Time**	6/14/2023 11:04 AM	**Reporter Name**	admin
**Last Calib Update**	6/14/2023 11:03 AM	**Batch State**	Processed

Analysis Info

**Acq Time**	2023-06-13 23:34	**Data File**	ICT_2uM.d
**Position**	P1-E2	**Sample Name**	ICT_2
**Dilution**	1	**Sample Info**	
**Inj. Volume**	Per method	**Acq Method File**	NCF_LCMS_neg_IDH.m
**Sample Type**	Calibration	**Comment**	

Sample Chromatogram

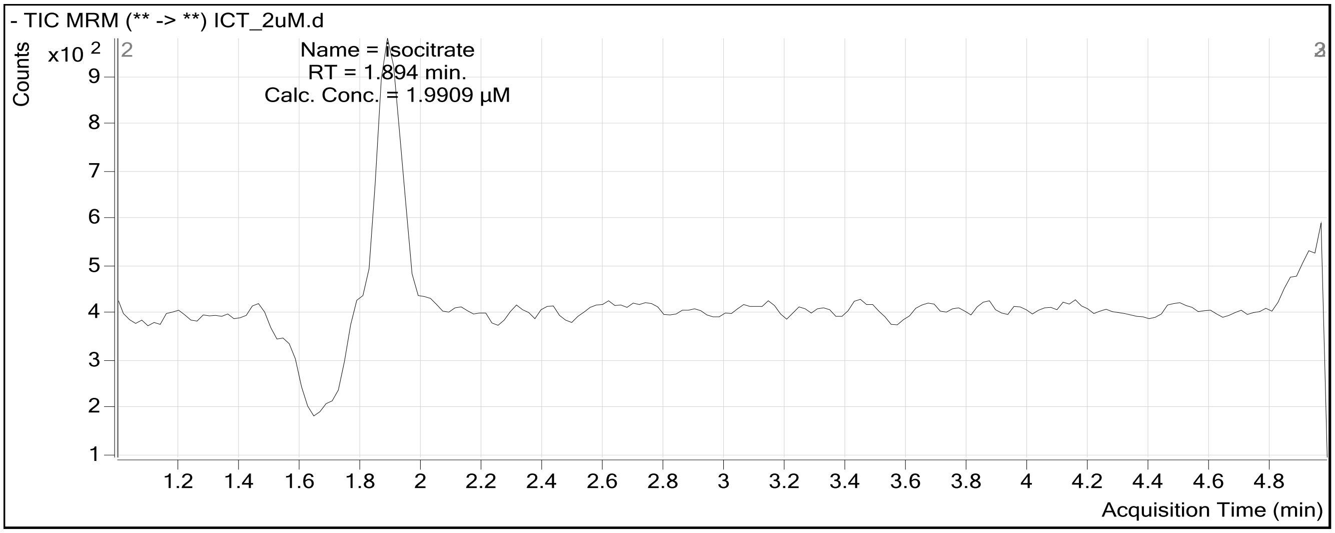

Quantitation Results

Compound	ISTD	RT	Response	ISTD Resp	RR	Conc.	Accuracy
isocitrate		1.894	2015			1.99	99.55

Compound Graphics

Target Compound *isocitrate*

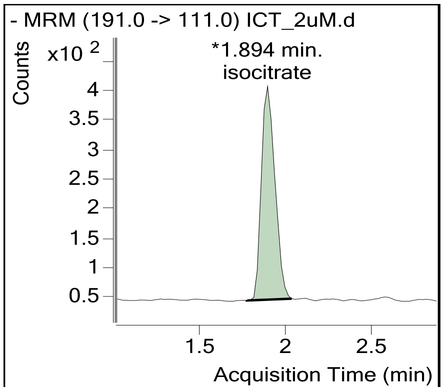

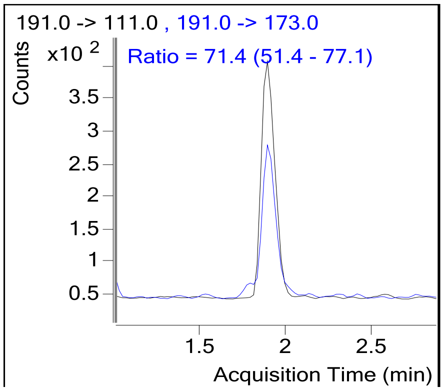

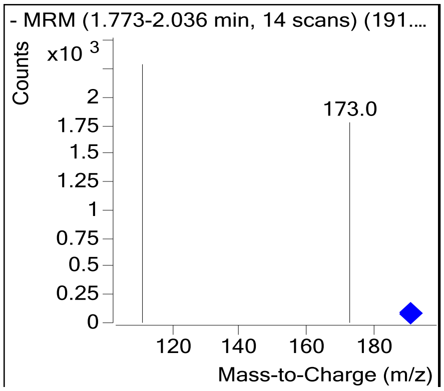

Quantitative Analysis Sample Report

**Batch Results**	D:\MassHunter\Data\Users\062023\Walstrom\LCMS6_13\QuantResults\ICT_6_13analysis_batch.batch.bin		
**Analysis Time**	6/14/2023 11:03 AM	**Analyst Name**	admin
**Report Time**	6/14/2023 11:04 AM	**Reporter Name**	admin
**Last Calib Update**	6/14/2023 11:03 AM	**Batch State**	Processed

Analysis Info

**Acq Time**	2023-06-13 23:45	**Data File**	ICT_5uM.d
**Position**	P1-E3	**Sample Name**	ICT_5
**Dilution**	1	**Sample Info**	
**Inj. Volume**	Per method	**Acq Method File**	NCF_LCMS_neg_IDH_Vial_Wash2.m
**Sample Type**	Calibration	**Comment**	

Sample Chromatogram

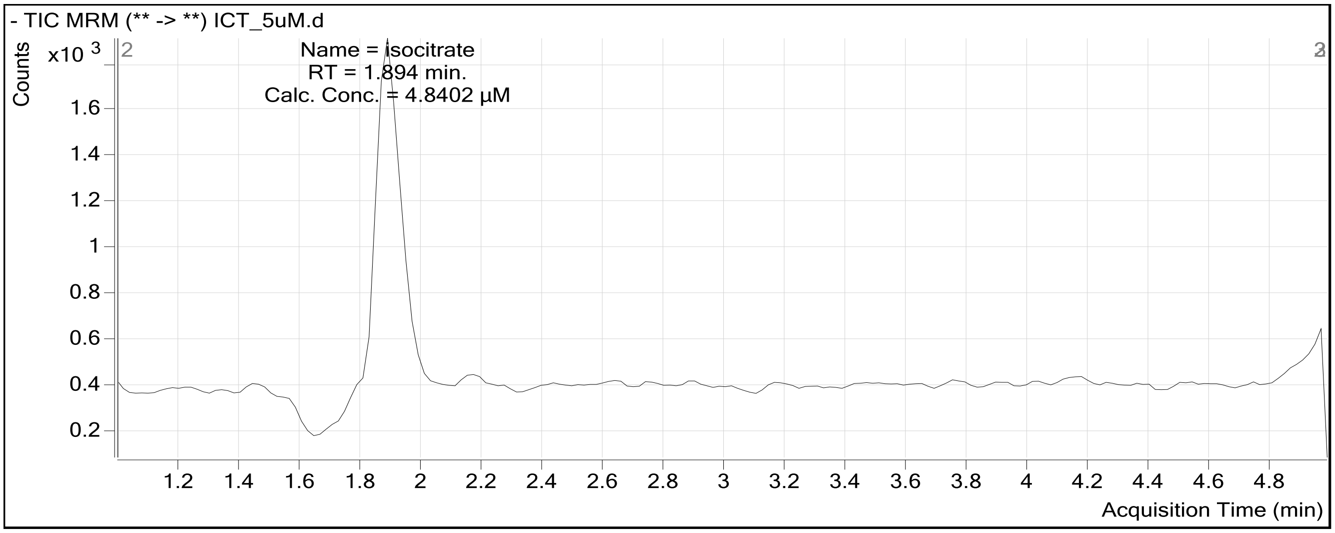

Quantitation Results

Compound	ISTD	RT	Response	ISTD Resp	RR	Conc.	Accuracy
isocitrate		1.894	5089			4.84	96.80

Compound Graphics

Target Compound *isocitrate*

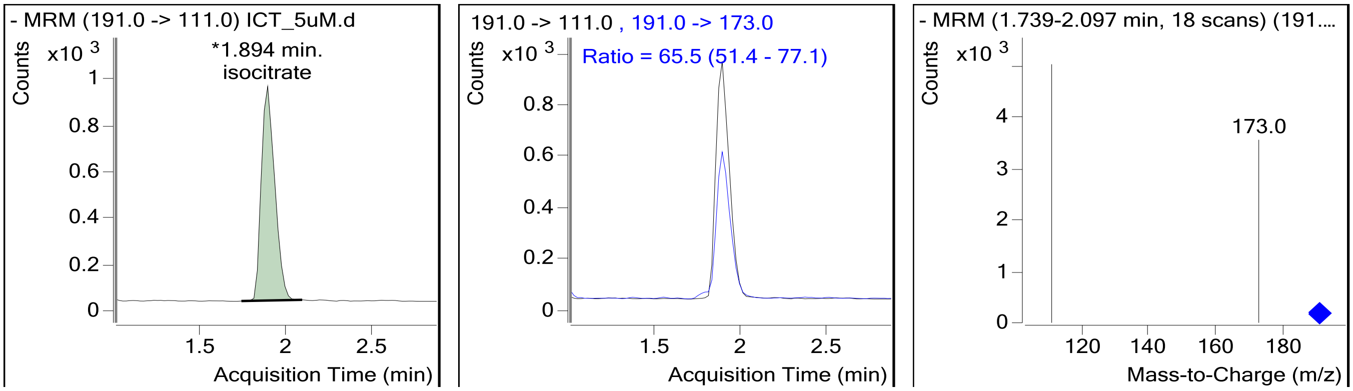

Quantitative Analysis Sample Report

**Batch Results**	D:\MassHunter\Data\Users\062023\Walstrom\LCMS6_13\QuantResults\ICT_6_13analysis_batch.batch.bin		
**Analysis Time**	6/14/2023 11:03 AM	**Analyst Name**	admin
**Report Time**	6/14/2023 11:04 AM	**Reporter Name**	admin
**Last Calib Update**	6/14/2023 11:03 AM	**Batch State**	Processed

Analysis Info

**Acq Time**	2023-06-13 23:56	**Data File**	ICT_12uM.d
**Position**	P1-E4	**Sample Name**	ICT_12
**Dilution**	1	**Sample Info**	
**Inj. Volume**	Per method	**Acq Method File**	NCF_LCMS_neg_IDH.m
**Sample Type**	Calibration	**Comment**	

Sample Chromatogram

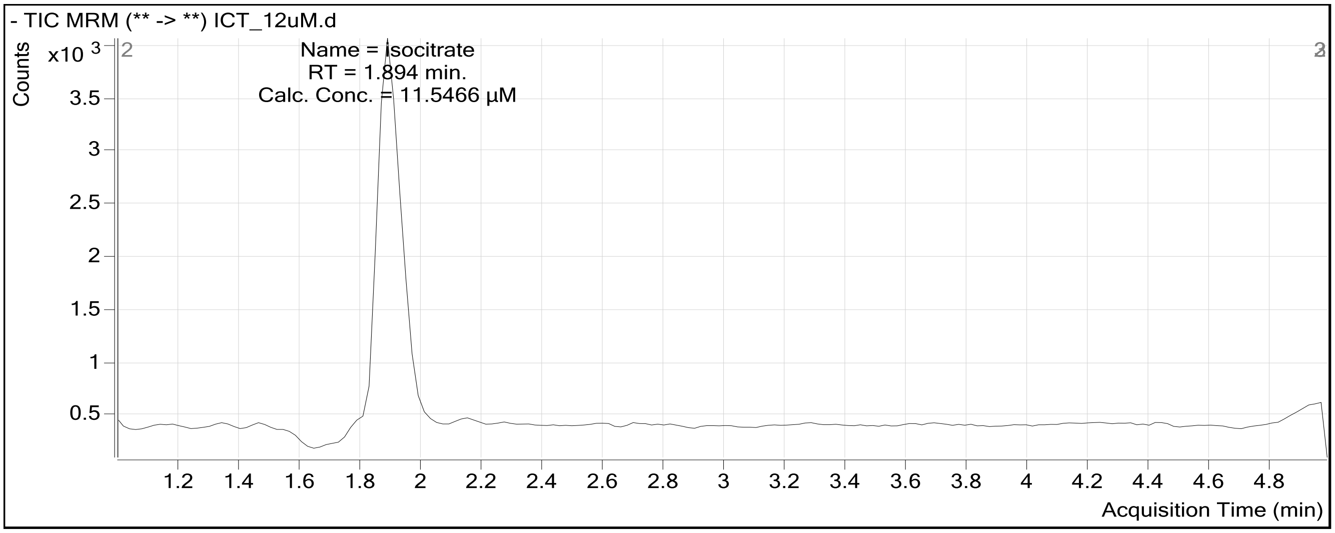

Quantitation Results

Compound	ISTD	RT	Response	ISTD Resp	RR	Conc.	Accuracy
isocitrate		1.894	12324			11.55	96.22

Compound Graphics

Target Compound *isocitrate*

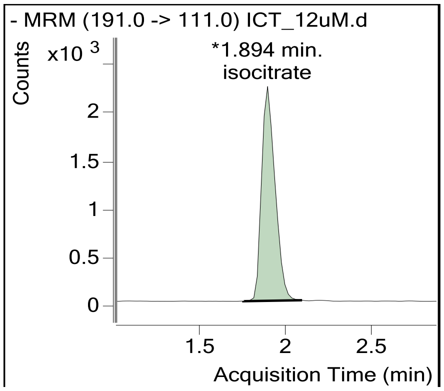

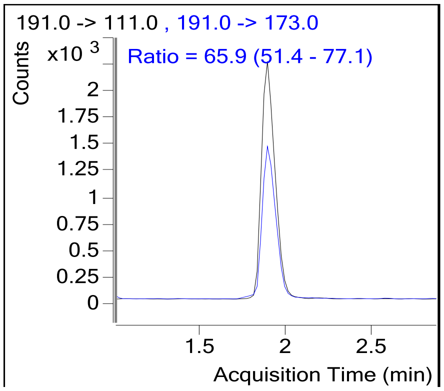

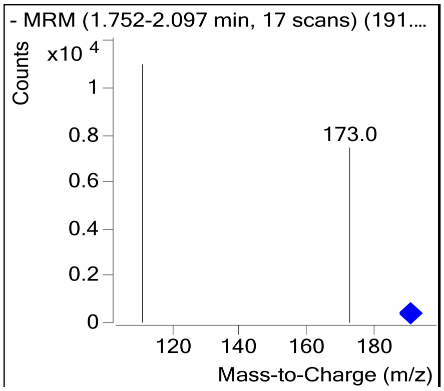

Quantitative Analysis Sample Report

**Batch Results**	D:\MassHunter\Data\Users\062023\Walstrom\LCMS6_13\QuantResults\ICT_6_13analysis_batch.batch.bin		
**Analysis Time**	6/14/2023 11:03 AM	**Analyst Name**	admin
**Report Time**	6/14/2023 11:04 AM	**Reporter Name**	admin
**Last Calib Update**	6/14/2023 11:03 AM	**Batch State**	Processed

Analysis Info

**Acq Time**	2023-06-14 00:06	**Data File**	ICT_25uM.d
**Position**	P1-E5	**Sample Name**	ICT_25
**Dilution**	1	**Sample Info**	
**Inj. Volume**	Per method	**Acq Method File**	NCF_LCMS_neg_IDH.m
**Sample Type**	Calibration	**Comment**	

Sample Chromatogram

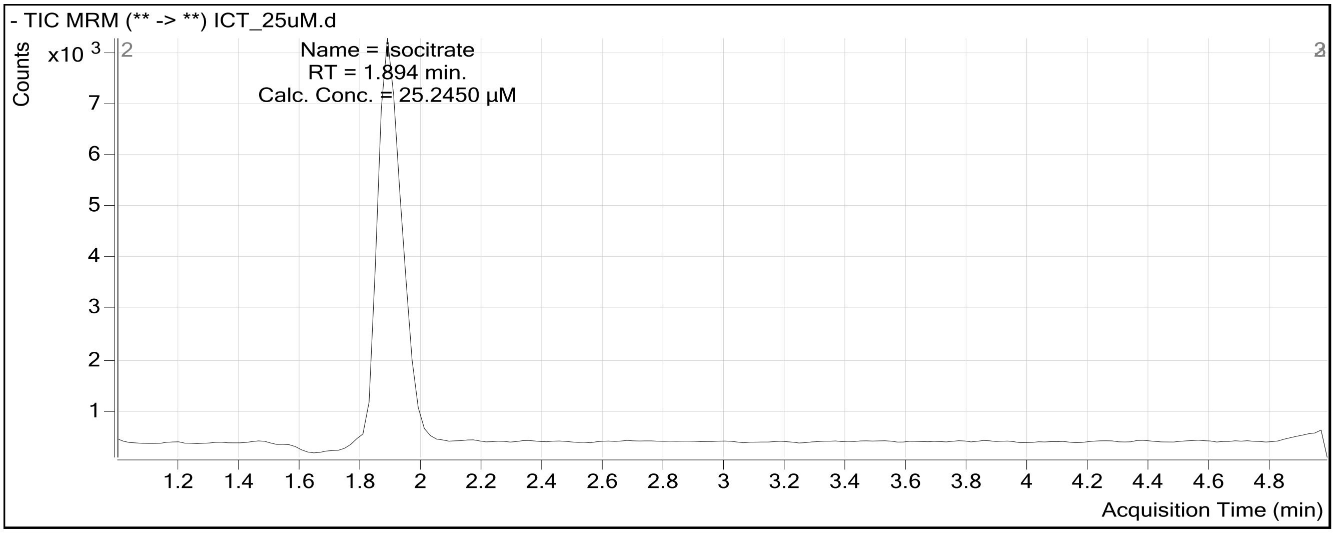

Quantitation Results

Compound	ISTD	RT	Response	ISTD Resp	RR	Conc.	Accuracy
isocitrate		1.894	27102			25.24	100.98

Compound Graphics

Target Compound *isocitrate*

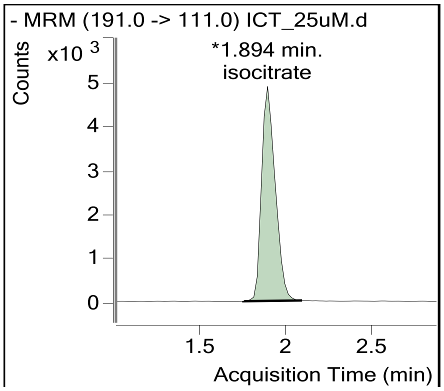

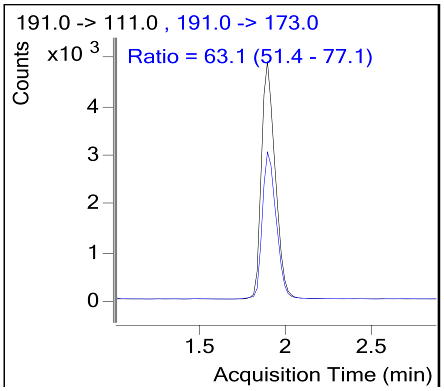

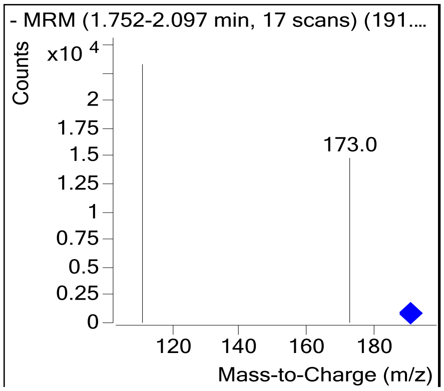

Quantitative Analysis Sample Report

**Batch Results**	D:\MassHunter\Data\Users\062023\Walstrom\LCMS6_13\QuantResults\ICT_6_13analysis_batch.batch.bin		
**Analysis Time**	6/14/2023 11:03 AM	**Analyst Name**	admin
**Report Time**	6/14/2023 11:04 AM	**Reporter Name**	admin
**Last Calib Update**	6/14/2023 11:03 AM	**Batch State**	Processed

Analysis Info

**Acq Time**	2023-06-13 17:46	**Data File**	blank13_50mM_no_wash.d
**Position**	P1-A3	**Sample Name**	blank_50mM_13
**Dilution**	1	**Sample Info**	
**Inj. Volume**	Per method	**Acq Method File**	NCF_LCMS_neg_IDH.m
**Sample Type**	Blank	**Comment**	

Sample Chromatogram

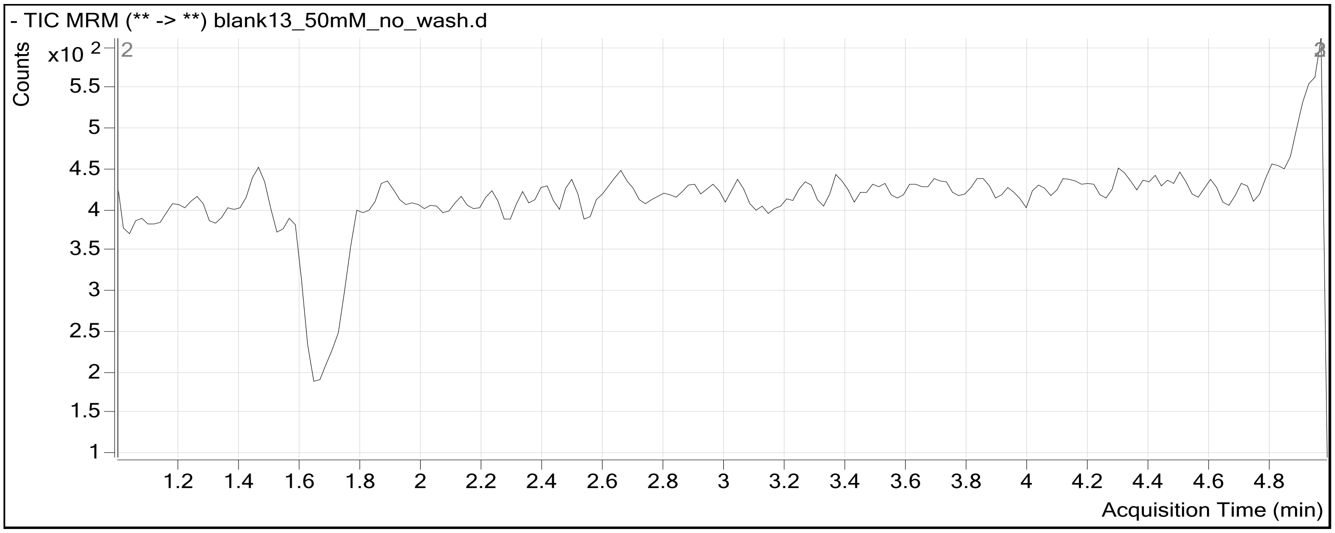

Quantitation Results

Compound	ISTD	RT	Response	ISTD Resp	RR	Conc.	Accuracy
isocitrate						0.00	

Compound Graphics

Target Compound *isocitrate*

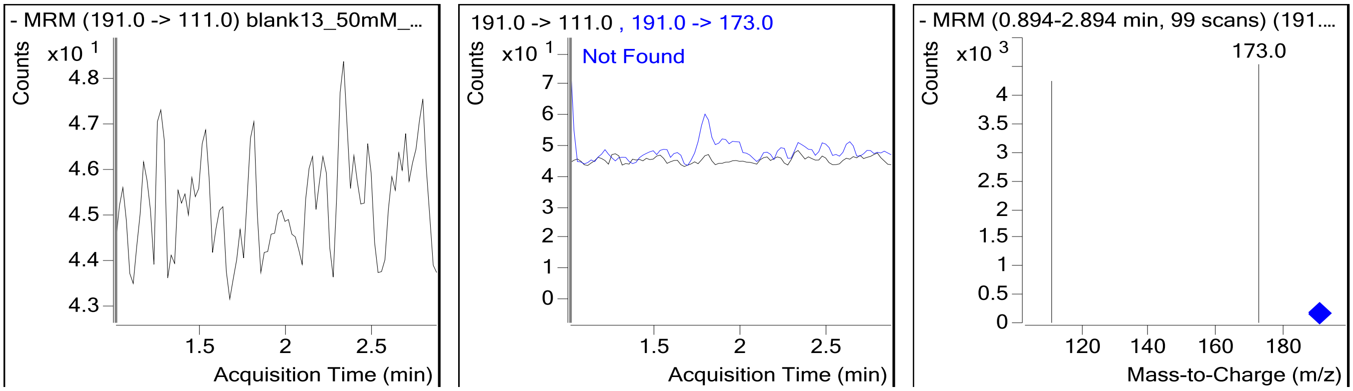

Quantitative Analysis Sample Report

**Batch Results**	D:\MassHunter\Data\Users\062023\Walstrom\LCMS6_13\QuantResults\ICT_6_13analysis_batch.batch.bin		
**Analysis Time**	6/14/2023 11:03 AM	**Analyst Name**	admin
**Report Time**	6/14/2023 11:04 AM	**Reporter Name**	admin
**Last Calib Update**	6/14/2023 11:03 AM	**Batch State**	Processed

Analysis Info

**Acq Time**	2023-06-13 18:40	**Data File**	blank15_50mM_no_wash.d
**Position**	P1-A7	**Sample Name**	blank_50mM_15
**Dilution**	1	**Sample Info**	
**Inj. Volume**	Per method	**Acq Method File**	NCF_LCMS_neg_IDH.m
**Sample Type**	Blank	**Comment**	

Sample Chromatogram

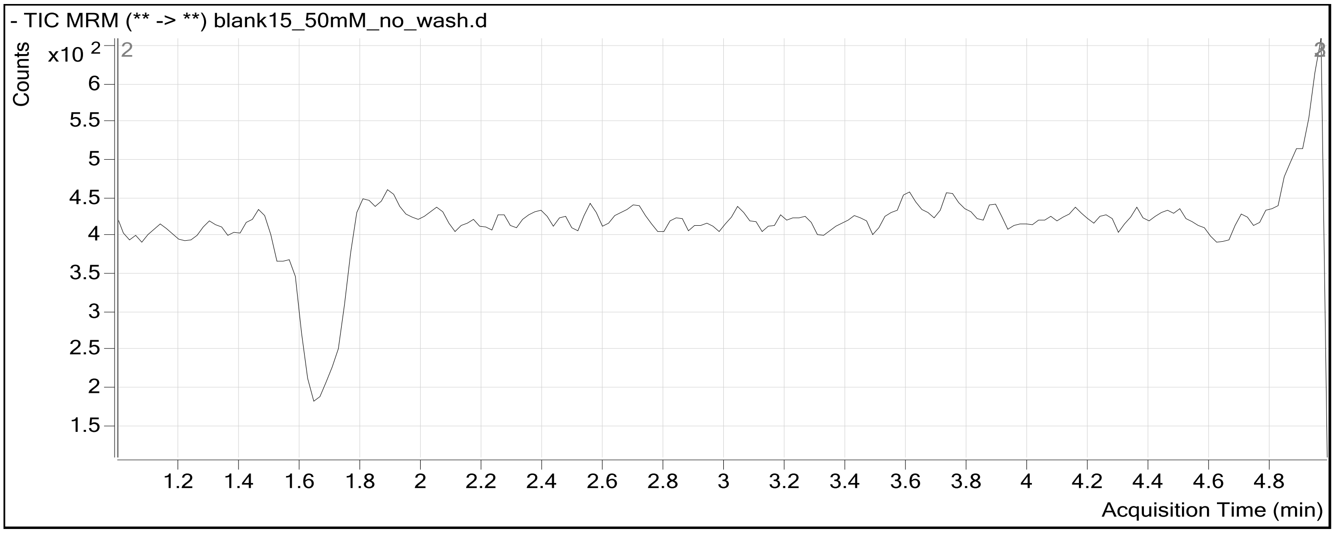

Quantitation Results

Compound	ISTD	RT	Response	ISTD Resp	RR	Conc.	Accuracy
isocitrate						0.00	

Compound Graphics

Target Compound *isocitrate*

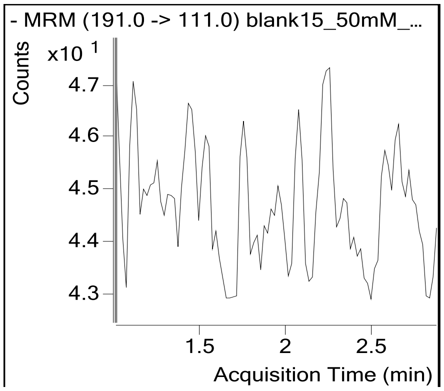

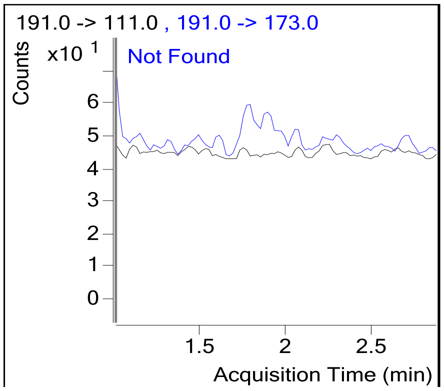

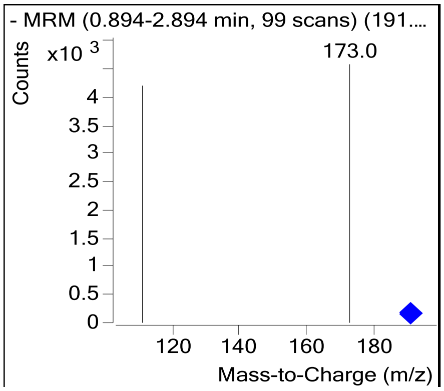

Quantitative Analysis Sample Report

**Batch Results**	D:\MassHunter\Data\Users\062023\Walstrom\LCMS6_13\QuantResults\ICT_6_13analysis_batch.batch.bin		
**Analysis Time**	6/14/2023 11:03 AM	**Analyst Name**	admin
**Report Time**	6/14/2023 11:04 AM	**Reporter Name**	admin
**Last Calib Update**	6/14/2023 11:03 AM	**Batch State**	Processed

Analysis Info

**Acq Time**	2023-06-13 19:24	**Data File**	blank17_50mM_no_wash.d
**Position**	P1-B3	**Sample Name**	blank_50mM_17
**Dilution**	1	**Sample Info**	
**Inj. Volume**	Per method	**Acq Method File**	NCF_LCMS_neg_IDH.m
**Sample Type**	Blank	**Comment**	

Sample Chromatogram

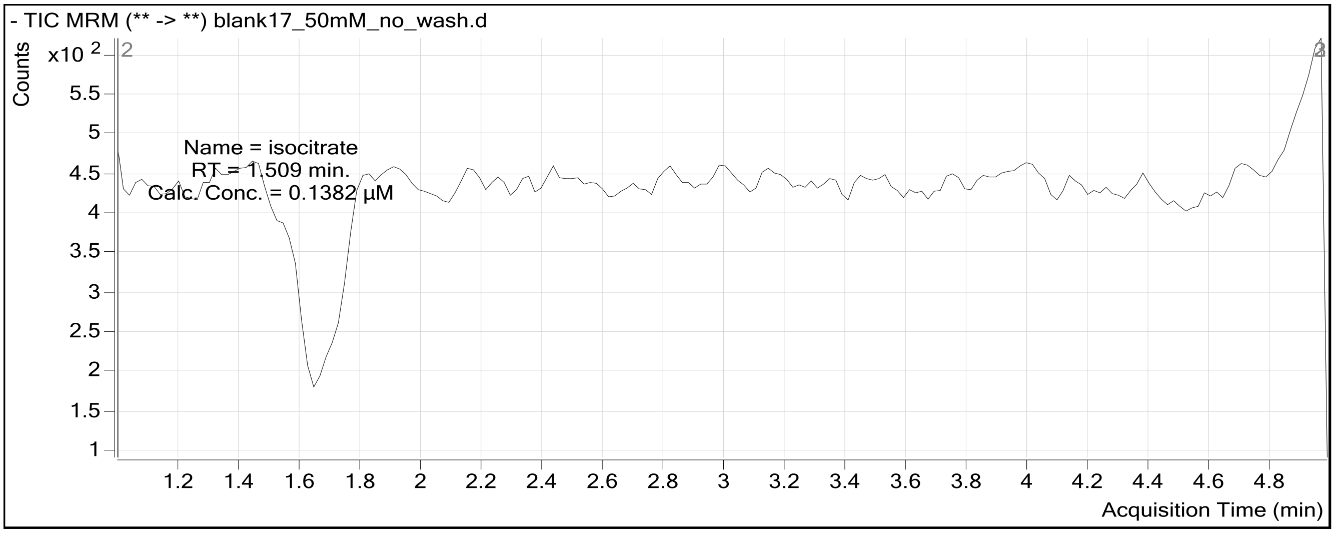

Quantitation Results

Compound	ISTD	RT	Response	ISTD Resp	RR	Conc.	Accuracy
isocitrate		1.509	17			0.14	

Compound Graphics

Target Compound *isocitrate*

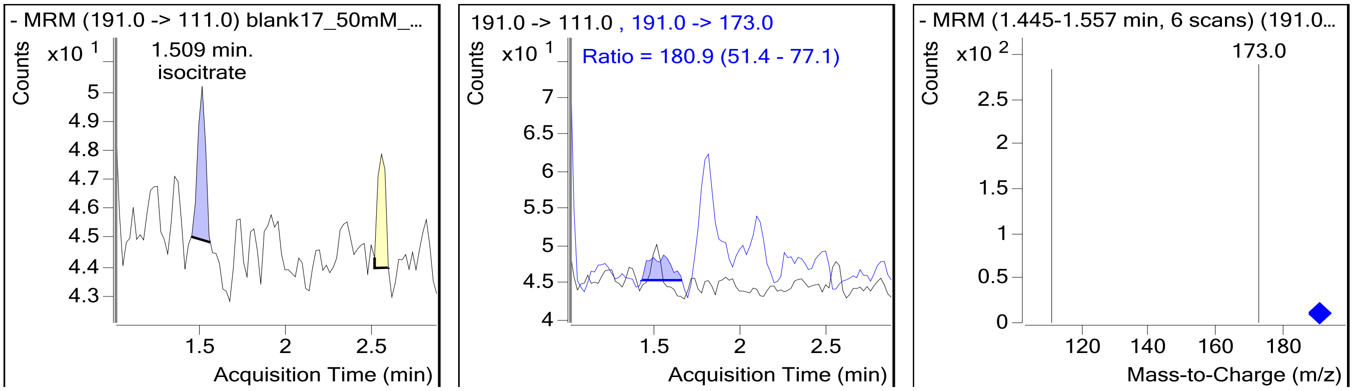

Quantitative Analysis Sample Report

**Batch Results**	D:\MassHunter\Data\Users\062023\Walstrom\LCMS6_13\QuantResults\ICT_6_13analysis_batch.batch.bin		
**Analysis Time**	6/14/2023 11:03 AM	**Analyst Name**	admin
**Report Time**	6/14/2023 11:04 AM	**Reporter Name**	admin
**Last Calib Update**	6/14/2023 11:03 AM	**Batch State**	Processed

Analysis Info

**Acq Time**	2023-06-13 20:07	**Data File**	blank18_50mM_no_wash.d
**Position**	P1-B7	**Sample Name**	blank_50mM_18
**Dilution**	1	**Sample Info**	
**Inj. Volume**	Per method	**Acq Method File**	NCF_LCMS_neg_IDH.m
**Sample Type**	Blank	**Comment**	

Sample Chromatogram

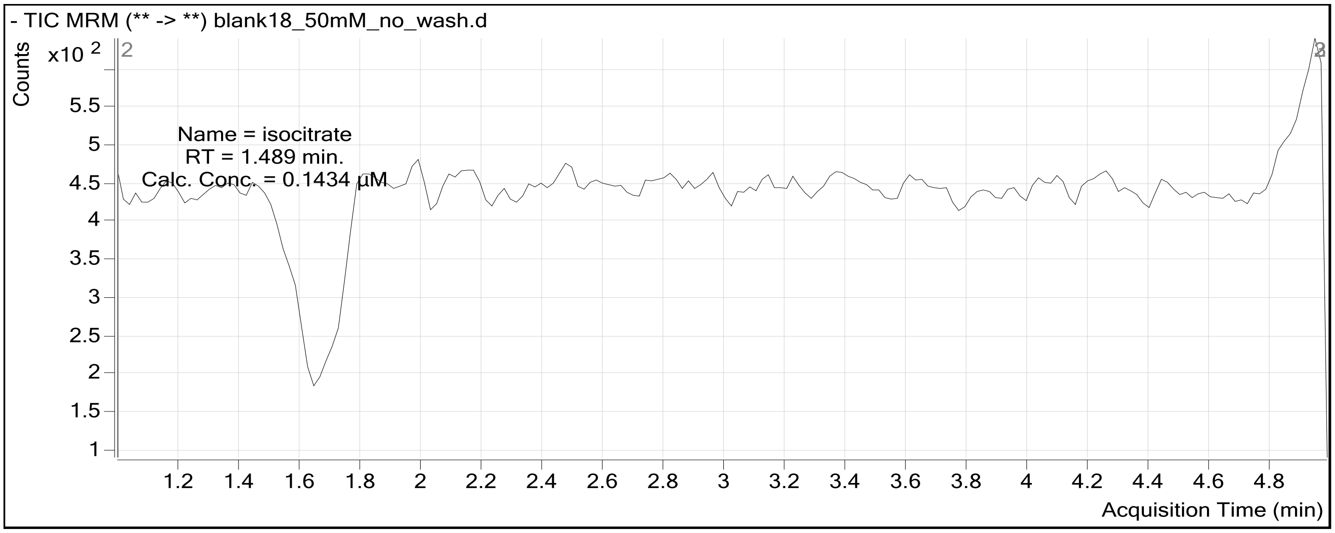

Quantitation Results

Compound	ISTD	RT	Response	ISTD Resp	RR	Conc.	Accuracy
isocitrate		1.489	22			0.14	

Compound Graphics

Target Compound *isocitrate*

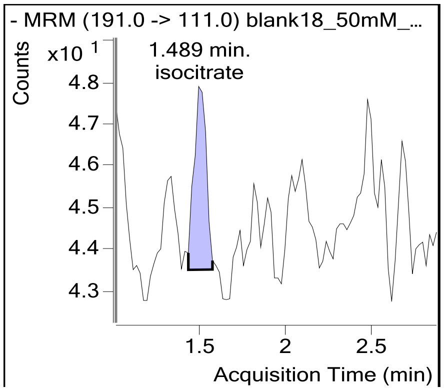

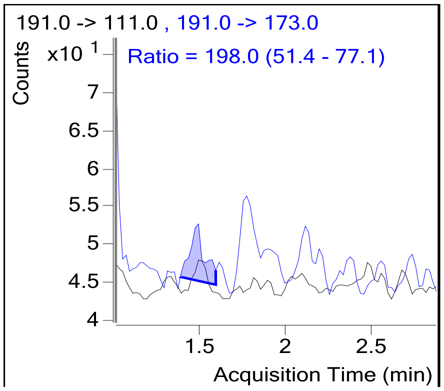

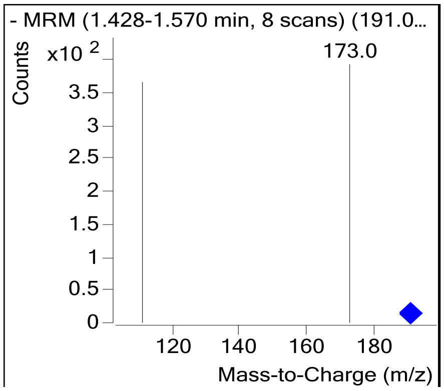

Quantitative Analysis Sample Report

**Batch Results**	D:\MassHunter\Data\Users\062023\Walstrom\LCMS6_13\QuantResults\ICT_6_13analysis_batch.batch.bin		
**Analysis Time**	6/14/2023 11:03 AM	**Analyst Name**	admin
**Report Time**	6/14/2023 11:04 AM	**Reporter Name**	admin
**Last Calib Update**	6/14/2023 11:03 AM	**Batch State**	Processed

Analysis Info

**Acq Time**	2023-06-13 14:32	**Data File**	WT_1a.d
**Position**	P1-A7	**Sample Name**	WT_1a
**Dilution**	1	**Sample Info**	
**Inj. Volume**	Per method	**Acq Method File**	NCF_LCMS_neg_IDH.m
**Sample Type**	Sample	**Comment**	

Sample Chromatogram

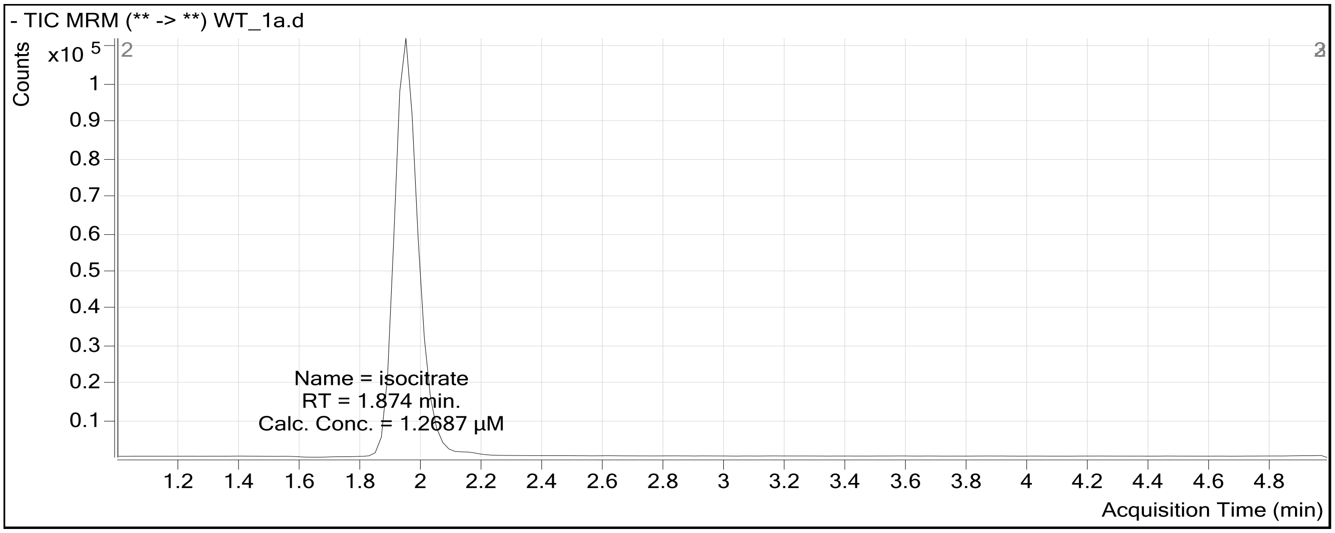

Quantitation Results

Compound	ISTD	RT	Response	ISTD Resp	RR	Conc.	Accuracy
isocitrate		1.874	1236			1.27	

Compound Graphics

Target Compound *isocitrate*

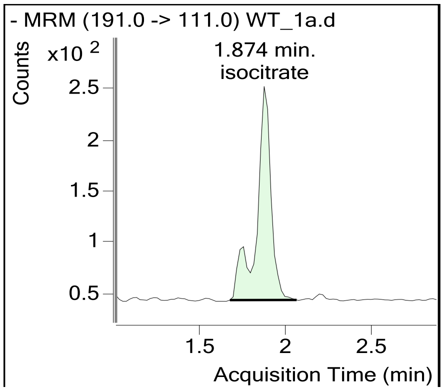

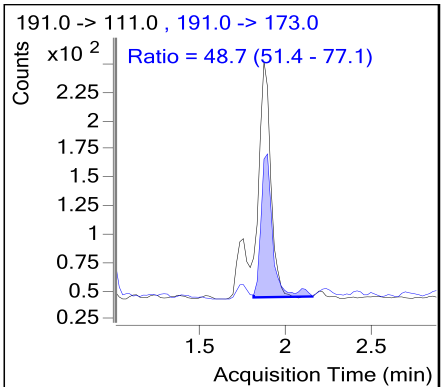

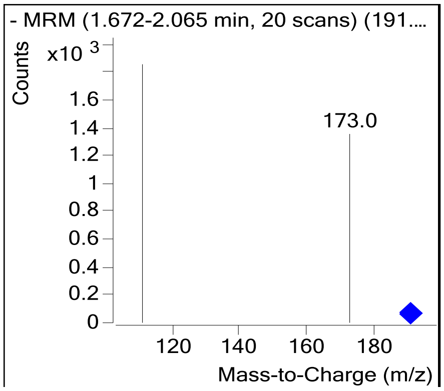

Quantitative Analysis Sample Report

**Batch Results**	D:\MassHunter\Data\Users\062023\Walstrom\LCMS6_13\QuantResults\ICT_6_13analysis_batch.batch.bin		
**Analysis Time**	6/14/2023 11:03 AM	**Analyst Name**	admin
**Report Time**	6/14/2023 11:04 AM	**Reporter Name**	admin
**Last Calib Update**	6/14/2023 11:03 AM	**Batch State**	Processed

Analysis Info

**Acq Time**	2023-06-13 15:05	**Data File**	G98_1a.d
**Position**	P1-A8	**Sample Name**	G98_1a
**Dilution**	1	**Sample Info**	
**Inj. Volume**	Per method	**Acq Method File**	NCF_LCMS_neg_IDH.m
**Sample Type**	Sample	**Comment**	

Sample Chromatogram

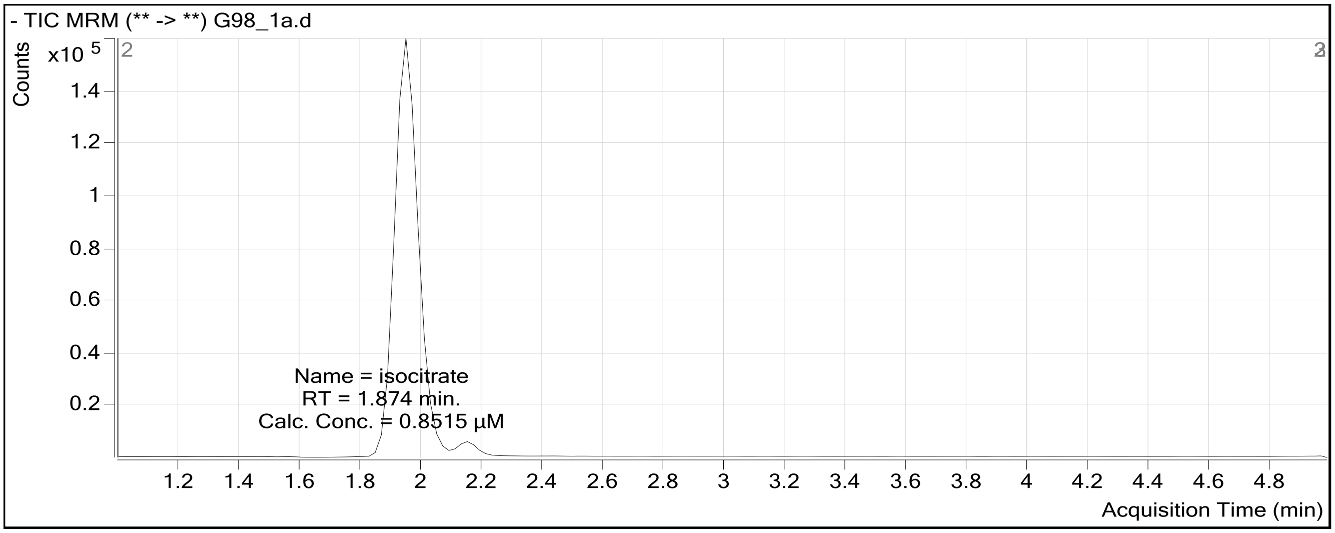

Quantitation Results

Compound	ISTD	RT	Response	ISTD Resp	RR	Conc.	Accuracy
isocitrate		1.874	786			0.85	

Compound Graphics

Target Compound *isocitrate*

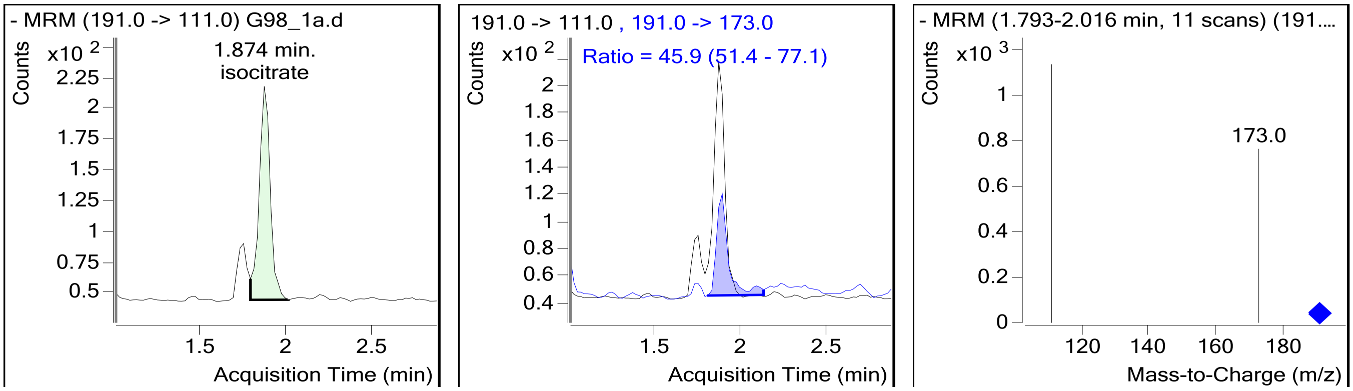

Quantitative Analysis Sample Report

**Batch Results**	D:\MassHunter\Data\Users\062023\Walstrom\LCMS6_13\QuantResults\ICT_6_13analysis_batch.batch.bin		
**Analysis Time**	6/14/2023 11:03 AM	**Analyst Name**	admin
**Report Time**	6/14/2023 11:04 AM	**Reporter Name**	admin
**Last Calib Update**	6/14/2023 11:03 AM	**Batch State**	Processed

Analysis Info

**Acq Time**	2023-06-13 15:26	**Data File**	R133_1.d
**Position**	P1-A9	**Sample Name**	R133_1
**Dilution**	1	**Sample Info**	
**Inj. Volume**	Per method	**Acq Method File**	NCF_LCMS_neg_IDH.m
**Sample Type**	Sample	**Comment**	

Sample Chromatogram

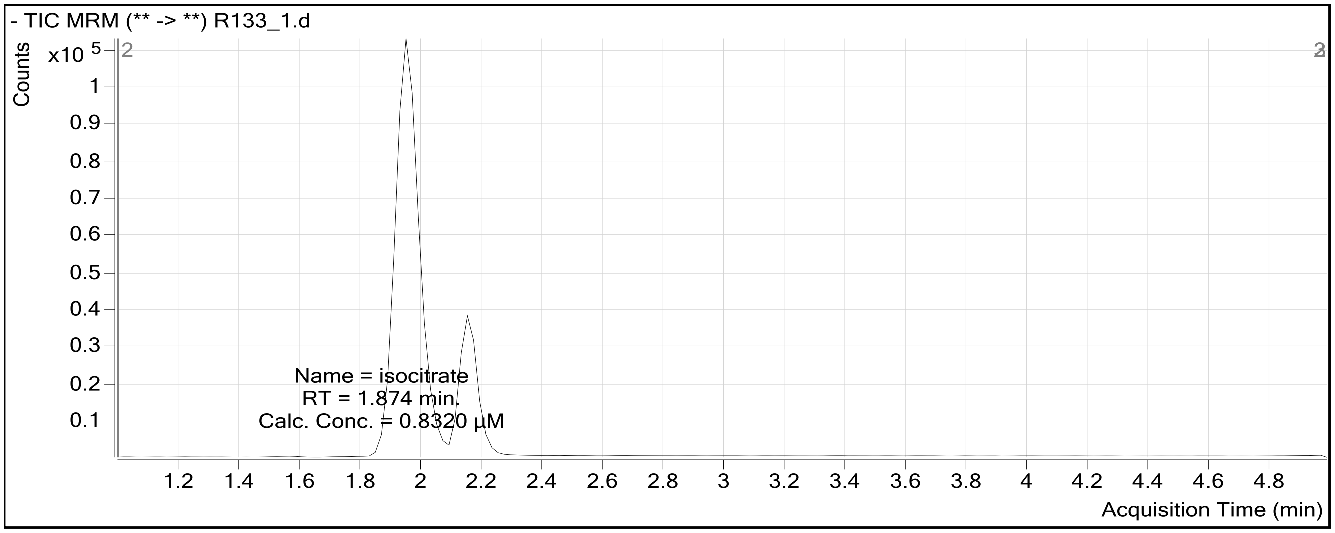

Quantitation Results

Compound	ISTD	RT	Response	ISTD Resp	RR	Conc.	Accuracy
isocitrate		1.874	765			0.83	

Compound Graphics

Target Compound *isocitrate*

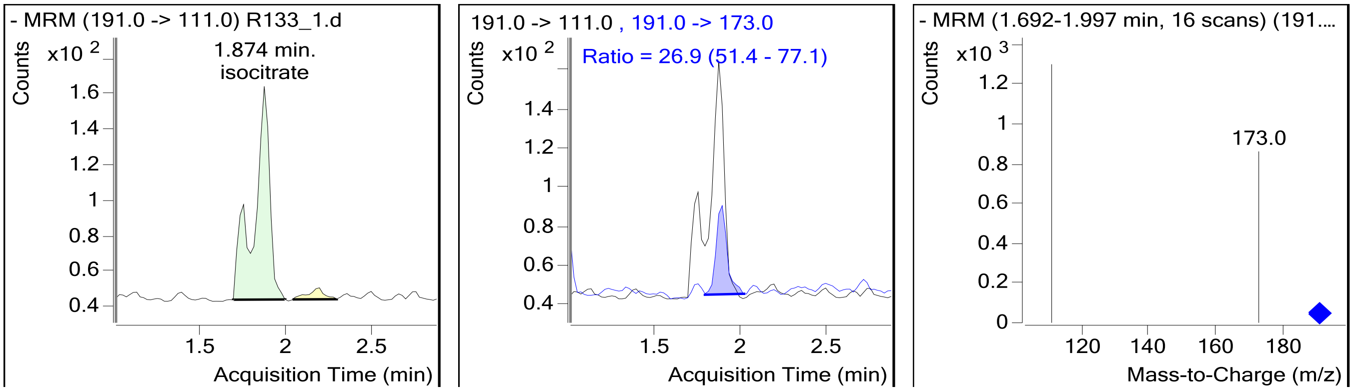

Quantitative Analysis Sample Report

**Batch Results**	D:\MassHunter\Data\Users\062023\Walstrom\LCMS6_13\QuantResults\ICT_6_13analysis_batch.batch.bin		
**Analysis Time**	6/14/2023 11:03 AM	**Analyst Name**	admin
**Report Time**	6/14/2023 11:04 AM	**Reporter Name**	admin
**Last Calib Update**	6/14/2023 11:03 AM	**Batch State**	Processed

Analysis Info

**Acq Time**	2023-06-13 15:59	**Data File**	G98_1a_slow.d
**Position**	P1-A8	**Sample Name**	G98_1a
**Dilution**	1	**Sample Info**	
**Inj. Volume**	Per method	**Acq Method File**	NCF_LCMS_neg_IDH.m
**Sample Type**	Sample	**Comment**	

Sample Chromatogram

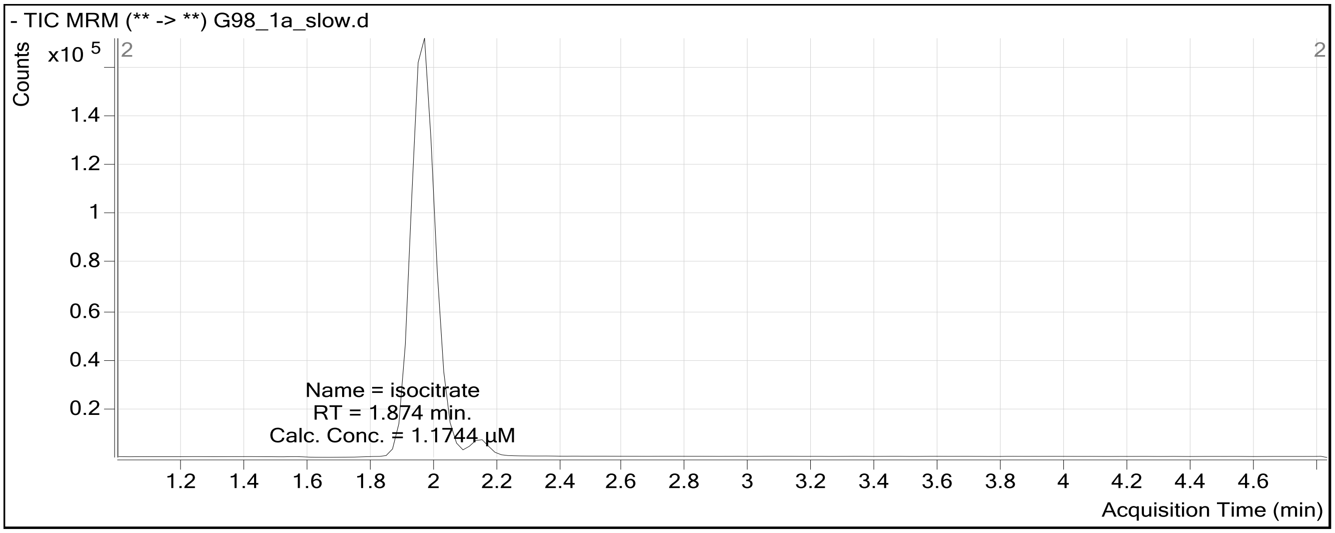

Quantitation Results

Compound	ISTD	RT	Response	ISTD Resp	RR	Conc.	Accuracy
isocitrate		1.874	1134			1.17	

Compound Graphics

Target Compound *isocitrate*

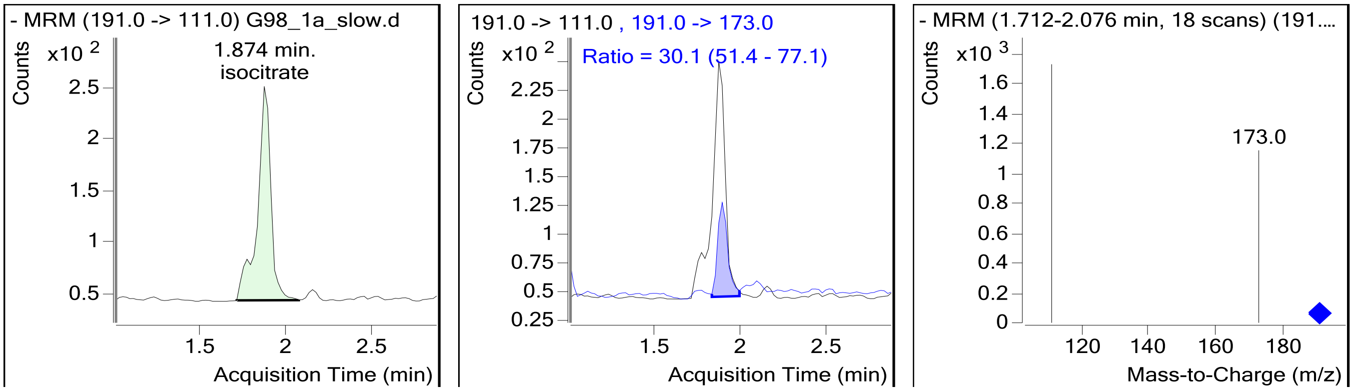

Quantitative Analysis Sample Report

**Batch Results**	D:\MassHunter\Data\Users\062023\Walstrom\LCMS6_13\QuantResults\ICT_6_13analysis_batch.batch.bin		
**Analysis Time**	6/14/2023 11:03 AM	**Analyst Name**	admin
**Report Time**	6/14/2023 11:04 AM	**Reporter Name**	admin
**Last Calib Update**	6/14/2023 11:03 AM	**Batch State**	Processed

Analysis Info

**Acq Time**	2023-06-13 17:57	**Data File**	WT_1a_2nd_run.d
**Position**	P1-A1	**Sample Name**	WT_1a_2
**Dilution**	1	**Sample Info**	
**Inj. Volume**	Per method	**Acq Method File**	NCF_LCMS_neg_IDH.m
**Sample Type**	Sample	**Comment**	

Sample Chromatogram

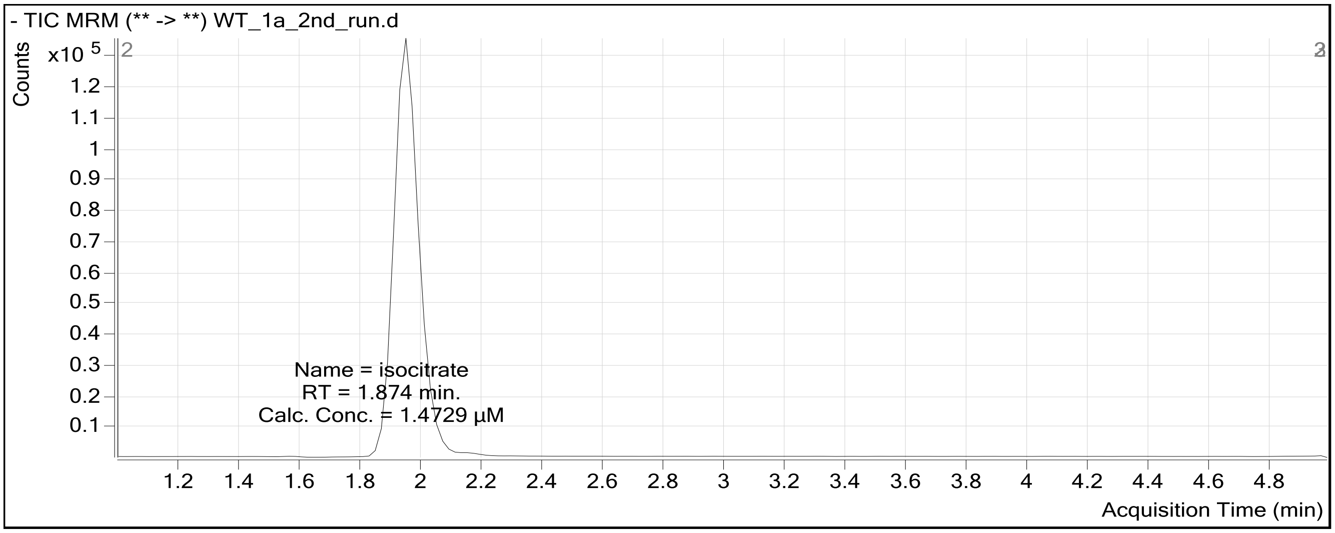

Quantitation Results

Compound	ISTD	RT	Response	ISTD Resp	RR	Conc.	Accuracy
isocitrate		1.874	1456			1.47	

Compound Graphics

Target Compound *isocitrate*

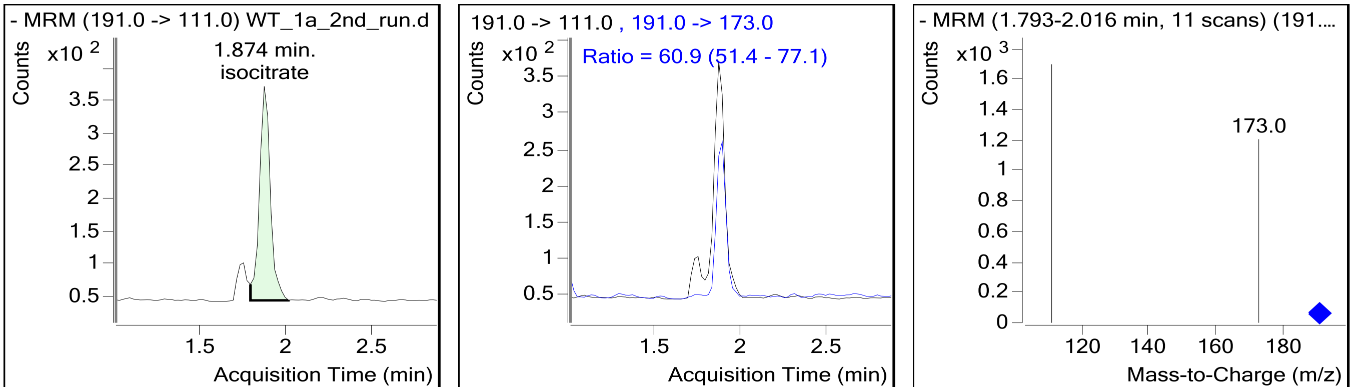

Quantitative Analysis Sample Report

**Batch Results**	D:\MassHunter\Data\Users\062023\Walstrom\LCMS6_13\QuantResults\ICT_6_13analysis_batch.batch.bin		
**Analysis Time**	6/14/2023 11:03 AM	**Analyst Name**	admin
**Report Time**	6/14/2023 11:04 AM	**Reporter Name**	admin
**Last Calib Update**	6/14/2023 11:03 AM	**Batch State**	Processed

Analysis Info

**Acq Time**	2023-06-13 18:08	**Data File**	WT_1b.d
**Position**	P1-A4	**Sample Name**	WT_1b
**Dilution**	1	**Sample Info**	
**Inj. Volume**	Per method	**Acq Method File**	NCF_LCMS_neg_IDH.m
**Sample Type**	Sample	**Comment**	

Sample Chromatogram

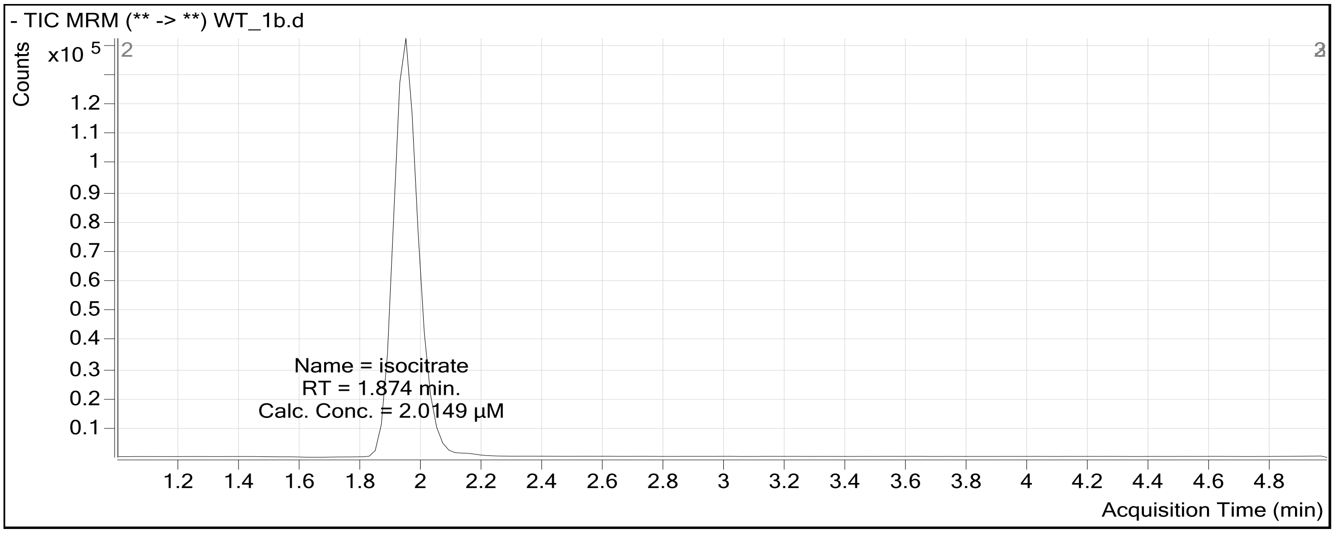

Quantitation Results

Compound	ISTD	RT	Response	ISTD Resp	RR	Conc.	Accuracy
isocitrate		1.874	2041			2.01	

Compound Graphics

Target Compound *isocitrate*

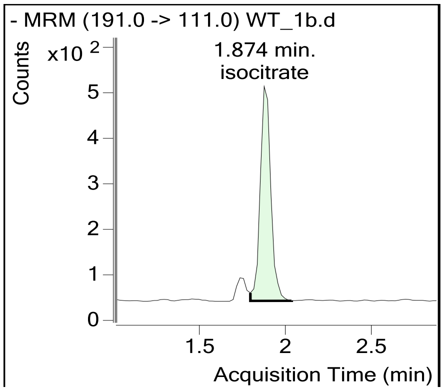

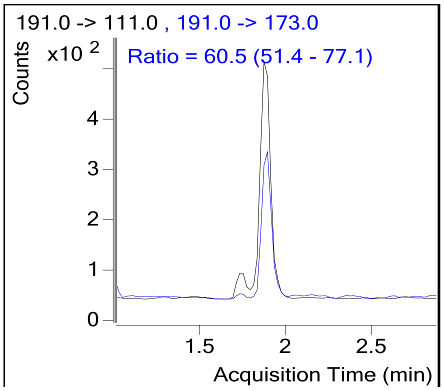

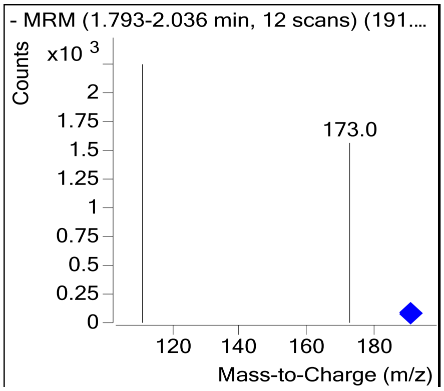

Quantitative Analysis Sample Report

**Batch Results**	D:\MassHunter\Data\Users\062023\Walstrom\LCMS6_13\QuantResults\ICT_6_13analysis_batch.batch.bin		
**Analysis Time**	6/14/2023 11:03 AM	**Analyst Name**	admin
**Report Time**	6/14/2023 11:04 AM	**Reporter Name**	admin
**Last Calib Update**	6/14/2023 11:03 AM	**Batch State**	Processed

Analysis Info

**Acq Time**	2023-06-13 18:19	**Data File**	WT_2.d
**Position**	P1-A5	**Sample Name**	WT_2
**Dilution**	1	**Sample Info**	
**Inj. Volume**	Per method	**Acq Method File**	NCF_LCMS_neg_IDH.m
**Sample Type**	Sample	**Comment**	

Sample Chromatogram

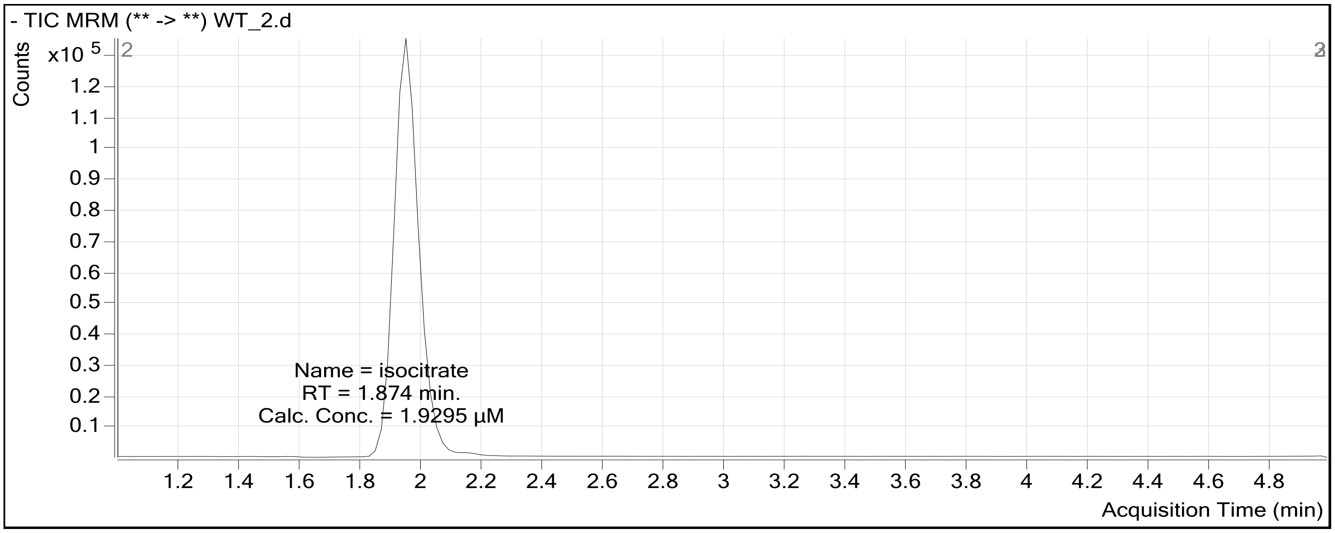

Quantitation Results

Compound	ISTD	RT	Response	ISTD Resp	RR	Conc.	Accuracy
isocitrate		1.874	1949			1.93	

Compound Graphics

Target Compound *isocitrate*

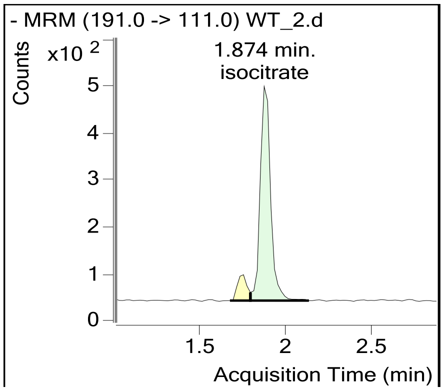

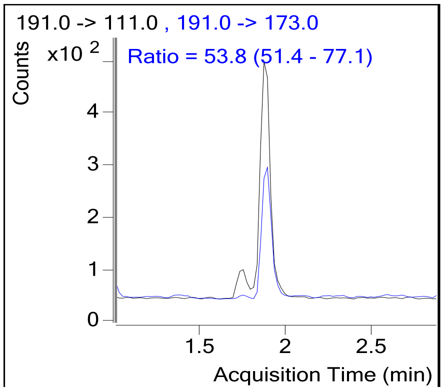

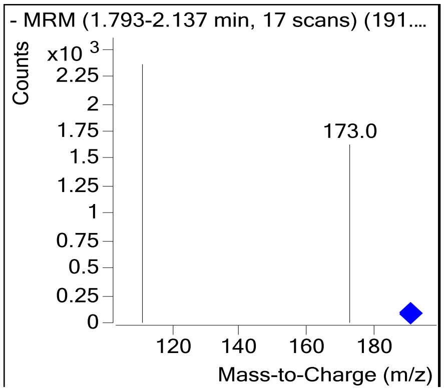

Quantitative Analysis Sample Report

**Batch Results**	D:\MassHunter\Data\Users\062023\Walstrom\LCMS6_13\QuantResults\ICT_6_13analysis_batch.batch.bin		
**Analysis Time**	6/14/2023 11:03 AM	**Analyst Name**	admin
**Report Time**	6/14/2023 11:04 AM	**Reporter Name**	admin
**Last Calib Update**	6/14/2023 11:03 AM	**Batch State**	Processed

Analysis Info

**Acq Time**	2023-06-13 18:51	**Data File**	G98_1b.d
**Position**	P1-A8	**Sample Name**	G98_1b
**Dilution**	1	**Sample Info**	
**Inj. Volume**	Per method	**Acq Method File**	NCF_LCMS_neg_IDH.m
**Sample Type**	Sample	**Comment**	

Sample Chromatogram

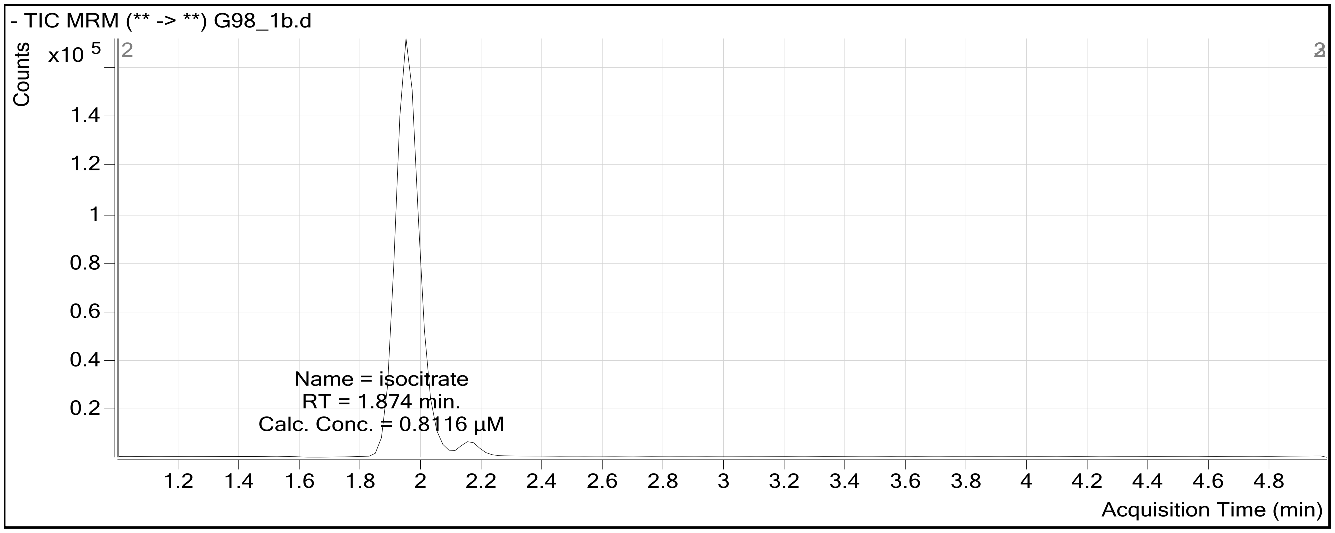

Quantitation Results

Compound	ISTD	RT	Response	ISTD Resp	RR	Conc.	Accuracy
isocitrate		1.874	743			0.81	

Compound Graphics

Target Compound *isocitrate*

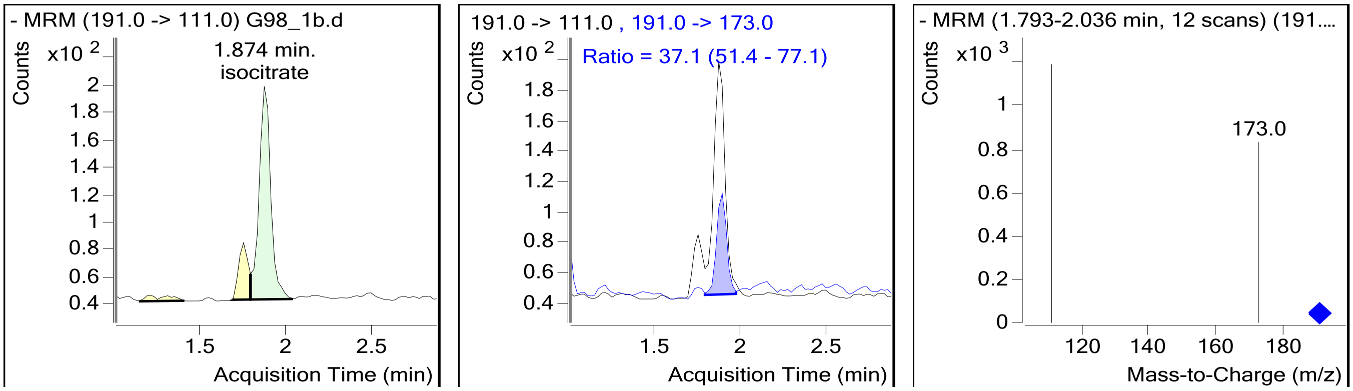

Quantitative Analysis Sample Report

**Batch Results**	D:\MassHunter\Data\Users\062023\Walstrom\LCMS6_13\QuantResults\ICT_6_13analysis_batch.batch.bin		
**Analysis Time**	6/14/2023 11:03 AM	**Analyst Name**	admin
**Report Time**	6/14/2023 11:04 AM	**Reporter Name**	admin
**Last Calib Update**	6/14/2023 11:03 AM	**Batch State**	Processed

Analysis Info

**Acq Time**	2023-06-13 19:02	**Data File**	G98_2.d
**Position**	P1-B1	**Sample Name**	G98_2
**Dilution**	1	**Sample Info**	
**Inj. Volume**	Per method	**Acq Method File**	NCF_LCMS_neg_IDH.m
**Sample Type**	Sample	**Comment**	

Sample Chromatogram

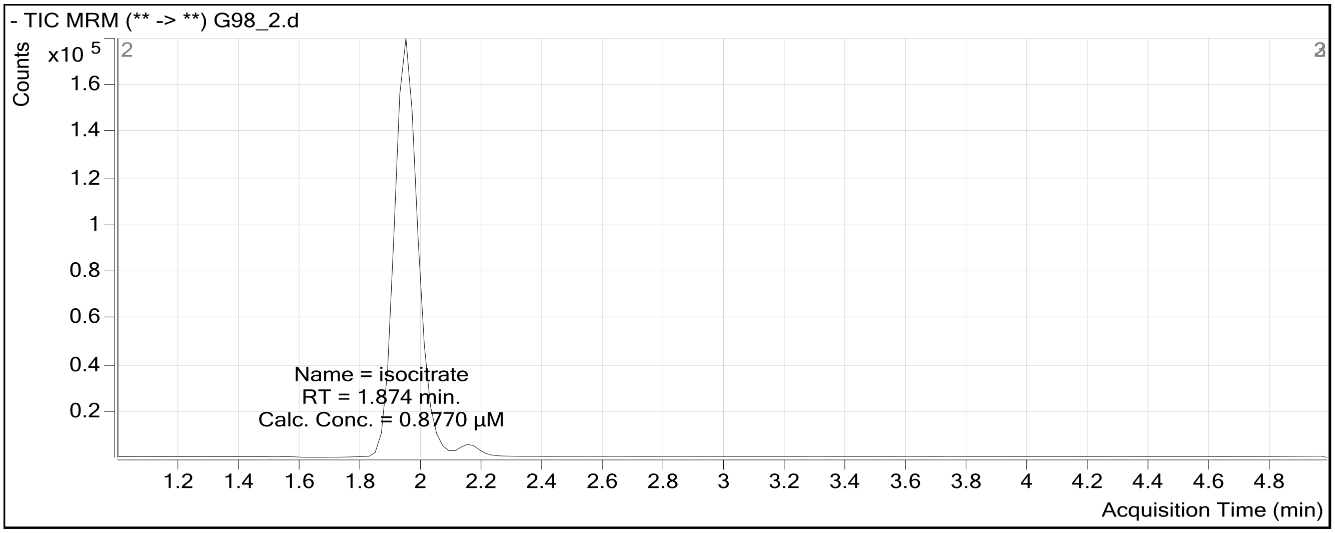

Quantitation Results

Compound	ISTD	RT	Response	ISTD Resp	RR	Conc.	Accuracy
isocitrate		1.874	814			0.88	

Compound Graphics

Target Compound *isocitrate*

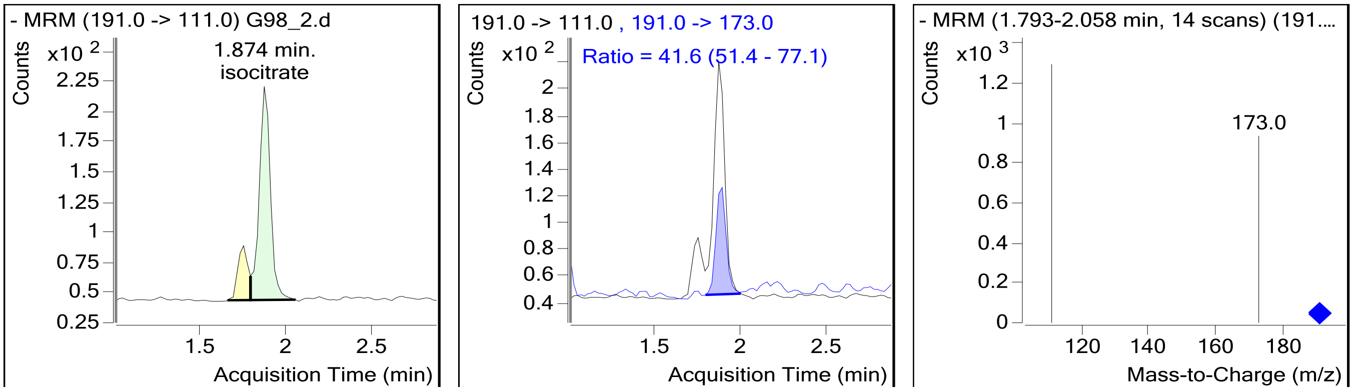

Quantitative Analysis Sample Report

**Batch Results**	D:\MassHunter\Data\Users\062023\Walstrom\LCMS6_13\QuantResults\ICT_6_13analysis_batch.batch.bin		
**Analysis Time**	6/14/2023 11:03 AM	**Analyst Name**	admin
**Report Time**	6/14/2023 11:04 AM	**Reporter Name**	admin
**Last Calib Update**	6/14/2023 11:03 AM	**Batch State**	Processed

Analysis Info

**Acq Time**	2023-06-13 19:35	**Data File**	R133_1_2nd_run.d
**Position**	P1-B4	**Sample Name**	R133_1
**Dilution**	1	**Sample Info**	
**Inj. Volume**	Per method	**Acq Method File**	NCF_LCMS_neg_IDH.m
**Sample Type**	Sample	**Comment**	

Sample Chromatogram

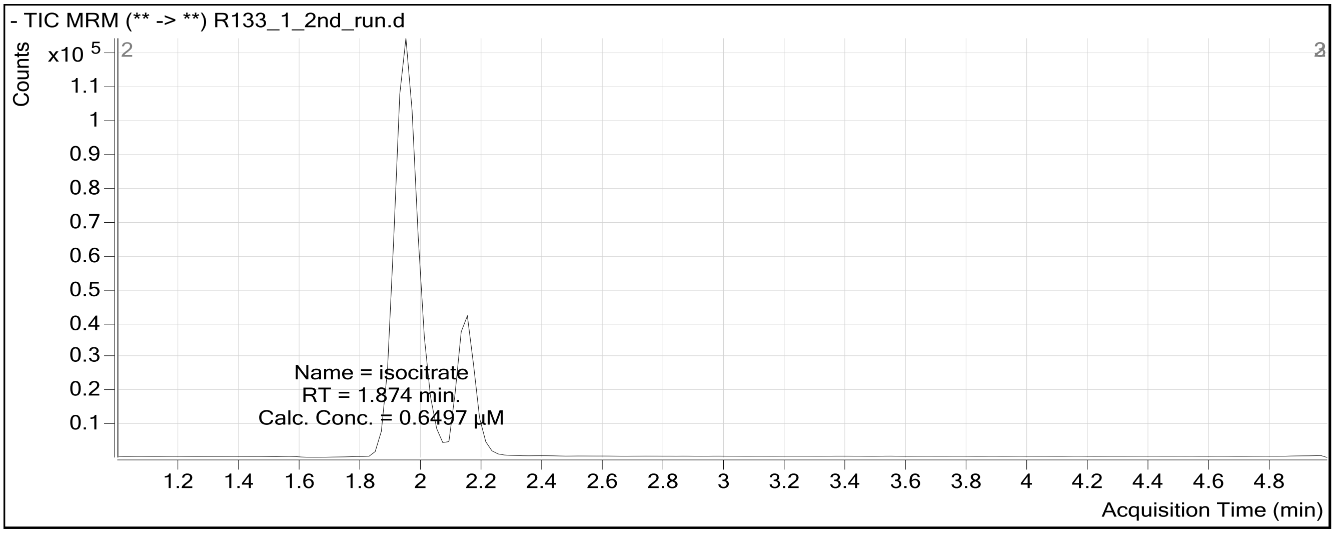

Quantitation Results

Compound	ISTD	RT	Response	ISTD Resp	RR	Conc.	Accuracy
isocitrate		1.874	568			0.65	

Compound Graphics

Target Compound *isocitrate*

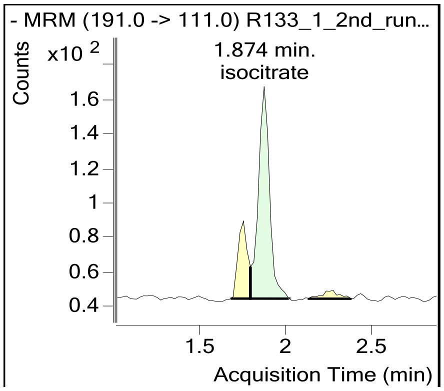

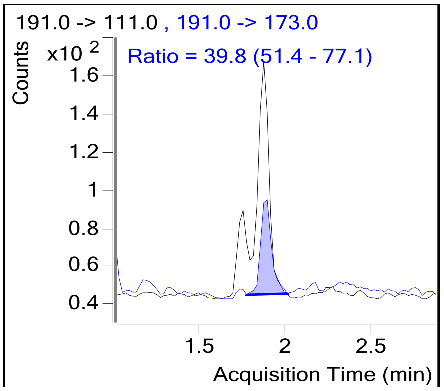

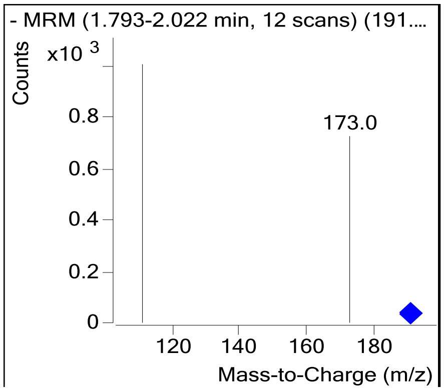

Quantitative Analysis Sample Report

**Batch Results**	D:\MassHunter\Data\Users\062023\Walstrom\LCMS6_13\QuantResults\ICT_6_13analysis_batch.batch.bin		
**Analysis Time**	6/14/2023 11:03 AM	**Analyst Name**	admin
**Report Time**	6/14/2023 11:04 AM	**Reporter Name**	admin
**Last Calib Update**	6/14/2023 11:03 AM	**Batch State**	Processed

Analysis Info

**Acq Time**	2023-06-13 19:46	**Data File**	R133_2.d
**Position**	P1-B5	**Sample Name**	R133_2
**Dilution**	1	**Sample Info**	
**Inj. Volume**	Per method	**Acq Method File**	NCF_LCMS_neg_IDH.m
**Sample Type**	Sample	**Comment**	

Sample Chromatogram

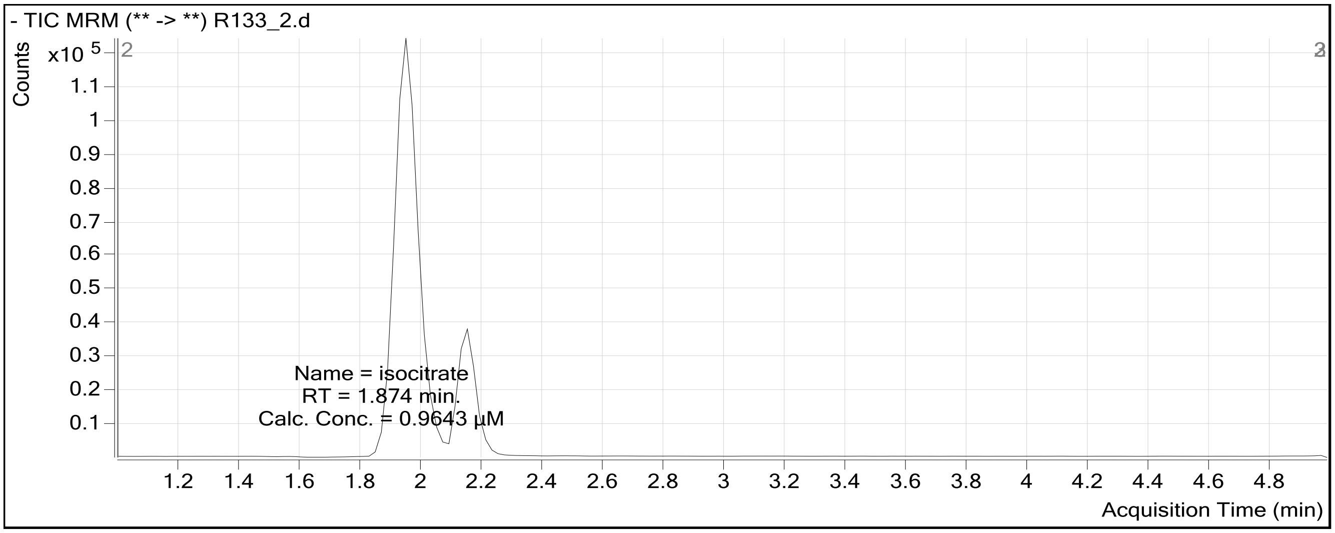

Quantitation Results

Compound	ISTD	RT	Response	ISTD Resp	RR	Conc.	Accuracy
isocitrate		1.874	908			0.96	

Compound Graphics

Target Compound *isocitrate*

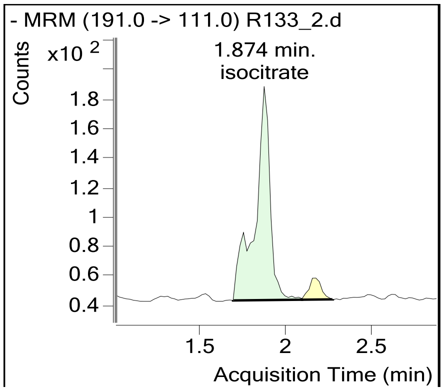

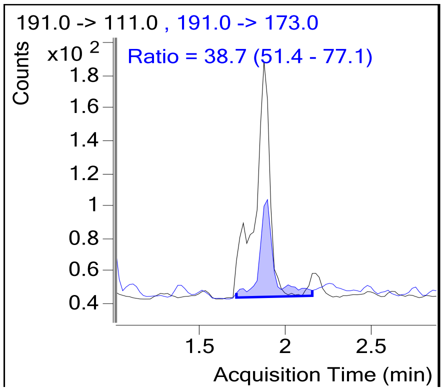

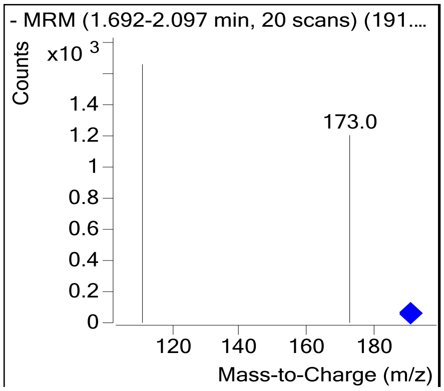

Quantitative Analysis Sample Report

**Batch Results**	D:\MassHunter\Data\Users\062023\Walstrom\LCMS6_13\QuantResults\ICT_6_13analysis_batch.batch.bin		
**Analysis Time**	6/14/2023 11:03 AM	**Analyst Name**	admin
**Report Time**	6/14/2023 11:04 AM	**Reporter Name**	admin
**Last Calib Update**	6/14/2023 11:03 AM	**Batch State**	Processed

Analysis Info

**Acq Time**	2023-06-13 19:57	**Data File**	R133_3.d
**Position**	P1-B6	**Sample Name**	R133_3
**Dilution**	1	**Sample Info**	
**Inj. Volume**	Per method	**Acq Method File**	NCF_LCMS_neg_IDH.m
**Sample Type**	Sample	**Comment**	

Sample Chromatogram

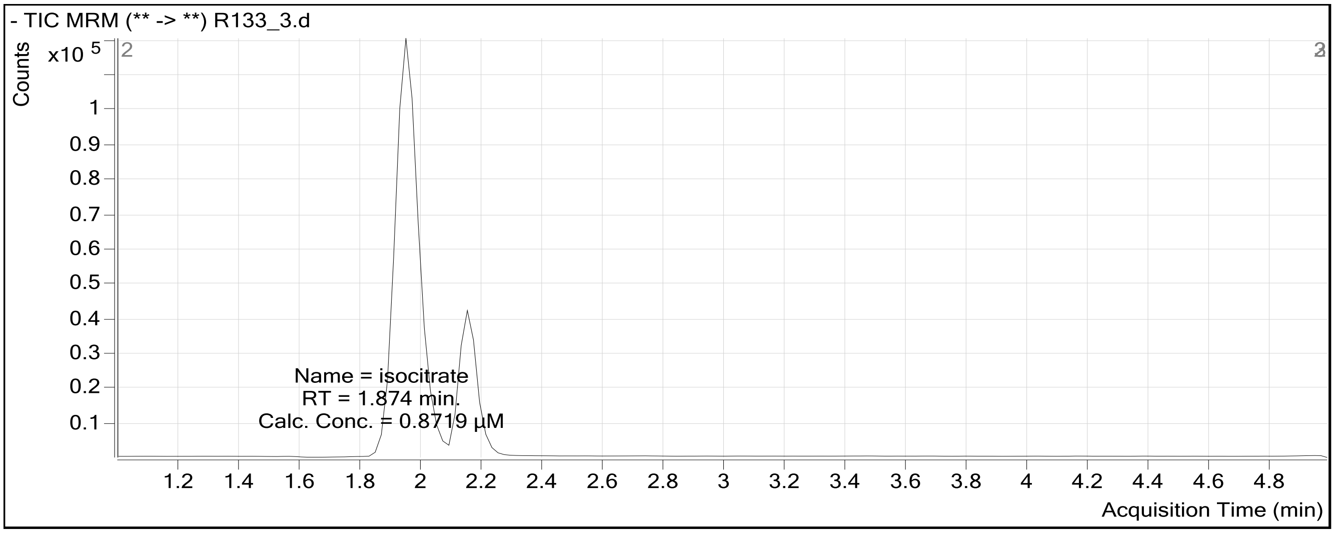

Quantitation Results

Compound	ISTD	RT	Response	ISTD Resp	RR	Conc.	Accuracy
isocitrate		1.874	808			0.87	

Compound Graphics

Target Compound *isocitrate*

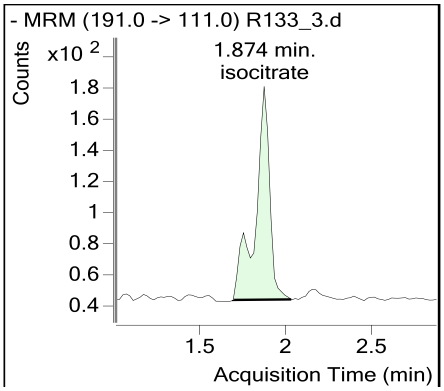

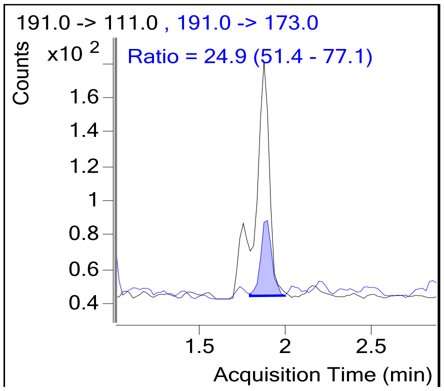

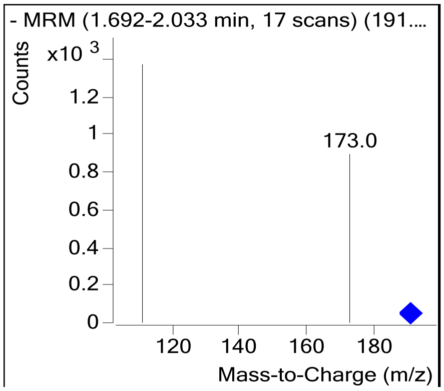

Quantitative Analysis Sample Report

**Batch Results**	D:\MassHunter\Data\Users\062023\Walstrom\LCMS6_13\QuantResults\ICT_6_13analysis_batch.batch.bin		
**Analysis Time**	6/14/2023 11:03 AM	**Analyst Name**	admin
**Report Time**	6/14/2023 11:04 AM	**Reporter Name**	admin
**Last Calib Update**	6/14/2023 11:03 AM	**Batch State**	Processed

Analysis Info

**Acq Time**	2023-06-13 20:18	**Data File**	rxnmix_div_2.d
**Position**	P1-B8	**Sample Name**	rxn_mix_div_2
**Dilution**	1	**Sample Info**	
**Inj. Volume**	Per method	**Acq Method File**	NCF_LCMS_neg_IDH.m
**Sample Type**	Sample	**Comment**	

Sample Chromatogram

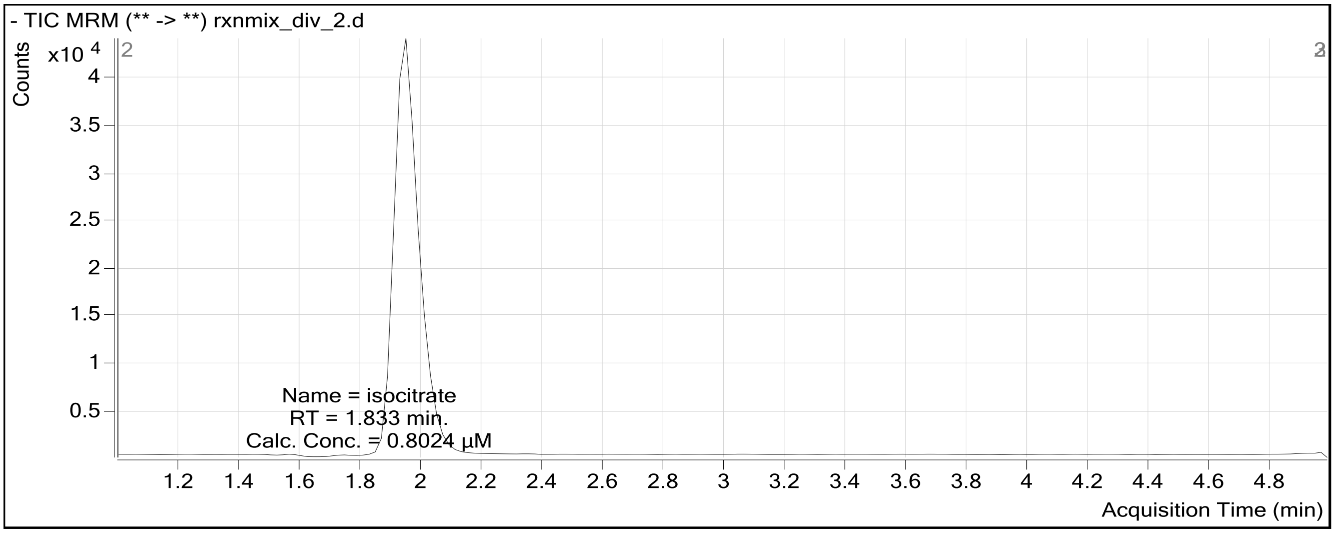

Quantitation Results

Compound	ISTD	RT	Response	ISTD Resp	RR	Conc.	Accuracy
isocitrate		1.833	733			0.80	

Compound Graphics

Target Compound *isocitrate*

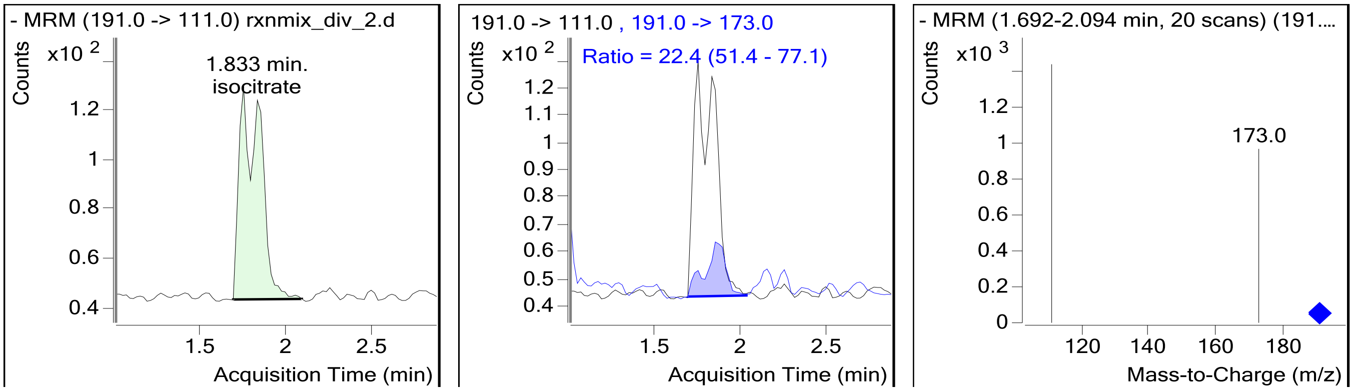

Quantitative Analysis Summary Report

**Batch Results**	D:\MassHunter\Data\Users\062023\Walstrom\LCMS6_13\QuantResults\2-HG_6_13analysis_batch.batch.bin		
**Analysis Time**	6/14/2023 11:06 AM	**Analyst Name**	admin
**Report Time**	6/14/2023 11:13 AM	**Reporter Name**	admin
**Last Calib Update**	6/14/2023 11:06 AM	**Batch State**	Processed
**Quant Batch Version**	B.05.01	**Quant Report Version**	B.05.01

Sequence Table

Data File	Sample Name	Sample Type	Position	Inj. Volume	Level	Acq Method File
2HG_200uM.d	2HG_200	Sample	P1-A1	Per method		NCF_LCMS_neg_IDH.m
2HG_200uM_2.d	2HG_200	Sample	P1-A1	Per method		NCF_LCMS_neg_IDH.m
2HG_0_1uM.d	2HG_0_1	Calibration	P1-F3	Per method	1	NCF_LCMS_neg_IDH.m
2HG_0_5uM.d	2HG_0_5	Calibration	P1-F4	Per method	2	NCF_LCMS_neg_IDH.m
2HG_1uM.d	2HG_1	Calibration	P1-C1	Per method	3	NCF_LCMS_neg_IDH.m
2HG_2uM.d	2HG_2	Calibration	P1-C2	Per method	4	NCF_LCMS_neg_IDH.m
2HG_5uM.d	2HG_5	Calibration	P1-C3	Per method	5	NCF_LCMS_neg_IDH.m
2HG_12uM.d	2HG_12	Calibration	P1-C4	Per method	6	NCF_LCMS_neg_IDH.m
2HG_25uM.d	2HG_25	Calibration	P1-C5	Per method	7	NCF_LCMS_neg_IDH.m
2HG_50uM.d	2HG_50	Calibration	P1-C6	Per method	8	NCF_LCMS_neg_IDH.m
2HG_100uM.d	2HG_100	Calibration	P1-C7	Per method	9	NCF_LCMS_neg_IDH.m
blank13_50mM_no_wash.d	blank_50mM_13	Blank	P1-A3	Per method		NCF_LCMS_neg_IDH.m
blank15_50mM_no_wash.d	blank_50mM_15	Blank	P1-A7	Per method		NCF_LCMS_neg_IDH.m
blank17_50mM_no_wash.d	blank_50mM_17	Blank	P1-B3	Per method		NCF_LCMS_neg_IDH.m
blank18_50mM_no_wash.d	blank_50mM_18	Blank	P1-B7	Per method		NCF_LCMS_neg_IDH.m
WT_1a.d	WT_1a	Sample	P1-A7	Per method		NCF_LCMS_neg_IDH.m
G98_1a.d	G98_1a	Sample	P1-A8	Per method		NCF_LCMS_neg_IDH.m
R133_1.d	R133_1	Sample	P1-A9	Per method		NCF_LCMS_neg_IDH.m
G98_1a_slow.d	G98_1a	Sample	P1-A8	Per method		NCF_LCMS_neg_IDH.m
WT_1a_2nd_run.d	WT_1a_2	Sample	P1-A1	Per method		NCF_LCMS_neg_IDH.m
WT_1b.d	WT_1b	Sample	P1-A4	Per method		NCF_LCMS_neg_IDH.m
WT_2.d	WT_2	Sample	P1-A5	Per method		NCF_LCMS_neg_IDH.m
G98_1b.d	G98_1b	Sample	P1-A8	Per method		NCF_LCMS_neg_IDH.m
G98_2.d	G98_2	Sample	P1-B1	Per method		NCF_LCMS_neg_IDH.m
R133_1_2nd_run.d	R133_1	Sample	P1-B4	Per method		NCF_LCMS_neg_IDH.m
R133_2.d	R133_2	Sample	P1-B5	Per method		NCF_LCMS_neg_IDH.m
R133_3.d	R133_3	Sample	P1-B6	Per method		NCF_LCMS_neg_IDH.m
rxnmix_div_2.d	rxn_mix_div_2	Sample	P1-B8	Per method		NCF_LCMS_neg_IDH.m

Quantitation Results

Target Compound *2-HG*

Quantitative Analysis Summary Report

Data File	Compound	ISTD	Sample Type	Response	ISTD Resp	Resp Ratio	Conc.	Exp Conc	Accuracy
2HG_200uM.d	2-HG		Sample	413003			165.84		
2HG_200uM_2.d	2-HG		Sample	459586			184.41		
2HG_0_1uM.d	2-HG		Calibration	304			1.35	0.10	1347.31
2HG_0_5uM.d	2-HG		Calibration	1460			1.81	0.50	361.63
2HG_1uM.d	2-HG		Calibration	2055			2.05	1.00	204.51
2HG_2uM.d	2-HG		Calibration	3874			2.77	2.00	138.51
2HG_5uM.d	2-HG		Calibration	9577			5.04	5.00	100.87
2HG_12uM.d	2-HG		Calibration	23403			10.55	12.00	87.95
2HG_25uM.d	2-HG		Calibration	53829			22.68	25.00	90.73
2HG_50uM.d	2-HG		Calibration	115507			47.27	50.00	94.53
2HG_100uM.d	2-HG		Calibration	253045			102.08	100.00	102.08
blank13_50mM_no_wash.d	2-HG		Blank	30			1.24		
blank15_50mM_no_wash.d	2-HG		Blank	19			1.23		
blank17_50mM_no_wash.d	2-HG		Blank	141			1.28		
blank18_50mM_no_wash.d	2-HG		Blank	241			1.32		
WT_1a.d	2-HG		Sample	3386			2.58		
G98_1a.d	2-HG		Sample	23887			10.75		
R133_1.d	2-HG		Sample	157553			64.02		
G98_1a_slow.d	2-HG		Sample	26474			11.78		
WT_1a_2nd_run.d	2-HG		Sample	3849			2.76		
WT_1b.d	2-HG		Sample	3232			2.51		
WT_2.d	2-HG		Sample	3837			2.76		
G98_1b.d	2-HG		Sample	26613			11.83		
G98_2.d	2-HG		Sample	23532			10.61		
R133_1_2nd_run.d	2-HG		Sample	173856			70.52		
R133_2.d	2-HG		Sample	157663			64.07		
R133_3.d	2-HG		Sample	174466			70.76		
rxnmix_div_2.d	2-HG		Sample	43			1.24		

Quantitative Analysis Calibration Report

**Batch Results**	D:\MassHunter\Data\Users\062023\Walstrom\LCMS6_13\QuantResults\2-HG_6_13analysis_batch.bat		
**Analysis Time**	6/14/2023 11:06 AM	**Analyst Name**	admin
**Report Time**	6/14/2023 11:13 AM	**Reporter Name**	admin
**Last Calib Update**	6/14/2023 11:06 AM	**Batch State**	Processed

Calibration Info

Target Compound *2-HG*

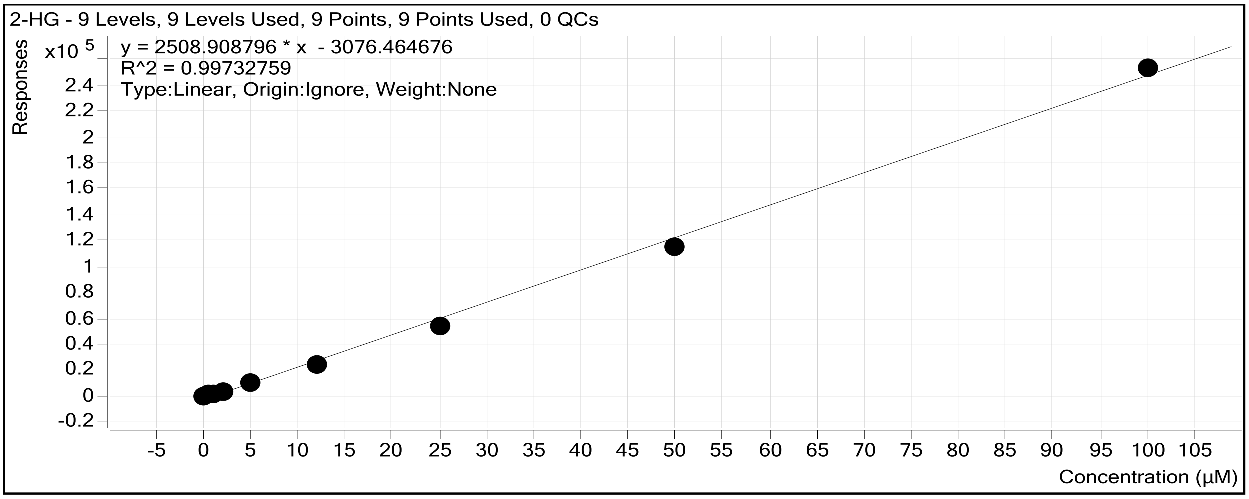

Calibration STD	Cal Type	Level	Enabled	Response	Exp Conc	RF
D:\MassHunter\Data\Users\062023\Walstrom\LCMS6_13\2HG_0_1uM.d	Calibration	1	☑	304	0.1000	3038.0974
D:\MassHunter\Data\Users\062023\Walstrom\LCMS6_13\2HG_0_5uM.d	Calibration	2	☑	1460	0.5000	2919.9895
D:\MassHunter\Data\Users\062023\Walstrom\LCMS6_13\2HG_1uM.d	Calibration	3	☑	2055	1.0000	2054.5257
D:\MassHunter\Data\Users\062023\Walstrom\LCMS6_13\2HG_2uM.d	Calibration	4	☑	3874	2.0000	1936.9069
D:\MassHunter\Data\Users\062023\Walstrom\LCMS6_13\2HG_5uM.d	Calibration	5	☑	9577	5.0000	1915.4470
D:\MassHunter\Data\Users\062023\Walstrom\LCMS6_13\2HG_12uM.d	Calibration	6	☑	23403	12.0000	1950.2864
D:\MassHunter\Data\Users\062023\Walstrom\LCMS6_13\2HG_25uM.d	Calibration	7	☑	53829	25.0000	2153.1729
D:\MassHunter\Data\Users\062023\Walstrom\LCMS6_13\2HG_50uM.d	Calibration	8	☑	115507	50.0000	2310.1492
D:\MassHunter\Data\Users\062023\Walstrom\LCMS6_13\2HG_100uM.d	Calibration	9	☑	253045	100.0000	2530.4478

Analysis of wild-type IDH-1 2HG production

This standard curve includes the lower concentration standards, including the blanks.

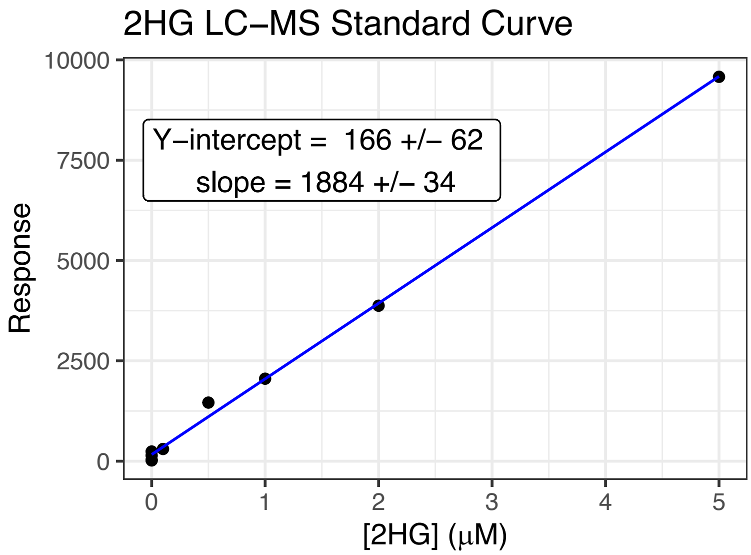

Results

This standard curve was used to calculate the 2HG concentrations for the wild-type samples.

Sample	[2HG]
WT_1a_2nd_run	1.95
WT_1b	1.63
WT_2	1.95

The mean is 1.84 µM with a standard error of 0.11 µM.

Quantitative Analysis Sample Report

**Batch Results**	D:\MassHunter\Data\Users\062023\Walstrom\LCMS6_13\QuantResults\2-HG_6_13analysis_batch.batch.bin		
**Analysis Time**	6/14/2023 11:06 AM	**Analyst Name**	admin
**Report Time**	6/14/2023 11:13 AM	**Reporter Name**	admin
**Last Calib Update**	6/14/2023 11:06 AM	**Batch State**	Processed

Analysis Info

**Acq Time**	2023-06-13 11:33	**Data File**	2HG_200uM.d
**Position**	P1-A1	**Sample Name**	2HG_200
**Dilution**	1	**Sample Info**	
**Inj. Volume**	Per method	**Acq Method File**	NCF_LCMS_neg_IDH.m
**Sample Type**	Sample	**Comment**	

Sample Chromatogram

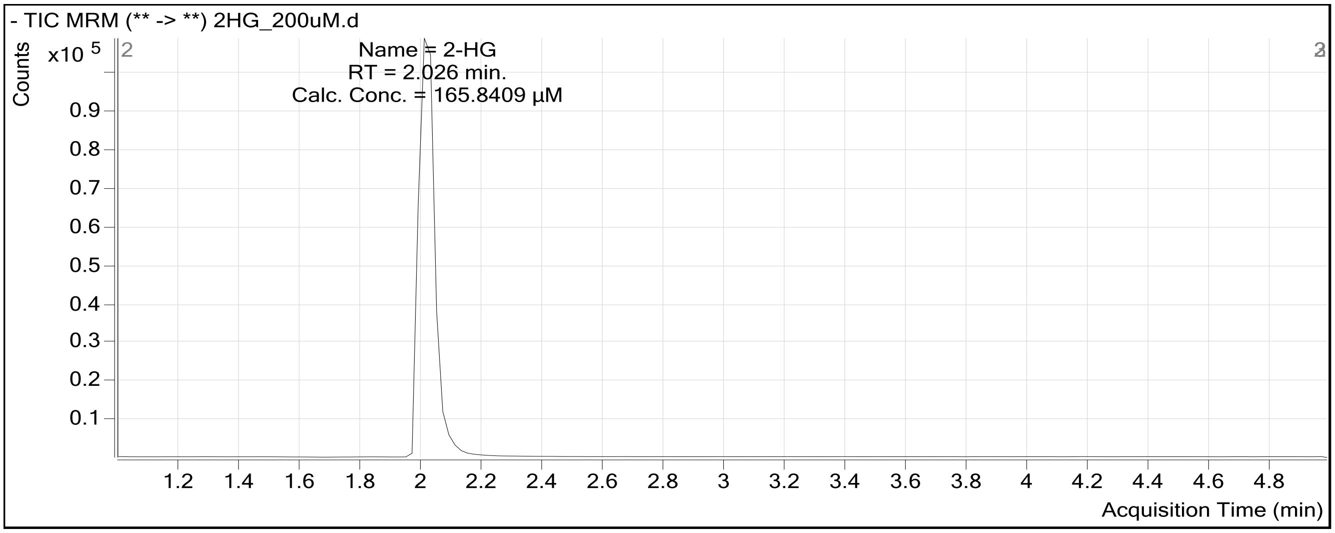

Quantitation Results

Compound	ISTD	RT	Response	ISTD Resp	RR	Conc.	Accuracy
2-HG		2.026	413003			165.84	

Compound Graphics

Target Compound *2-HG*

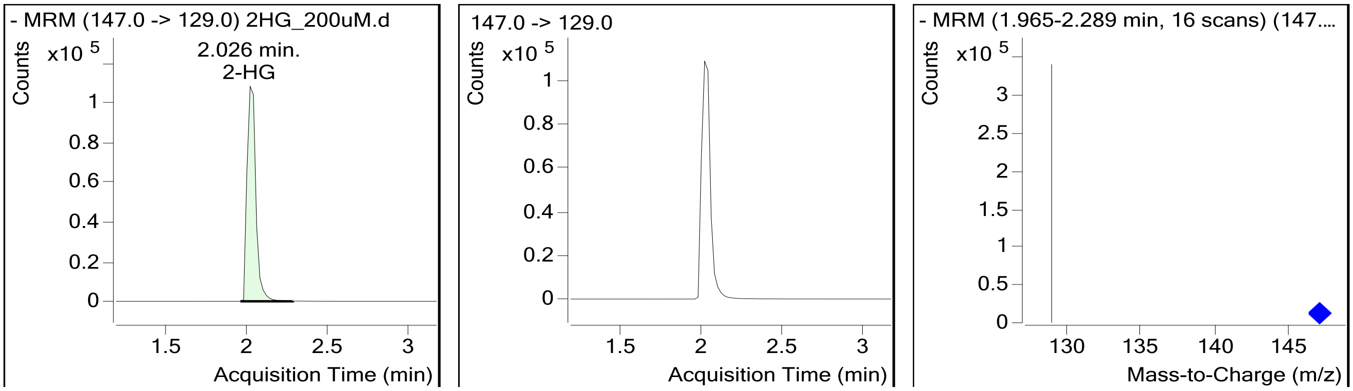

Quantitative Analysis Sample Report

**Batch Results**	D:\MassHunter\Data\Users\062023\Walstrom\LCMS6_13\QuantResults\2-HG_6_13analysis_batch.batch.bin		
**Analysis Time**	6/14/2023 11:06 AM	**Analyst Name**	admin
**Report Time**	6/14/2023 11:13 AM	**Reporter Name**	admin
**Last Calib Update**	6/14/2023 11:06 AM	**Batch State**	Processed

Analysis Info

**Acq Time**	2023-06-13 12:06	**Data File**	2HG_200uM_2.d
**Position**	P1-A1	**Sample Name**	2HG_200
**Dilution**	1	**Sample Info**	
**Inj. Volume**	Per method	**Acq Method File**	NCF_LCMS_neg_IDH.m
**Sample Type**	Sample	**Comment**	

Sample Chromatogram

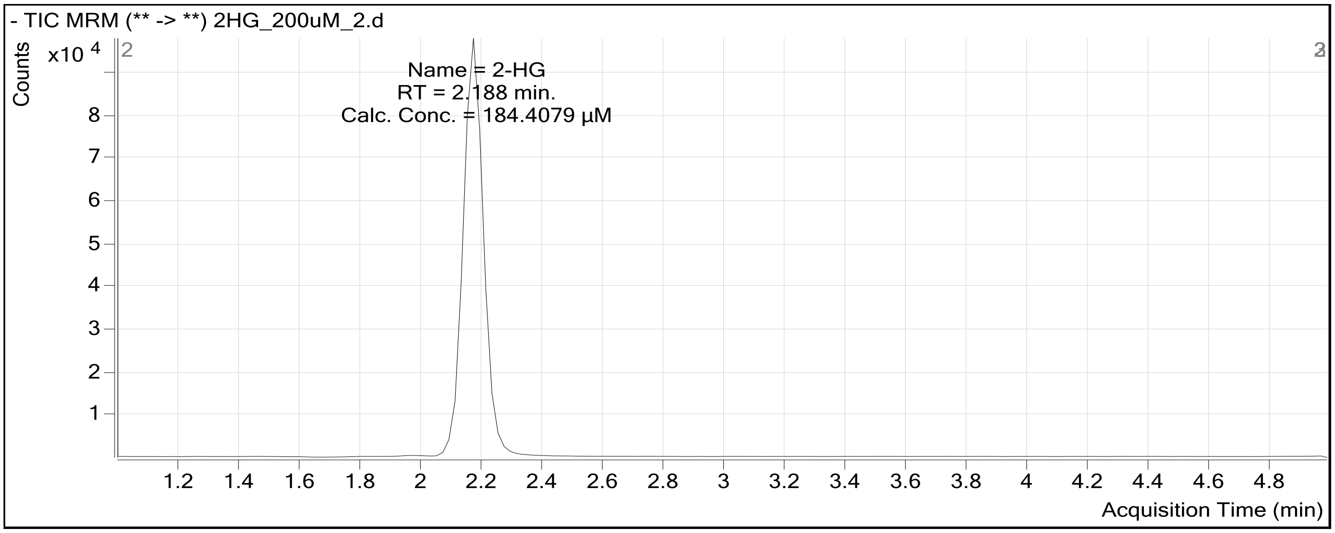

Quantitation Results

Compound	ISTD	RT	Response	ISTD Resp	RR	Conc.	Accuracy
2-HG		2.188	459586			184.41	

Compound Graphics

Target Compound *2-HG*

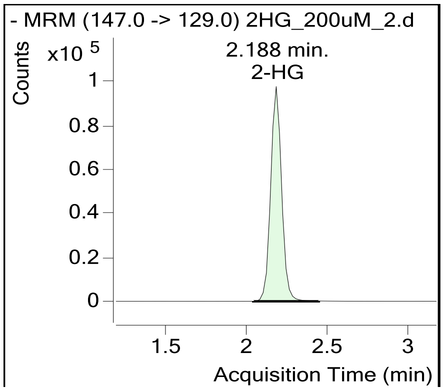

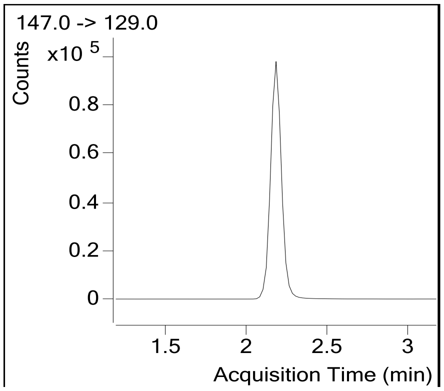

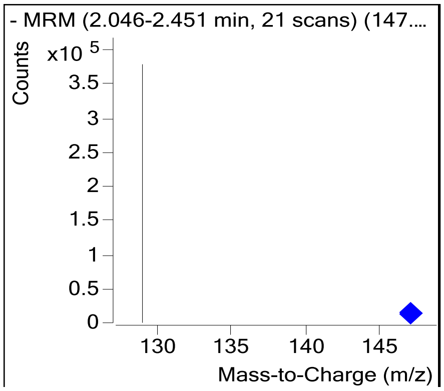

Quantitative Analysis Sample Report

**Batch Results**	D:\MassHunter\Data\Users\062023\Walstrom\LCMS6_13\QuantResults\2-HG_6_13analysis_batch.batch.bin		
**Analysis Time**	6/14/2023 11:06 AM	**Analyst Name**	admin
**Report Time**	6/14/2023 11:13 AM	**Reporter Name**	admin
**Last Calib Update**	6/14/2023 11:06 AM	**Batch State**	Processed

Analysis Info

**Acq Time**	2023-06-13 20:51	**Data File**	2HG_0_1uM.d
**Position**	P1-F3	**Sample Name**	2HG_0_1
**Dilution**	1	**Sample Info**	
**Inj. Volume**	Per method	**Acq Method File**	NCF_LCMS_neg_IDH.m
**Sample Type**	Calibration	**Comment**	

Sample Chromatogram

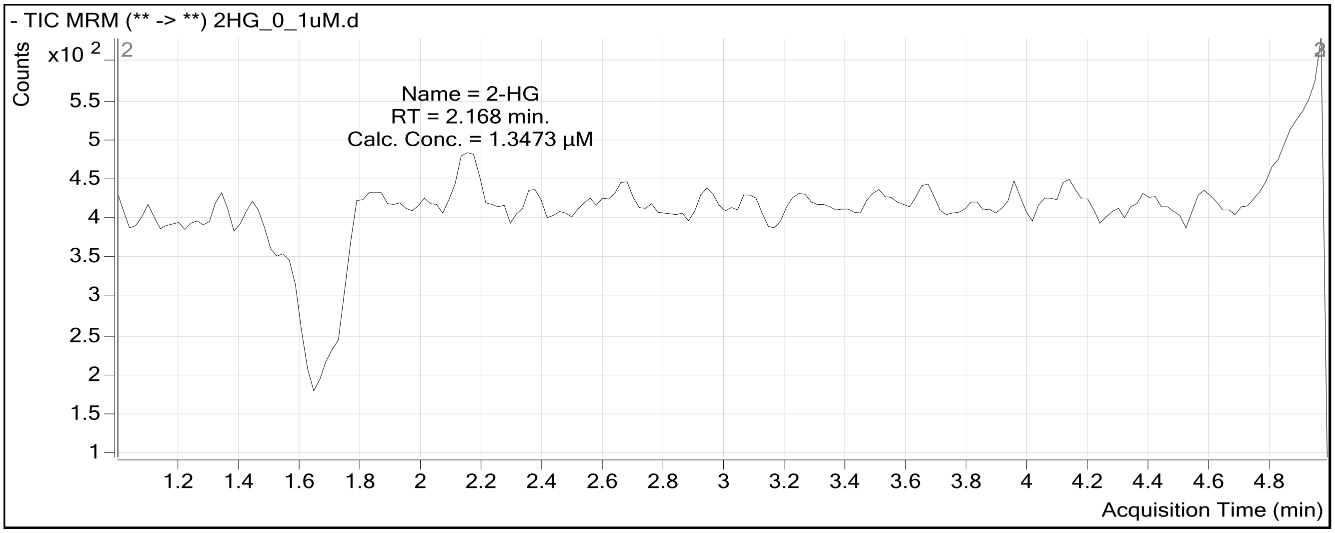

Quantitation Results

Compound	ISTD	RT	Response	ISTD Resp	RR	Conc.	Accuracy
2-HG		2.168	304			1.35	1347.31

Compound Graphics

Target Compound *2-HG*

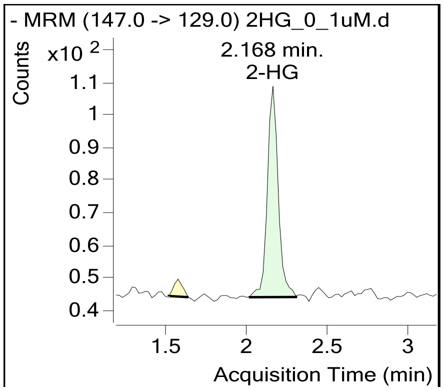

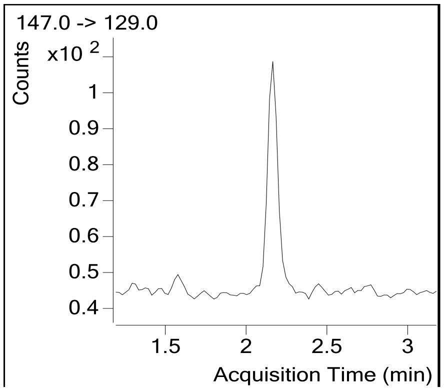

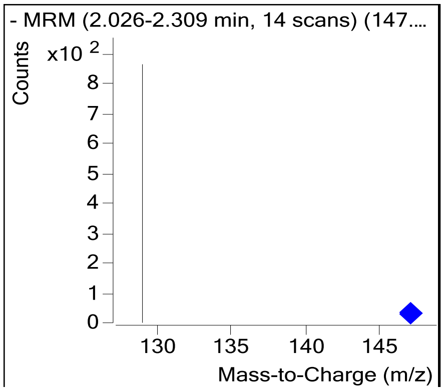

Quantitative Analysis Sample Report

**Batch Results**	D:\MassHunter\Data\Users\062023\Walstrom\LCMS6_13\QuantResults\2-HG_6_13analysis_batch.batch.bin		
**Analysis Time**	6/14/2023 11:06 AM	**Analyst Name**	admin
**Report Time**	6/14/2023 11:13 AM	**Reporter Name**	admin
**Last Calib Update**	6/14/2023 11:06 AM	**Batch State**	Processed

Analysis Info

**Acq Time**	2023-06-13 21:02	**Data File**	2HG_0_5uM.d
**Position**	P1-F4	**Sample Name**	2HG_0_5
**Dilution**	1	**Sample Info**	
**Inj. Volume**	Per method	**Acq Method File**	NCF_LCMS_neg_IDH.m
**Sample Type**	Calibration	**Comment**	

Sample Chromatogram

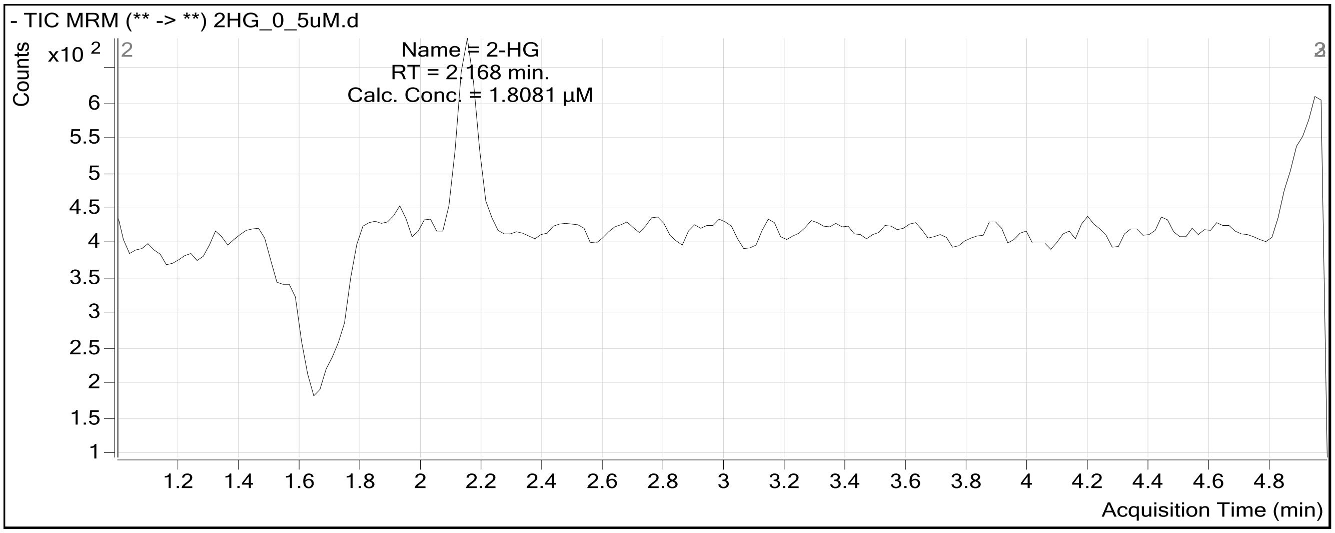

Quantitation Results

Compound	ISTD	RT	Response	ISTD Resp	RR	Conc.	Accuracy
2-HG		2.168	1460			1.81	361.63

Compound Graphics

Target Compound *2-HG*

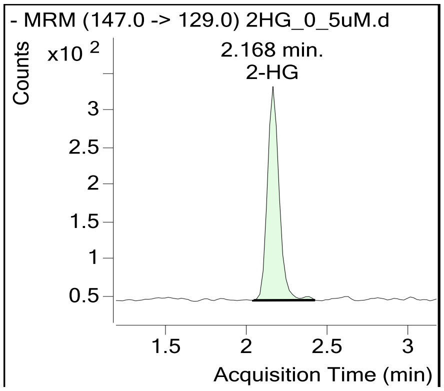

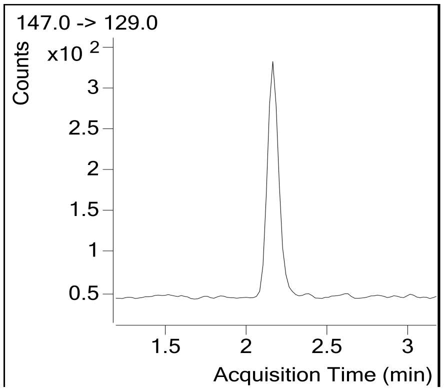

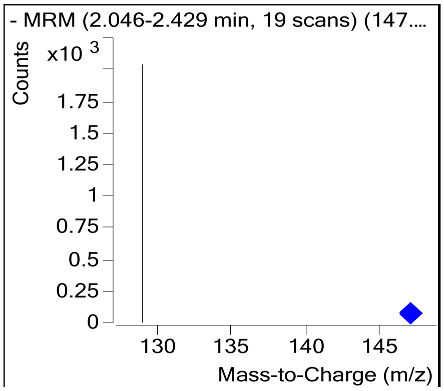

Quantitative Analysis Sample Report

**Batch Results**	D:\MassHunter\Data\Users\062023\Walstrom\LCMS6_13\QuantResults\2-HG_6_13analysis_batch.batch.bin		
**Analysis Time**	6/14/2023 11:06 AM	**Analyst Name**	admin
**Report Time**	6/14/2023 11:13 AM	**Reporter Name**	admin
**Last Calib Update**	6/14/2023 11:06 AM	**Batch State**	Processed

Analysis Info

**Acq Time**	2023-06-13 21:13	**Data File**	2HG_1uM.d
**Position**	P1-C1	**Sample Name**	2HG_1
**Dilution**	1	**Sample Info**	
**Inj. Volume**	Per method	**Acq Method File**	NCF_LCMS_neg_IDH.m
**Sample Type**	Calibration	**Comment**	

Sample Chromatogram

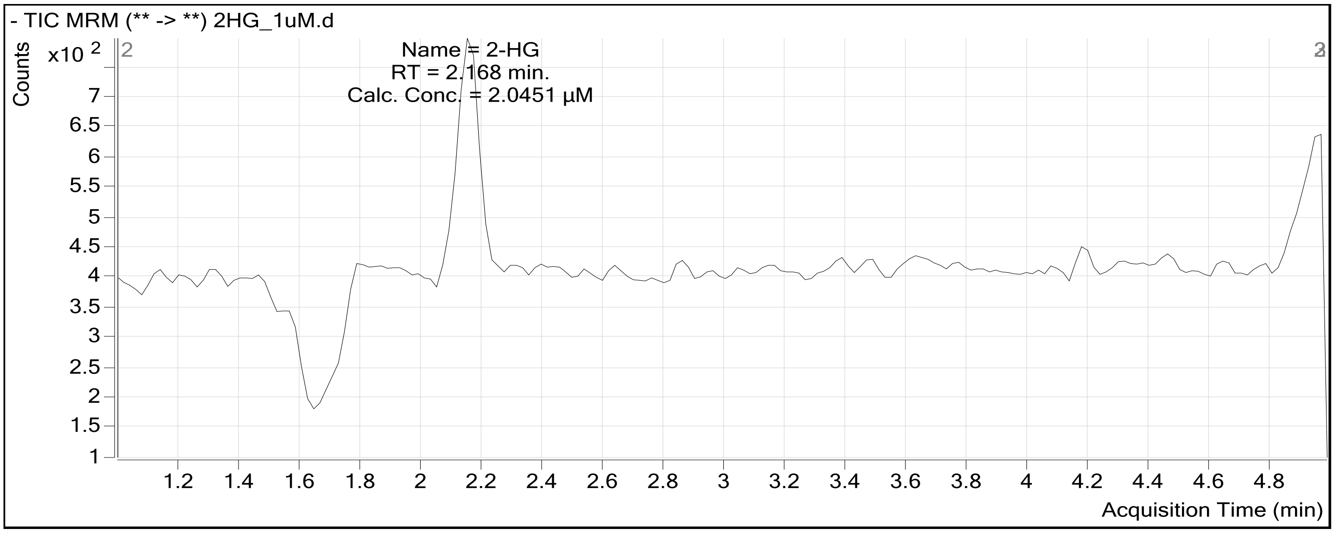

Quantitation Results

Compound	ISTD	RT	Response	ISTD Resp	RR	Conc.	Accuracy
2-HG		2.168	2055			2.05	204.51

Compound Graphics

Target Compound *2-HG*

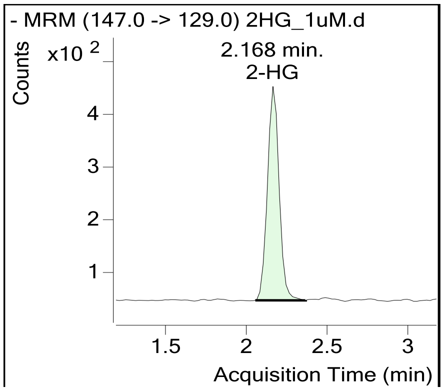

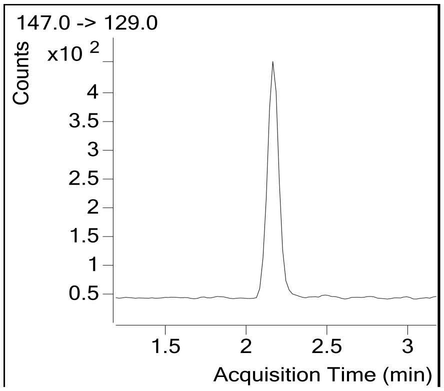

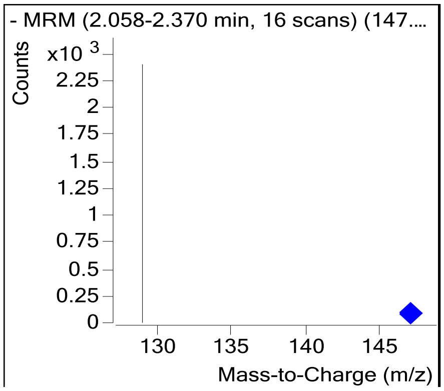

Quantitative Analysis Sample Report

**Batch Results**	D:\MassHunter\Data\Users\062023\Walstrom\LCMS6_13\QuantResults\2-HG_6_13analysis_batch.batch.bin		
**Analysis Time**	6/14/2023 11:06 AM	**Analyst Name**	admin
**Report Time**	6/14/2023 11:13 AM	**Reporter Name**	admin
**Last Calib Update**	6/14/2023 11:06 AM	**Batch State**	Processed

Analysis Info

**Acq Time**	2023-06-13 21:23	**Data File**	2HG_2uM.d
**Position**	P1-C2	**Sample Name**	2HG_2
**Dilution**	1	**Sample Info**	
**Inj. Volume**	Per method	**Acq Method File**	NCF_LCMS_neg_IDH.m
**Sample Type**	Calibration	**Comment**	

Sample Chromatogram

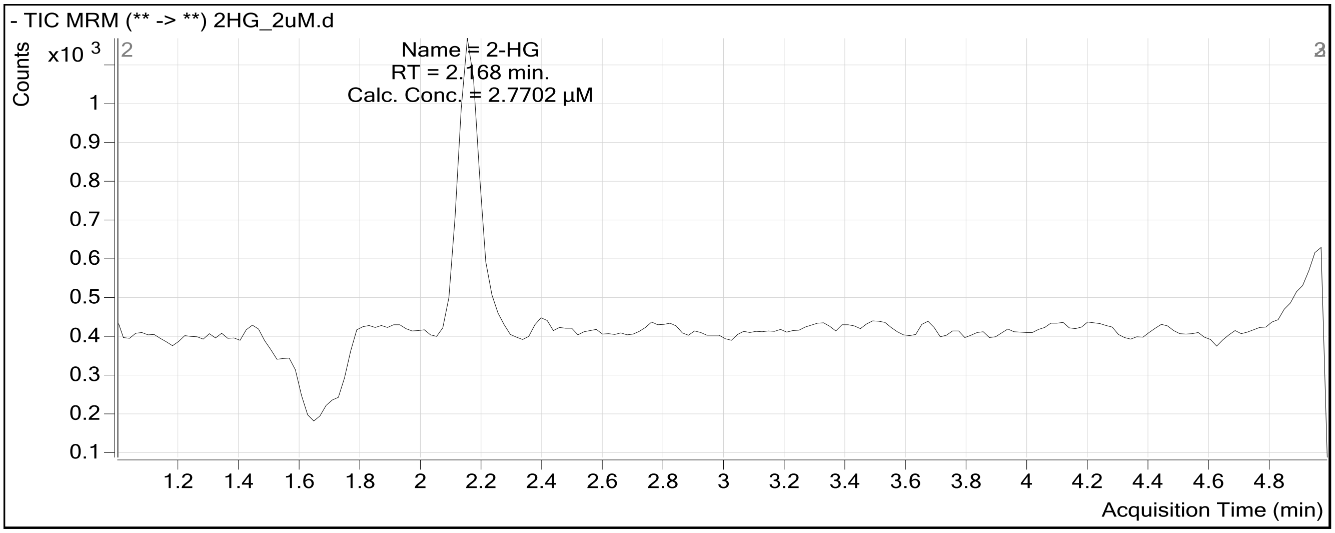

Quantitation Results

Compound	ISTD	RT	Response	ISTD Resp	RR	Conc.	Accuracy
2-HG		2.168	3874			2.77	138.51

Compound Graphics

Target Compound *2-HG*

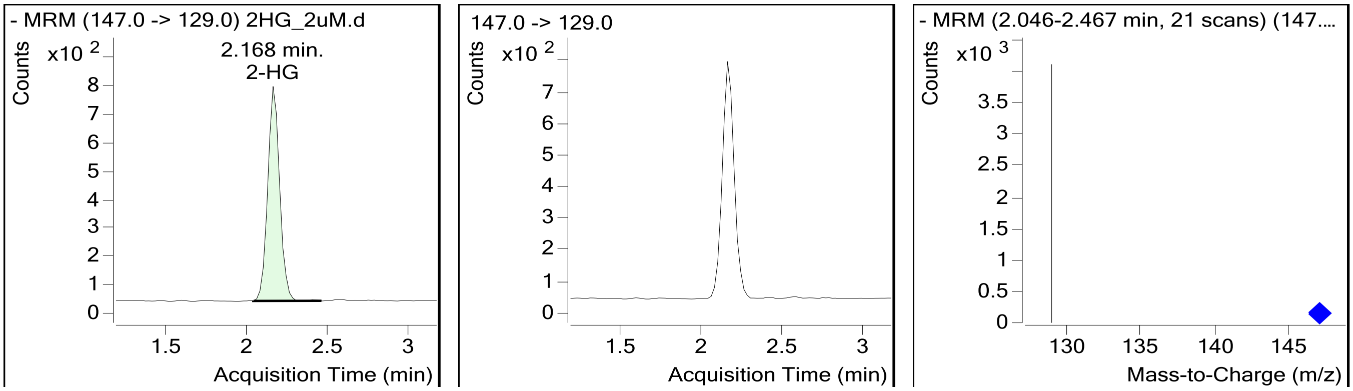

Quantitative Analysis Sample Report

**Batch Results**	D:\MassHunter\Data\Users\062023\Walstrom\LCMS6_13\QuantResults\2-HG_6_13analysis_batch.batch.bin		
**Analysis Time**	6/14/2023 11:06 AM	**Analyst Name**	admin
**Report Time**	6/14/2023 11:13 AM	**Reporter Name**	admin
**Last Calib Update**	6/14/2023 11:06 AM	**Batch State**	Processed

Analysis Info

**Acq Time**	2023-06-13 21:34	**Data File**	2HG_5uM.d
**Position**	P1-C3	**Sample Name**	2HG_5
**Dilution**	1	**Sample Info**	
**Inj. Volume**	Per method	**Acq Method File**	NCF_LCMS_neg_IDH.m
**Sample Type**	Calibration	**Comment**	

Sample Chromatogram

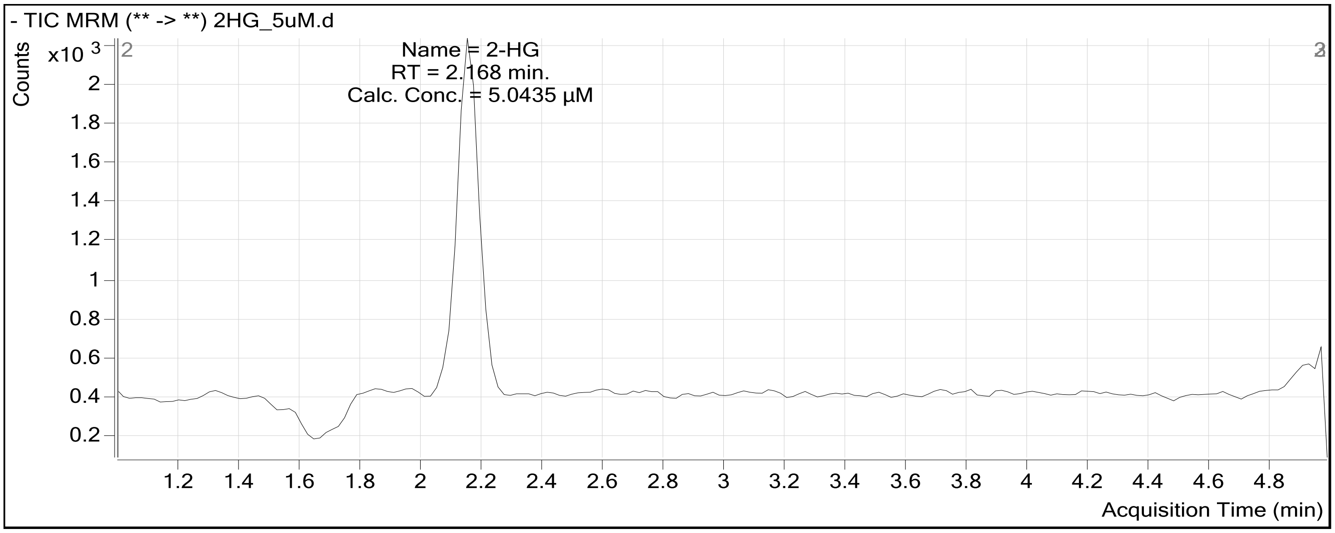

Quantitation Results

Compound	ISTD	RT	Response	ISTD Resp	RR	Conc.	Accuracy
2-HG		2.168	9577			5.04	100.87

Compound Graphics

Target Compound *2-HG*

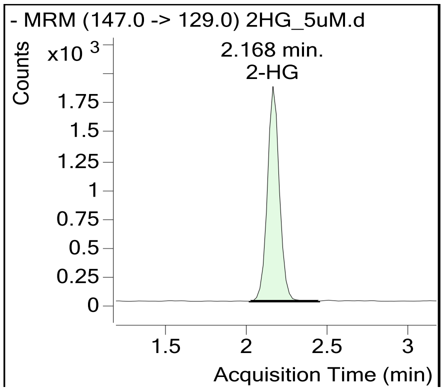

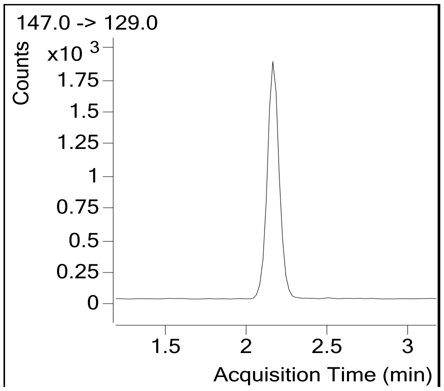

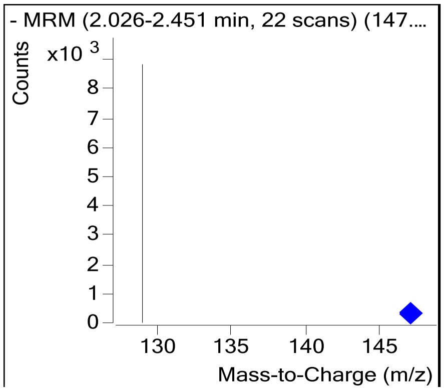

Quantitative Analysis Sample Report

**Batch Results**	D:\MassHunter\Data\Users\062023\Walstrom\LCMS6_13\QuantResults\2-HG_6_13analysis_batch.batch.bin		
**Analysis Time**	6/14/2023 11:06 AM	**Analyst Name**	admin
**Report Time**	6/14/2023 11:13 AM	**Reporter Name**	admin
**Last Calib Update**	6/14/2023 11:06 AM	**Batch State**	Processed

Analysis Info

**Acq Time**	2023-06-13 21:45	**Data File**	2HG_12uM.d
**Position**	P1-C4	**Sample Name**	2HG_12
**Dilution**	1	**Sample Info**	
**Inj. Volume**	Per method	**Acq Method File**	NCF_LCMS_neg_IDH.m
**Sample Type**	Calibration	**Comment**	

Sample Chromatogram

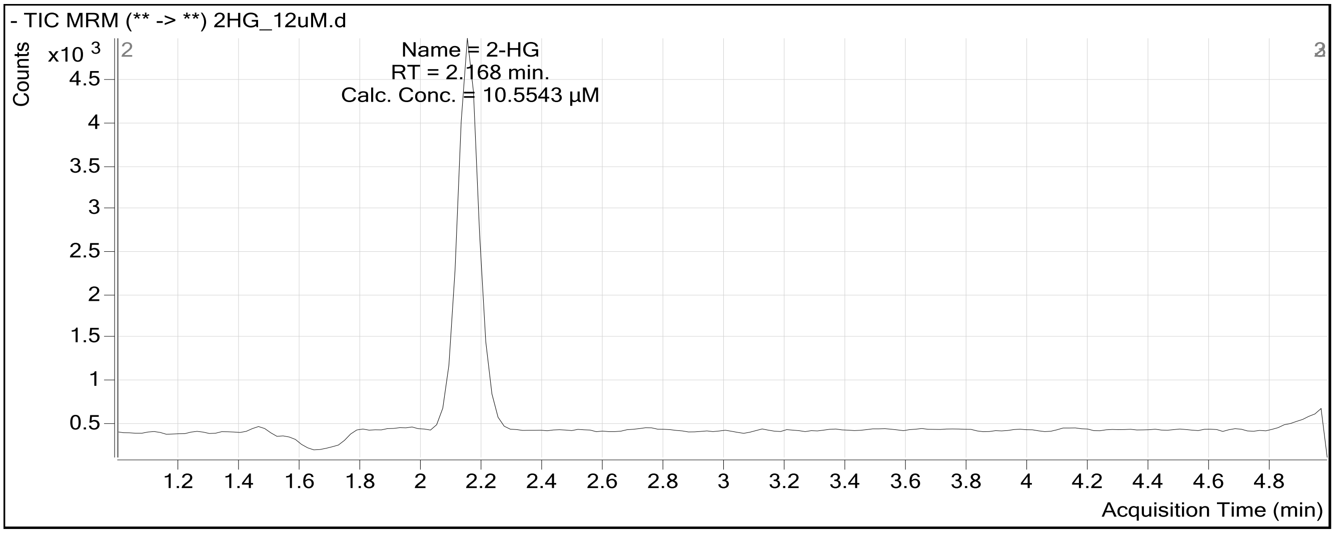

Quantitation Results

Compound	ISTD	RT	Response	ISTD Resp	RR	Conc.	Accuracy
2-HG		2.168	23403			10.55	87.95

Compound Graphics

Target Compound *2-HG*

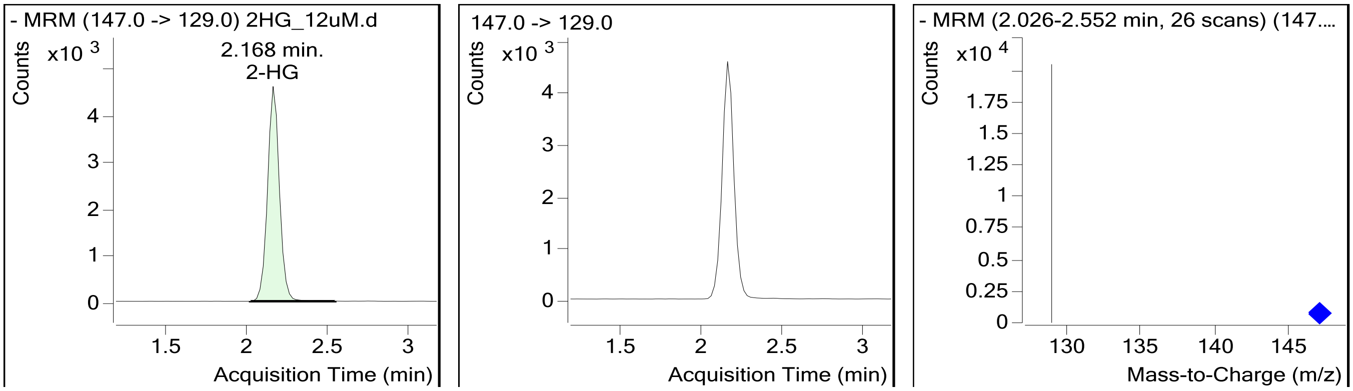

Quantitative Analysis Sample Report

**Batch Results**	D:\MassHunter\Data\Users\062023\Walstrom\LCMS6_13\QuantResults\2-HG_6_13analysis_batch.batch.bin		
**Analysis Time**	6/14/2023 11:06 AM	**Analyst Name**	admin
**Report Time**	6/14/2023 11:13 AM	**Reporter Name**	admin
**Last Calib Update**	6/14/2023 11:06 AM	**Batch State**	Processed

Analysis Info

**Acq Time**	2023-06-13 21:56	**Data File**	2HG_25uM.d
**Position**	P1-C5	**Sample Name**	2HG_25
**Dilution**	1	**Sample Info**	
**Inj. Volume**	Per method	**Acq Method File**	NCF_LCMS_neg_IDH.m
**Sample Type**	Calibration	**Comment**	

Sample Chromatogram

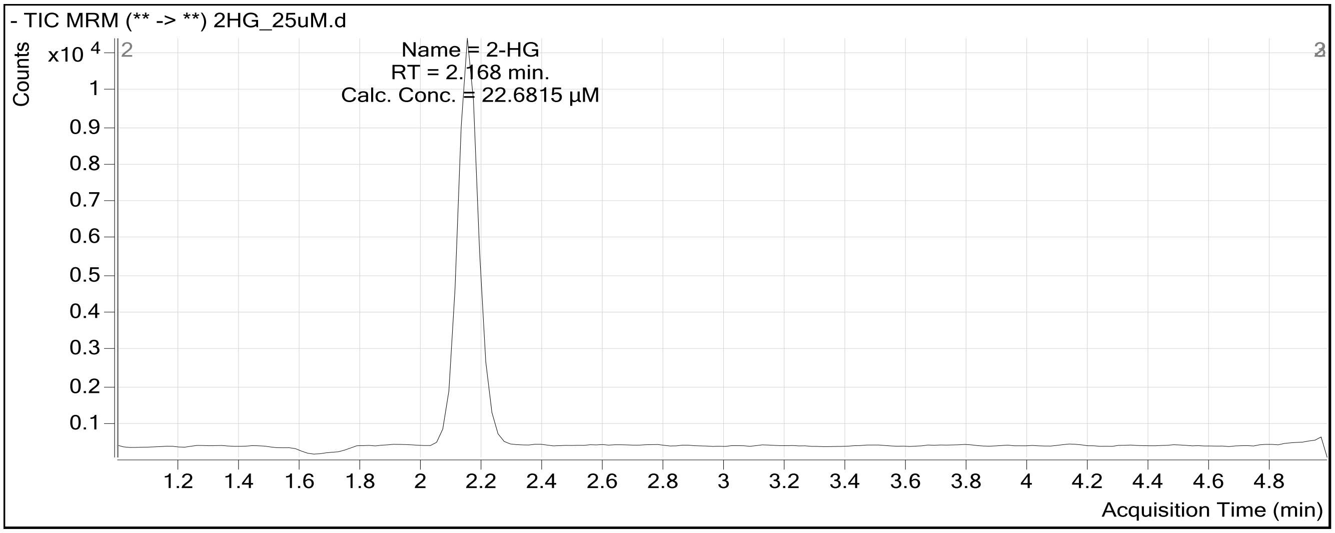

Quantitation Results

Compound	ISTD	RT	Response	ISTD Resp	RR	Conc.	Accuracy
2-HG		2.168	53829			22.68	90.73

Compound Graphics

Target Compound *2-HG*

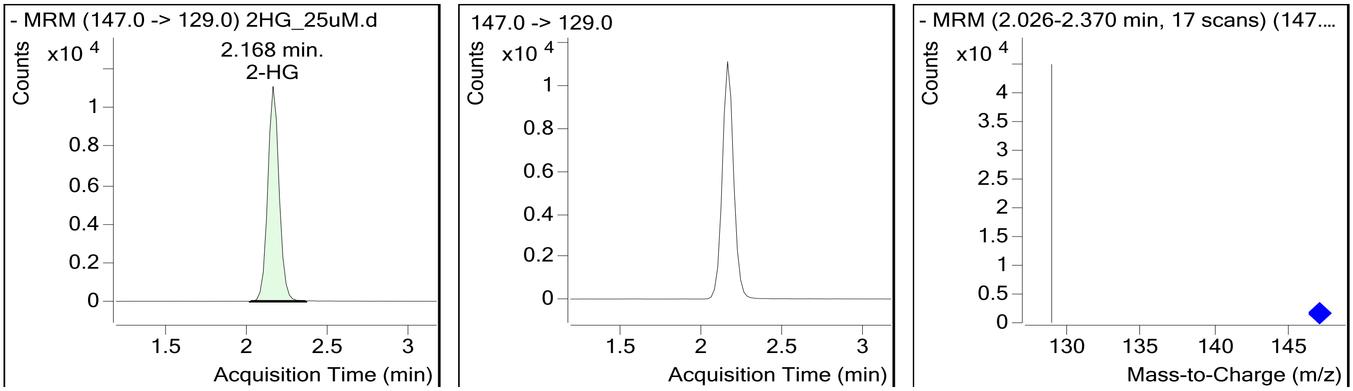

Quantitative Analysis Sample Report

**Batch Results**	D:\MassHunter\Data\Users\062023\Walstrom\LCMS6_13\QuantResults\2-HG_6_13analysis_batch.batch.bin		
**Analysis Time**	6/14/2023 11:06 AM	**Analyst Name**	admin
**Report Time**	6/14/2023 11:13 AM	**Reporter Name**	admin
**Last Calib Update**	6/14/2023 11:06 AM	**Batch State**	Processed

Analysis Info

**Acq Time**	2023-06-13 22:07	**Data File**	2HG_50uM.d
**Position**	P1-C6	**Sample Name**	2HG_50
**Dilution**	1	**Sample Info**	
**Inj. Volume**	Per method	**Acq Method File**	NCF_LCMS_neg_IDH.m
**Sample Type**	Calibration	**Comment**	

Sample Chromatogram

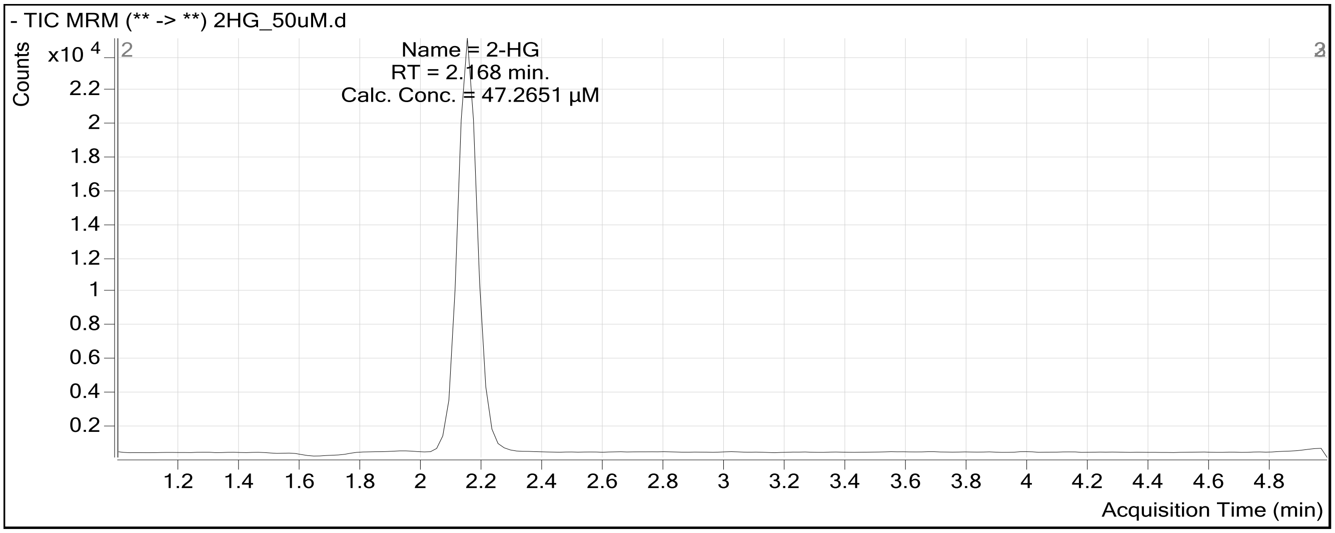

Quantitation Results

Compound	ISTD	RT	Response	ISTD Resp	RR	Conc.	Accuracy
2-HG		2.168	115507			47.27	94.53

Compound Graphics

Target Compound *2-HG*

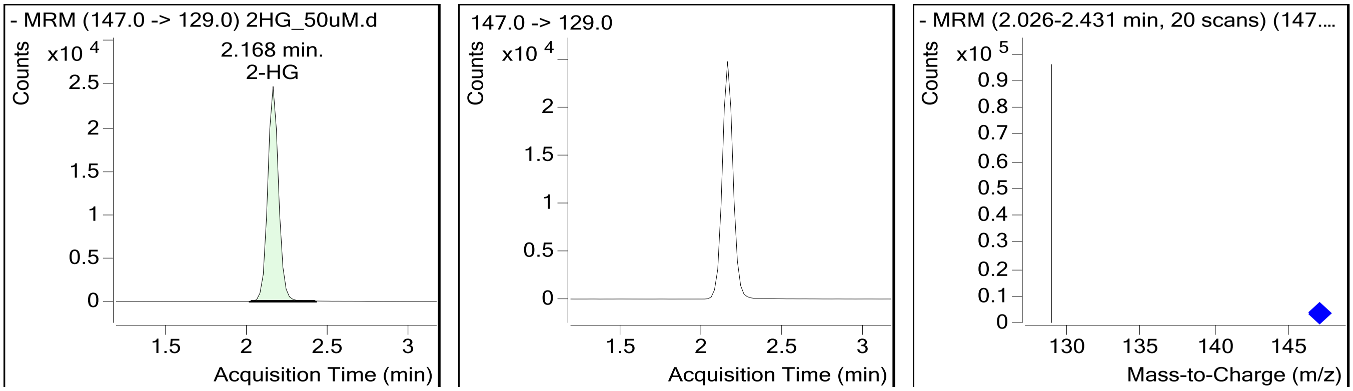

Quantitative Analysis Sample Report

**Batch Results**	D:\MassHunter\Data\Users\062023\Walstrom\LCMS6_13\QuantResults\2-HG_6_13analysis_batch.batch.bin		
**Analysis Time**	6/14/2023 11:06 AM	**Analyst Name**	admin
**Report Time**	6/14/2023 11:13 AM	**Reporter Name**	admin
**Last Calib Update**	6/14/2023 11:06 AM	**Batch State**	Processed

Analysis Info

**Acq Time**	2023-06-13 22:18	**Data File**	2HG_100uM.d
**Position**	P1-C7	**Sample Name**	2HG_100
**Dilution**	1	**Sample Info**	
**Inj. Volume**	Per method	**Acq Method File**	NCF_LCMS_neg_IDH.m
**Sample Type**	Calibration	**Comment**	

Sample Chromatogram

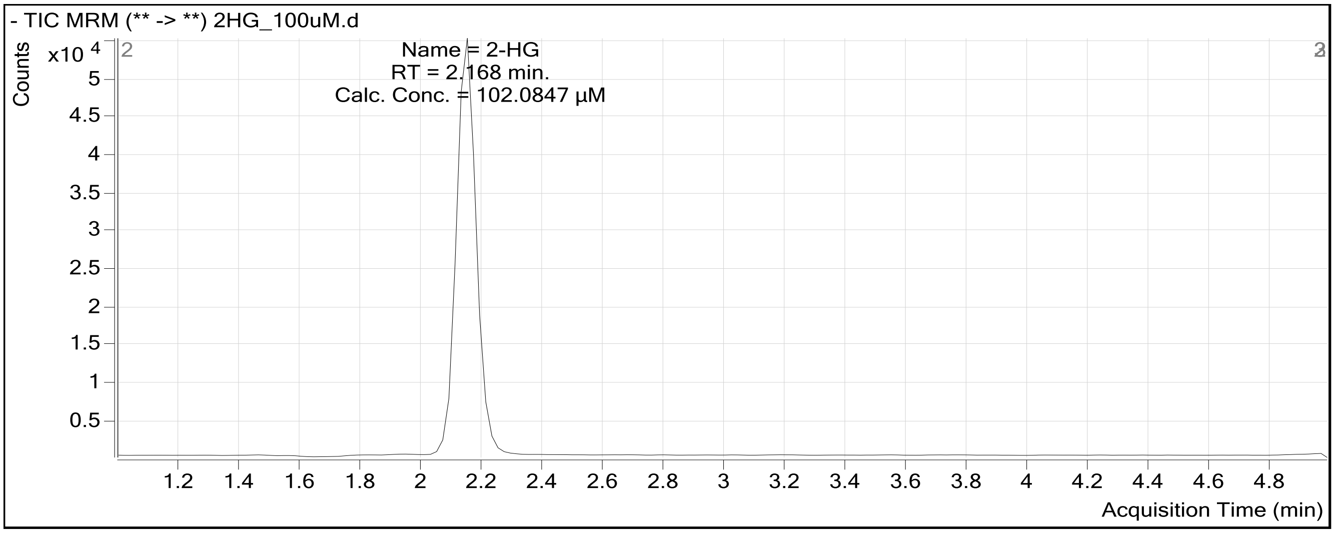

Quantitation Results

Compound	ISTD	RT	Response	ISTD Resp	RR	Conc.	Accuracy
2-HG		2.168	253045			102.08	102.08

Compound Graphics

Target Compound *2-HG*

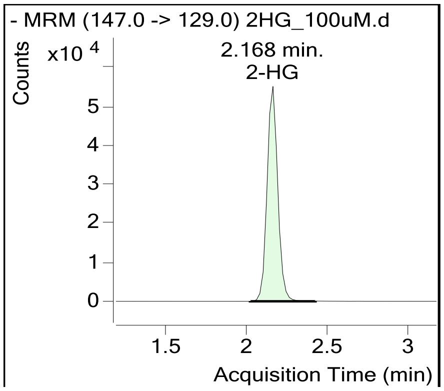

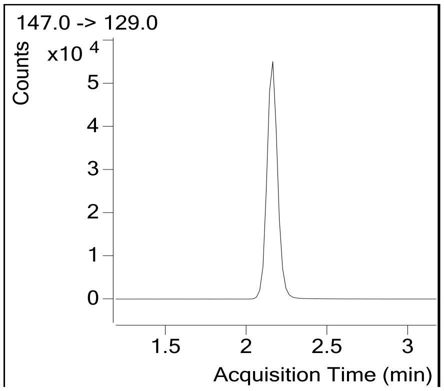

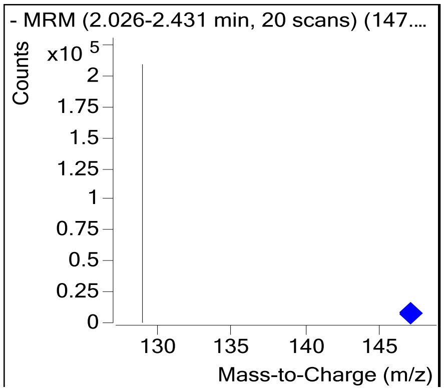

Quantitative Analysis Sample Report

**Batch Results**	D:\MassHunter\Data\Users\062023\Walstrom\LCMS6_13\QuantResults\2-HG_6_13analysis_batch.batch.bin		
**Analysis Time**	6/14/2023 11:06 AM	**Analyst Name**	admin
**Report Time**	6/14/2023 11:13 AM	**Reporter Name**	admin
**Last Calib Update**	6/14/2023 11:06 AM	**Batch State**	Processed

Analysis Info

**Acq Time**	2023-06-13 17:46	**Data File**	blank13_50mM_no_wash.d
**Position**	P1-A3	**Sample Name**	blank_50mM_13
**Dilution**	1	**Sample Info**	
**Inj. Volume**	Per method	**Acq Method File**	NCF_LCMS_neg_IDH.m
**Sample Type**	Blank	**Comment**	

Sample Chromatogram

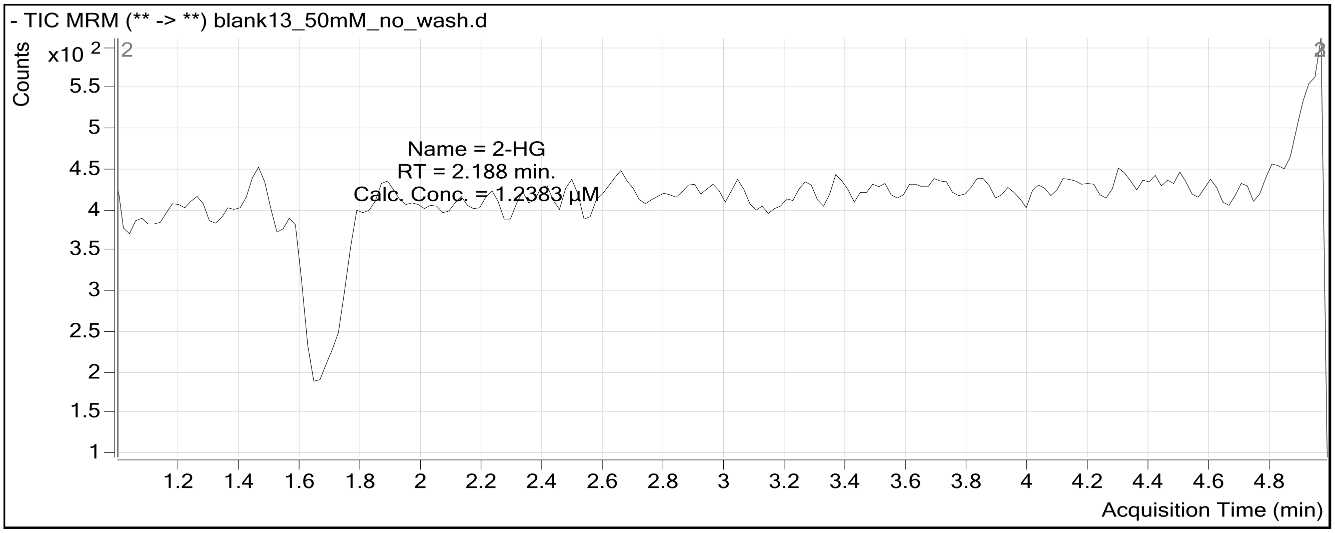

Quantitation Results

Compound	ISTD	RT	Response	ISTD Resp	RR	Conc.	Accuracy
2-HG		2.188	30			1.24	

Compound Graphics

Target Compound *2-HG*

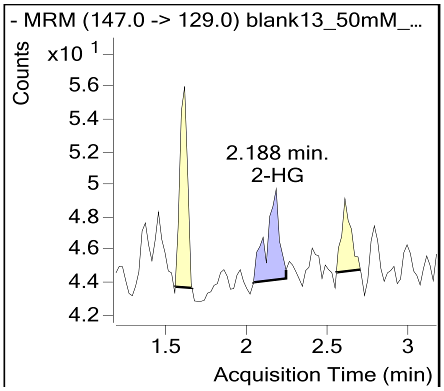

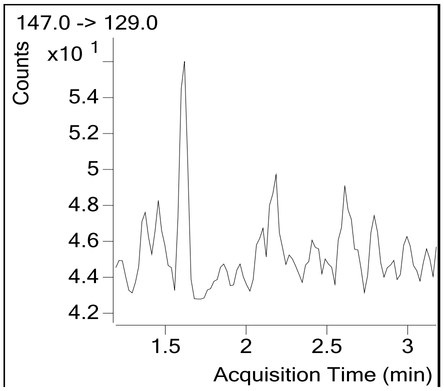

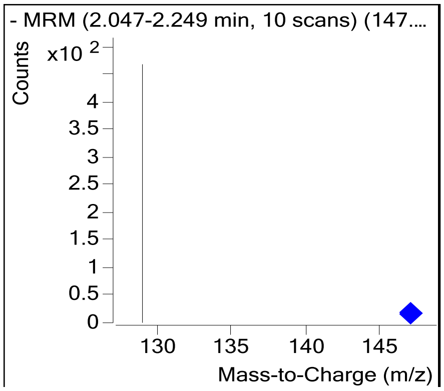

Quantitative Analysis Sample Report

**Batch Results**	D:\MassHunter\Data\Users\062023\Walstrom\LCMS6_13\QuantResults\2-HG_6_13analysis_batch.batch.bin		
**Analysis Time**	6/14/2023 11:06 AM	**Analyst Name**	admin
**Report Time**	6/14/2023 11:13 AM	**Reporter Name**	admin
**Last Calib Update**	6/14/2023 11:06 AM	**Batch State**	Processed

Analysis Info

**Acq Time**	2023-06-13 18:40	**Data File**	blank15_50mM_no_wash.d
**Position**	P1-A7	**Sample Name**	blank_50mM_15
**Dilution**	1	**Sample Info**	
**Inj. Volume**	Per method	**Acq Method File**	NCF_LCMS_neg_IDH.m
**Sample Type**	Blank	**Comment**	

Sample Chromatogram

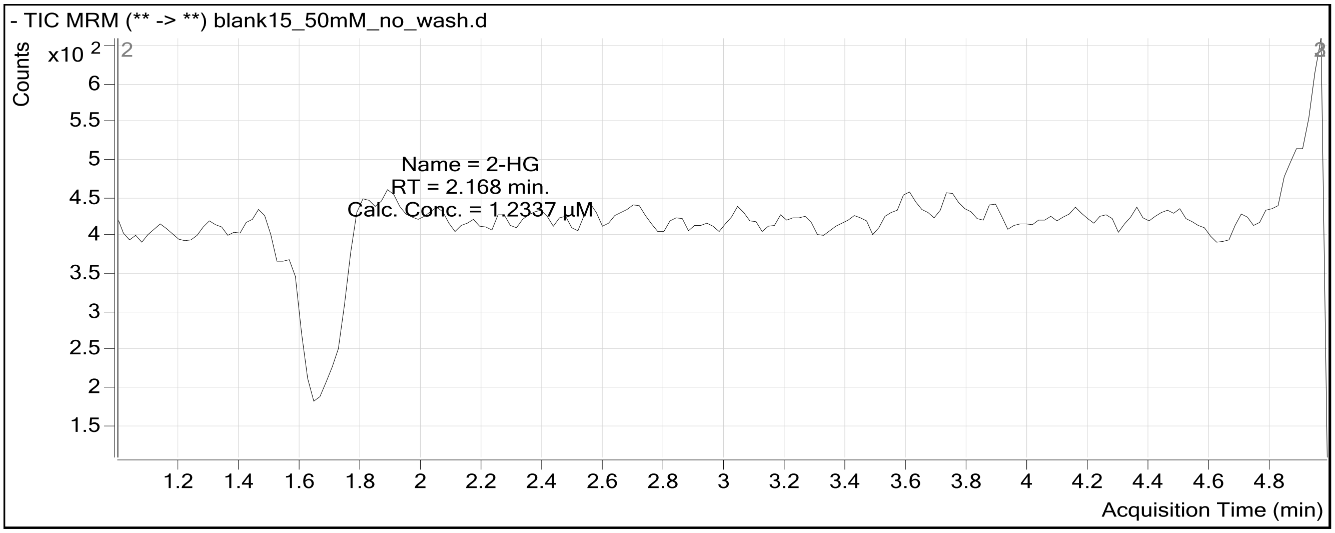

Quantitation Results

Compound	ISTD	RT	Response	ISTD Resp	RR	Conc.	Accuracy
2-HG		2.168	19			1.23	

Compound Graphics

Target Compound *2-HG*

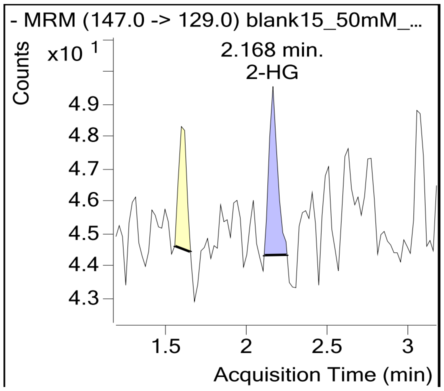

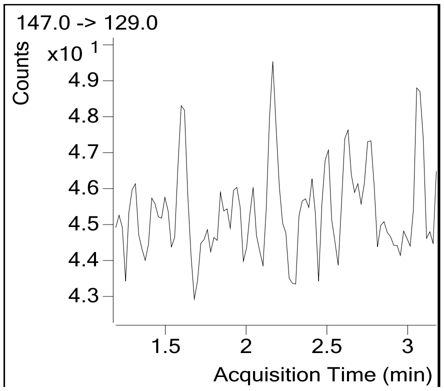

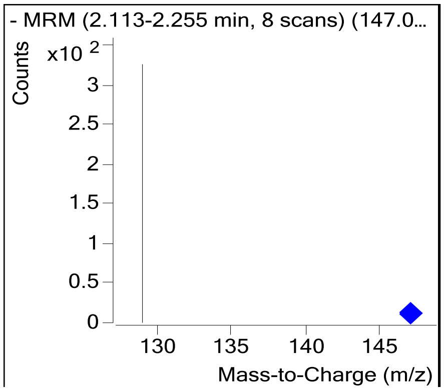

Quantitative Analysis Sample Report

**Batch Results**	D:\MassHunter\Data\Users\062023\Walstrom\LCMS6_13\QuantResults\2-HG_6_13analysis_batch.batch.bin		
**Analysis Time**	6/14/2023 11:06 AM	**Analyst Name**	admin
**Report Time**	6/14/2023 11:13 AM	**Reporter Name**	admin
**Last Calib Update**	6/14/2023 11:06 AM	**Batch State**	Processed

Analysis Info

**Acq Time**	2023-06-13 19:24	**Data File**	blank17_50mM_no_wash.d
**Position**	P1-B3	**Sample Name**	blank_50mM_17
**Dilution**	1	**Sample Info**	
**Inj. Volume**	Per method	**Acq Method File**	NCF_LCMS_neg_IDH.m
**Sample Type**	Blank	**Comment**	

Sample Chromatogram

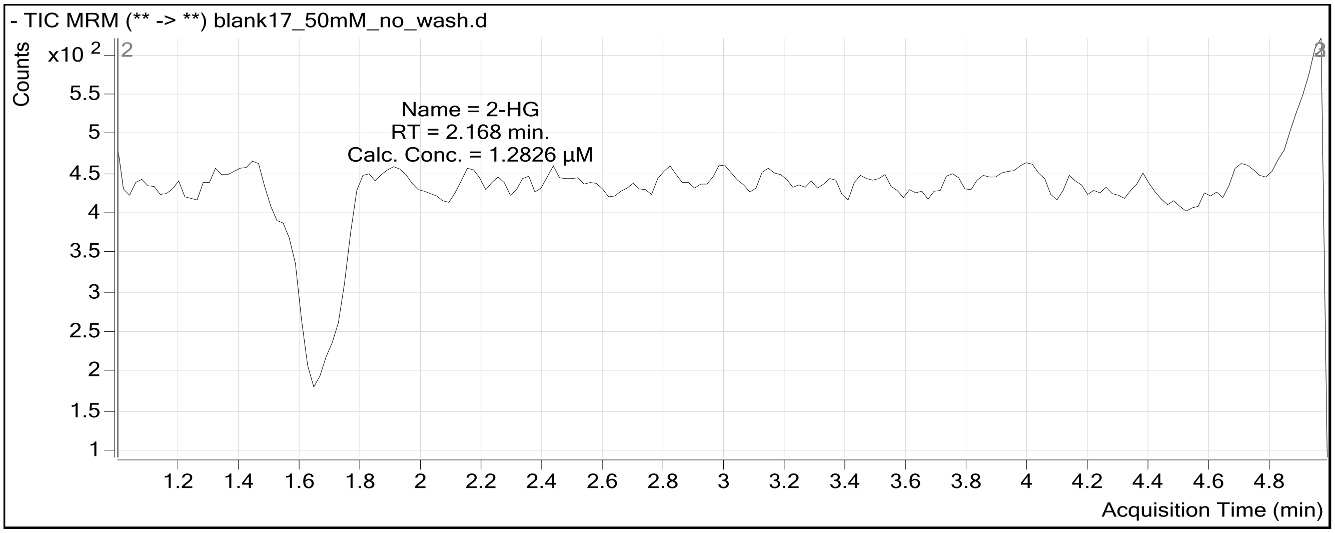

Quantitation Results

Compound	ISTD	RT	Response	ISTD Resp	RR	Conc.	Accuracy
2-HG		2.168	141			1.28	

Compound Graphics

Target Compound *2-HG*

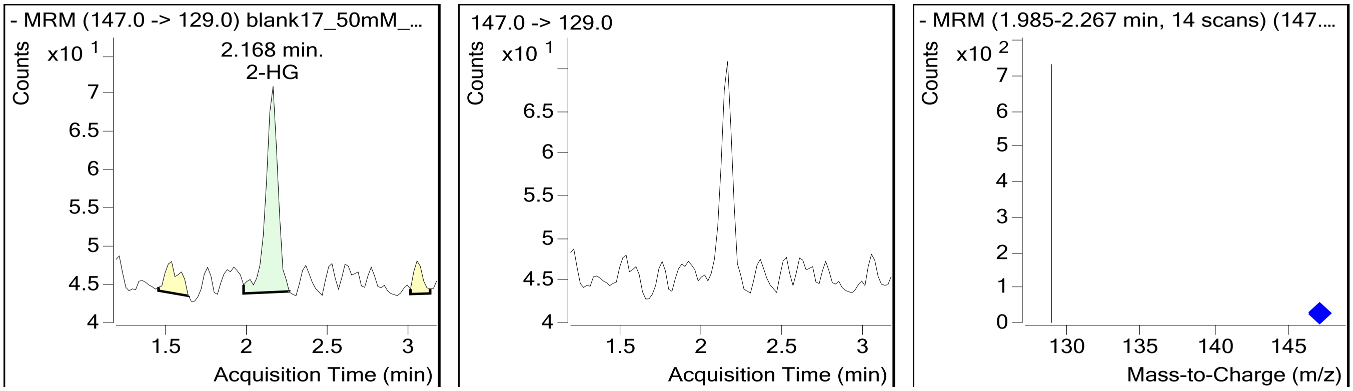

Quantitative Analysis Sample Report

**Batch Results**	D:\MassHunter\Data\Users\062023\Walstrom\LCMS6_13\QuantResults\2-HG_6_13analysis_batch.batch.bin		
**Analysis Time**	6/14/2023 11:06 AM	**Analyst Name**	admin
**Report Time**	6/14/2023 11:13 AM	**Reporter Name**	admin
**Last Calib Update**	6/14/2023 11:06 AM	**Batch State**	Processed

Analysis Info

**Acq Time**	2023-06-13 20:07	**Data File**	blank18_50mM_no_wash.d
**Position**	P1-B7	**Sample Name**	blank_50mM_18
**Dilution**	1	**Sample Info**	
**Inj. Volume**	Per method	**Acq Method File**	NCF_LCMS_neg_IDH.m
**Sample Type**	Blank	**Comment**	

Sample Chromatogram

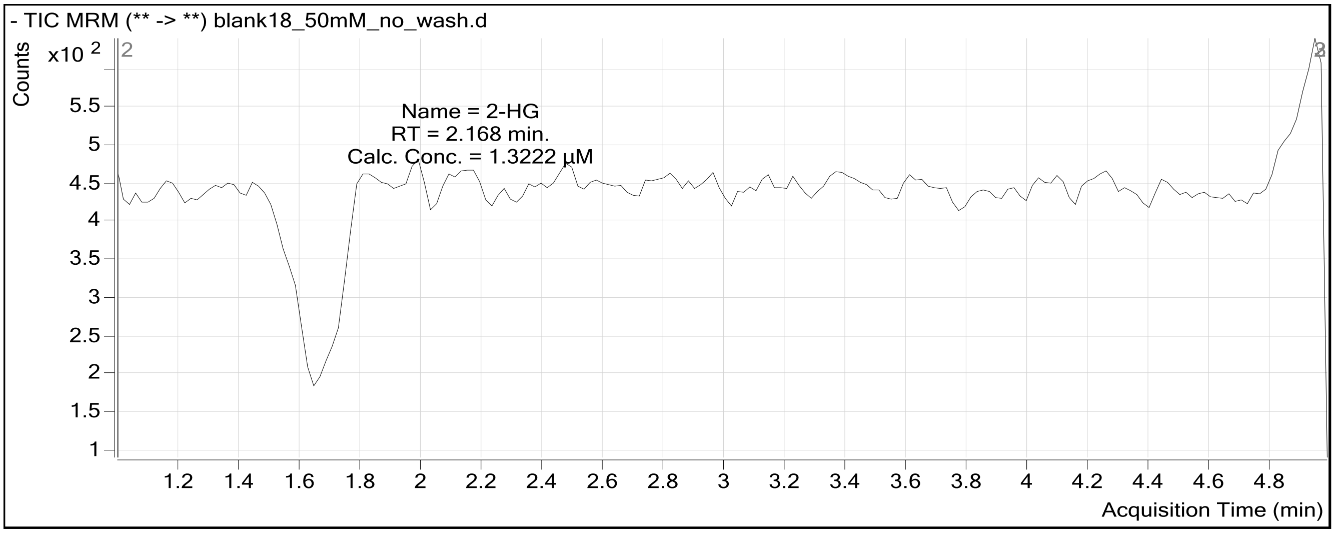

Quantitation Results

Compound	ISTD	RT	Response	ISTD Resp	RR	Conc.	Accuracy
2-HG		2.168	241			1.32	

Compound Graphics

Target Compound *2-HG*

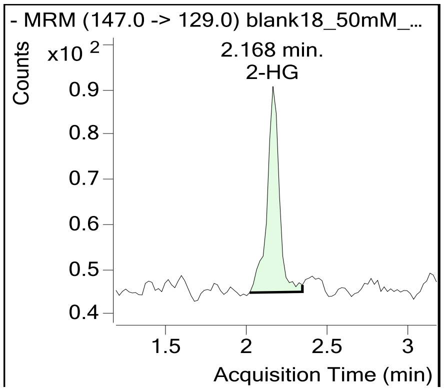

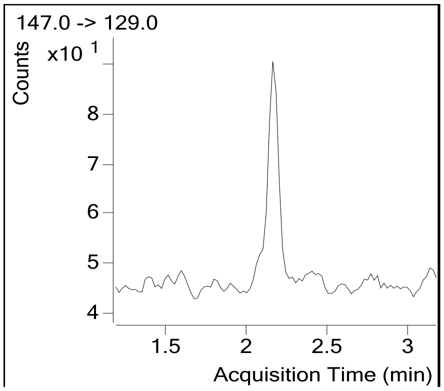

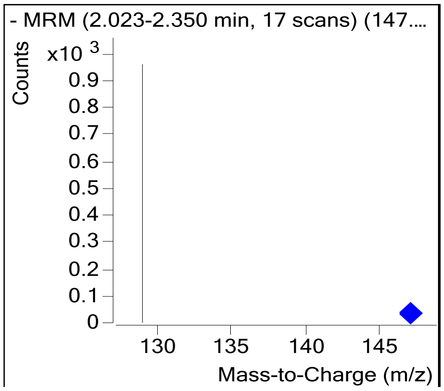

Quantitative Analysis Sample Report

**Batch Results**	D:\MassHunter\Data\Users\062023\Walstrom\LCMS6_13\QuantResults\2-HG_6_13analysis_batch.batch.bin		
**Analysis Time**	6/14/2023 11:06 AM	**Analyst Name**	admin
**Report Time**	6/14/2023 11:13 AM	**Reporter Name**	admin
**Last Calib Update**	6/14/2023 11:06 AM	**Batch State**	Processed

Analysis Info

**Acq Time**	2023-06-13 14:32	**Data File**	WT_1a.d
**Position**	P1-A7	**Sample Name**	WT_1a
**Dilution**	1	**Sample Info**	
**Inj. Volume**	Per method	**Acq Method File**	NCF_LCMS_neg_IDH.m
**Sample Type**	Sample	**Comment**	

Sample Chromatogram

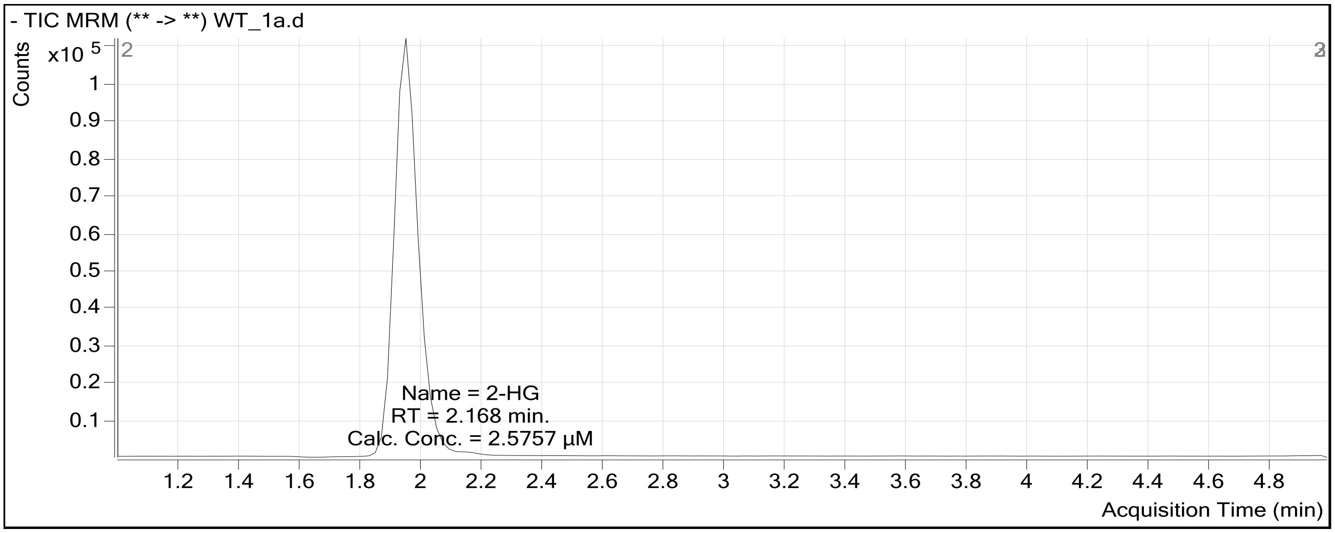

Quantitation Results

Compound	ISTD	RT	Response	ISTD Resp	RR	Conc.	Accuracy
2-HG		2.168	3386			2.58	

Compound Graphics

Target Compound *2-HG*

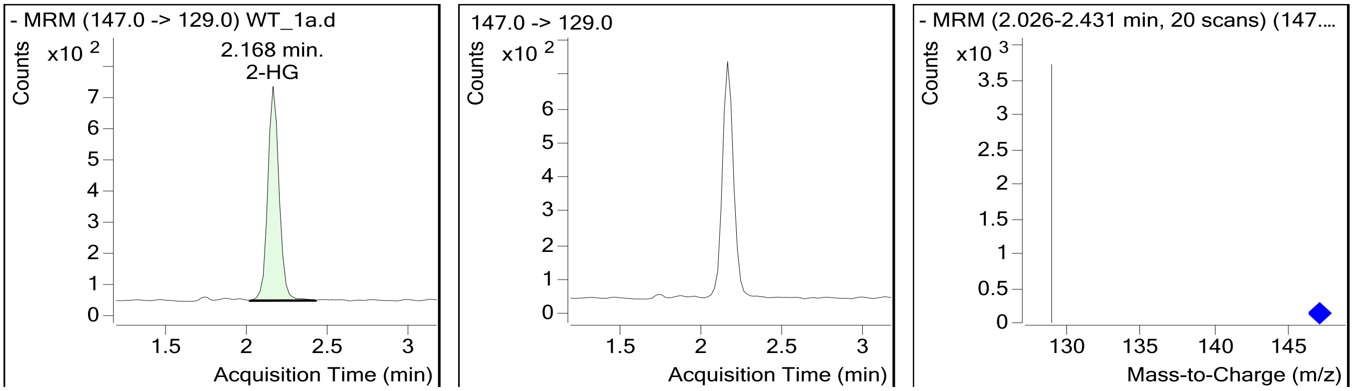

Quantitative Analysis Sample Report

**Batch Results**	D:\MassHunter\Data\Users\062023\Walstrom\LCMS6_13\QuantResults\2-HG_6_13analysis_batch.batch.bin		
**Analysis Time**	6/14/2023 11:06 AM	**Analyst Name**	admin
**Report Time**	6/14/2023 11:13 AM	**Reporter Name**	admin
**Last Calib Update**	6/14/2023 11:06 AM	**Batch State**	Processed

Analysis Info

**Acq Time**	2023-06-13 15:05	**Data File**	G98_1a.d
**Position**	P1-A8	**Sample Name**	G98_1a
**Dilution**	1	**Sample Info**	
**Inj. Volume**	Per method	**Acq Method File**	NCF_LCMS_neg_IDH.m
**Sample Type**	Sample	**Comment**	

Sample Chromatogram

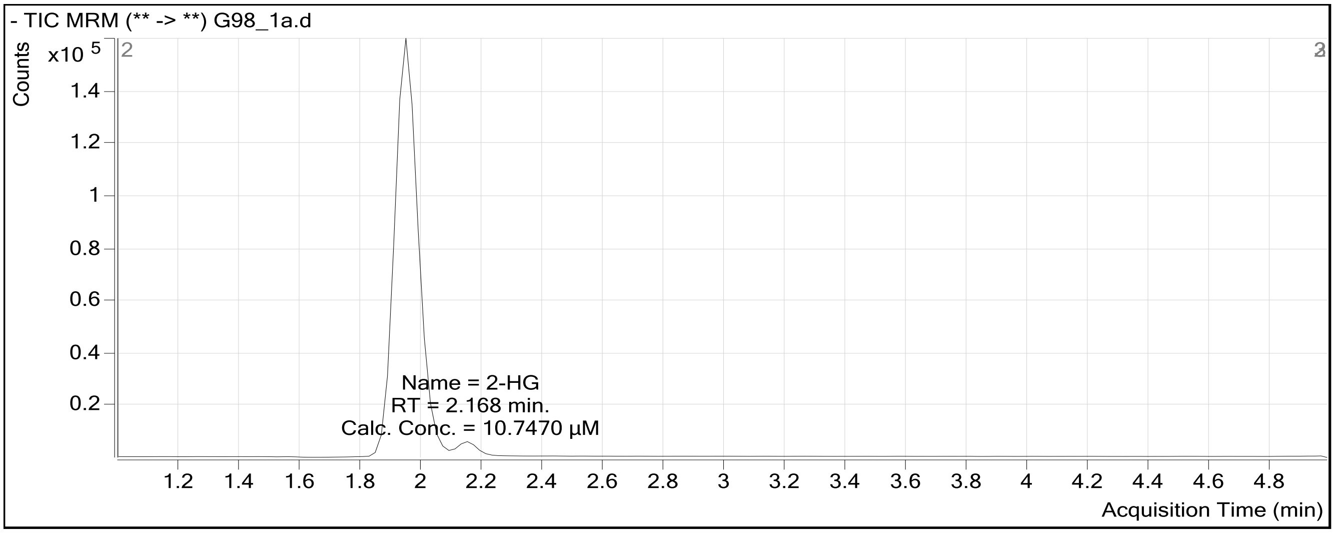

Quantitation Results

Compound	ISTD	RT	Response	ISTD Resp	RR	Conc.	Accuracy
2-HG		2.168	23887			10.75	

Compound Graphics

Target Compound *2-HG*

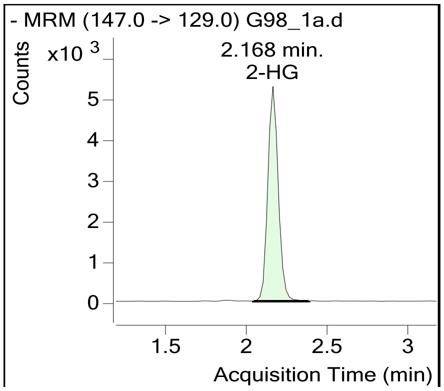

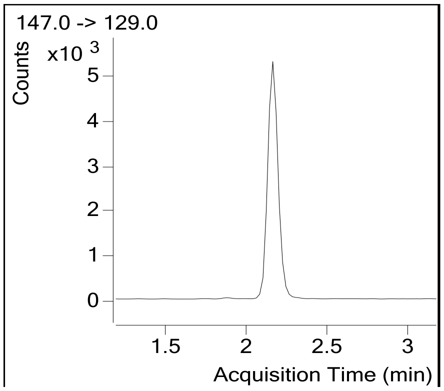

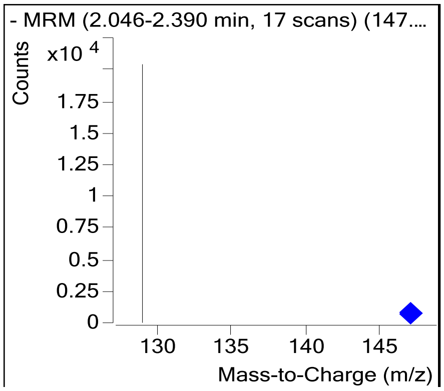

Quantitative Analysis Sample Report

**Batch Results**	D:\MassHunter\Data\Users\062023\Walstrom\LCMS6_13\QuantResults\2-HG_6_13analysis_batch.batch.bin		
**Analysis Time**	6/14/2023 11:06 AM	**Analyst Name**	admin
**Report Time**	6/14/2023 11:13 AM	**Reporter Name**	admin
**Last Calib Update**	6/14/2023 11:06 AM	**Batch State**	Processed

Analysis Info

**Acq Time**	2023-06-13 15:26	**Data File**	R133_1.d
**Position**	P1-A9	**Sample Name**	R133_1
**Dilution**	1	**Sample Info**	
**Inj. Volume**	Per method	**Acq Method File**	NCF_LCMS_neg_IDH.m
**Sample Type**	Sample	**Comment**	

Sample Chromatogram

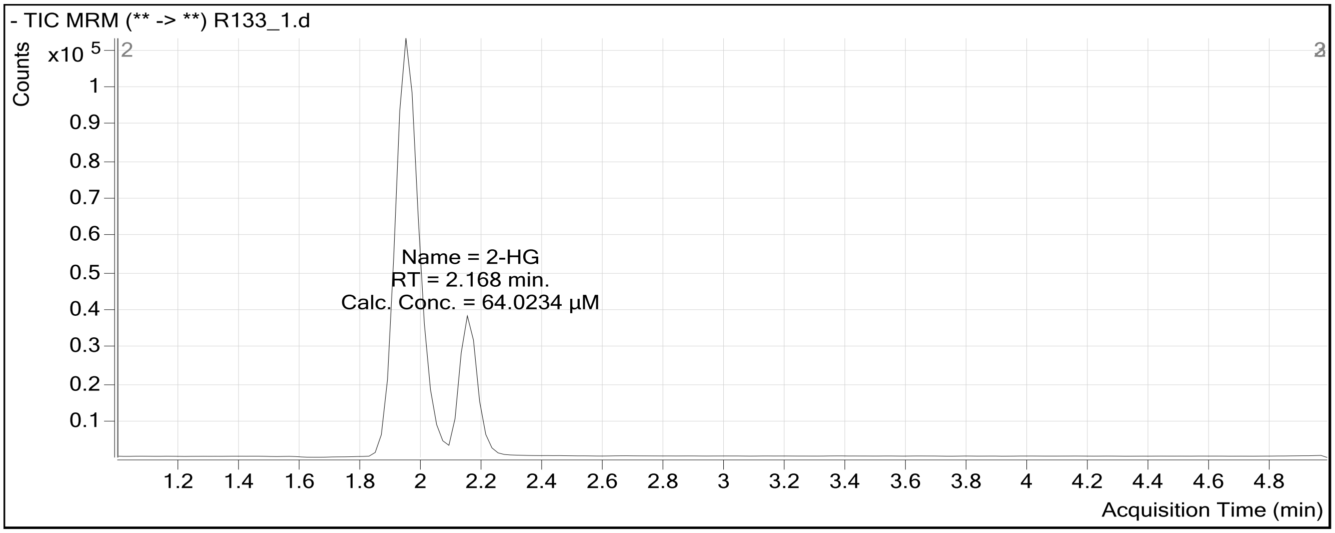

Quantitation Results

Compound	ISTD	RT	Response	ISTD Resp	RR	Conc.	Accuracy
2-HG		2.168	157553			64.02	

Compound Graphics

Target Compound *2-HG*

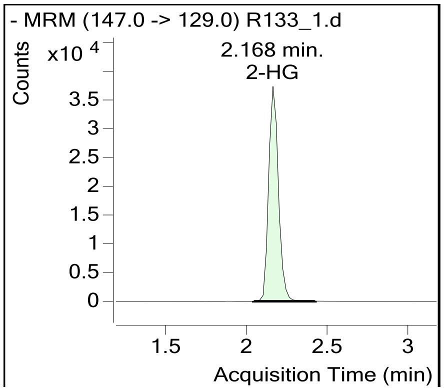

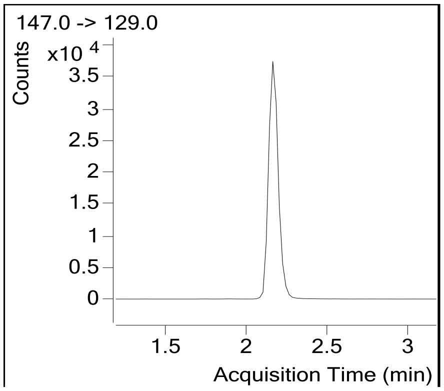

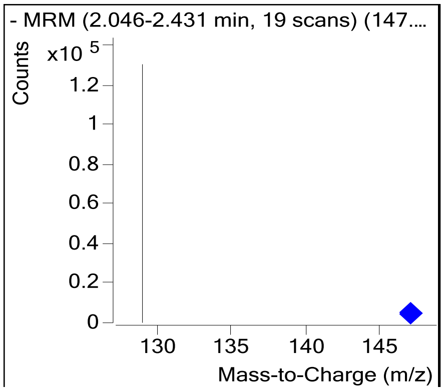

Quantitative Analysis Sample Report

**Batch Results**	D:\MassHunter\Data\Users\062023\Walstrom\LCMS6_13\QuantResults\2-HG_6_13analysis_batch.batch.bin		
**Analysis Time**	6/14/2023 11:06 AM	**Analyst Name**	admin
**Report Time**	6/14/2023 11:13 AM	**Reporter Name**	admin
**Last Calib Update**	6/14/2023 11:06 AM	**Batch State**	Processed

Analysis Info

**Acq Time**	2023-06-13 15:59	**Data File**	G98_1a_slow.d
**Position**	P1-A8	**Sample Name**	G98_1a
**Dilution**	1	**Sample Info**	
**Inj. Volume**	Per method	**Acq Method File**	NCF_LCMS_neg_IDH.m
**Sample Type**	Sample	**Comment**	

Sample Chromatogram

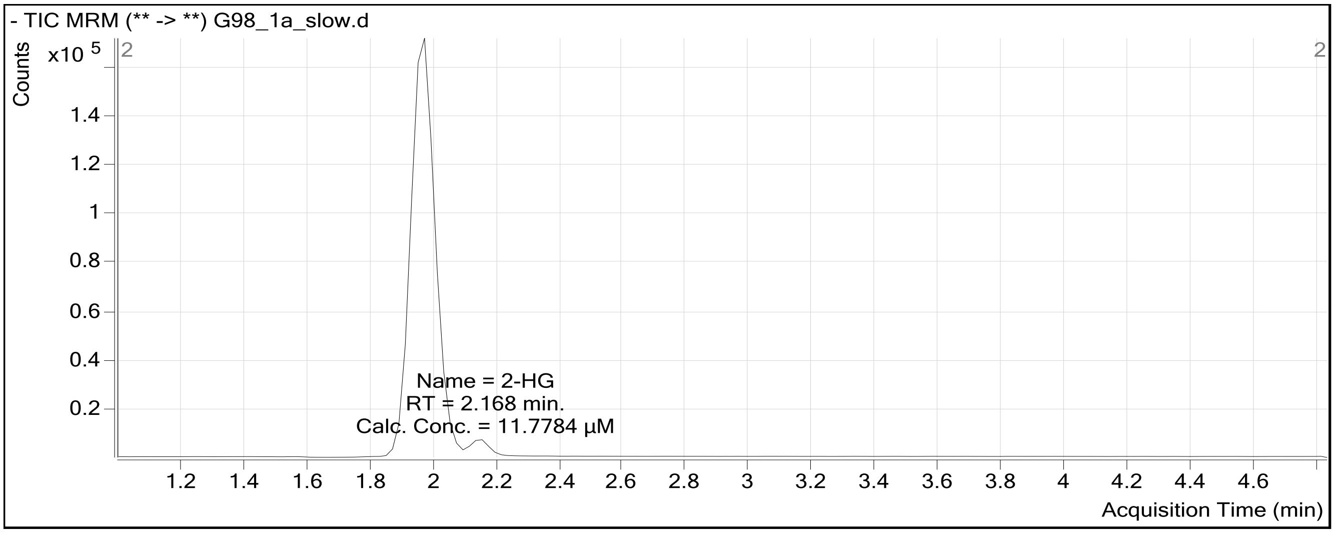

Quantitation Results

Compound	ISTD	RT	Response	ISTD Resp	RR	Conc.	Accuracy
2-HG		2.168	26474			11.78	

Compound Graphics

Target Compound *2-HG*

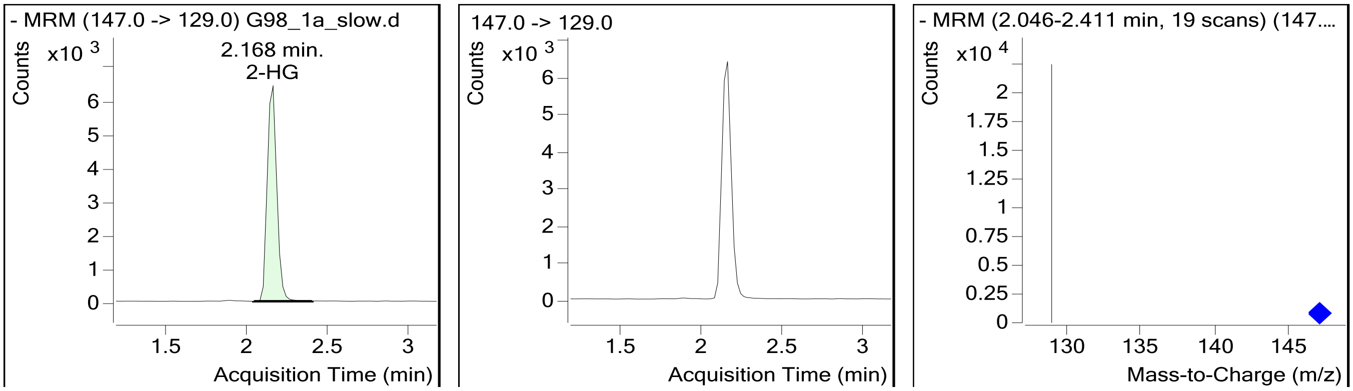

Quantitative Analysis Sample Report

**Batch Results**	D:\MassHunter\Data\Users\062023\Walstrom\LCMS6_13\QuantResults\2-HG_6_13analysis_batch.batch.bin		
**Analysis Time**	6/14/2023 11:06 AM	**Analyst Name**	admin
**Report Time**	6/14/2023 11:13 AM	**Reporter Name**	admin
**Last Calib Update**	6/14/2023 11:06 AM	**Batch State**	Processed

Analysis Info

**Acq Time**	2023-06-13 17:57	**Data File**	WT_1a_2nd_run.d
**Position**	P1-A1	**Sample Name**	WT_1a_2
**Dilution**	1	**Sample Info**	
**Inj. Volume**	Per method	**Acq Method File**	NCF_LCMS_neg_IDH.m
**Sample Type**	Sample	**Comment**	

Sample Chromatogram

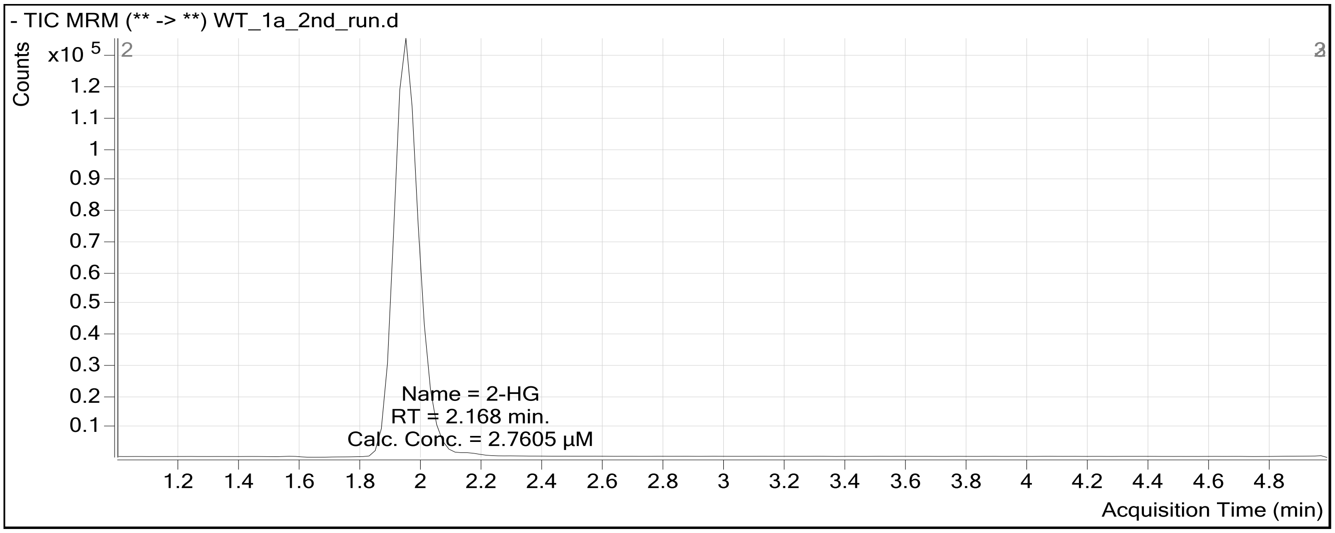

Quantitation Results

Compound	ISTD	RT	Response	ISTD Resp	RR	Conc.	Accuracy
2-HG		2.168	3849			2.76	

Compound Graphics

Target Compound *2-HG*

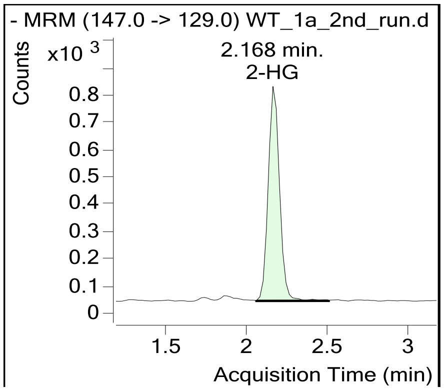

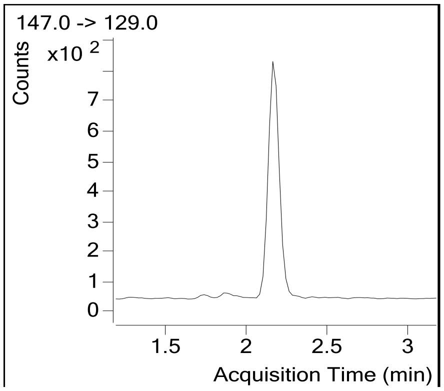

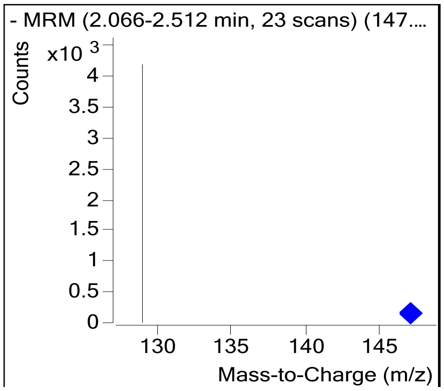

Quantitative Analysis Sample Report

**Batch Results**	D:\MassHunter\Data\Users\062023\Walstrom\LCMS6_13\QuantResults\2-HG_6_13analysis_batch.batch.bin		
**Analysis Time**	6/14/2023 11:06 AM	**Analyst Name**	admin
**Report Time**	6/14/2023 11:13 AM	**Reporter Name**	admin
**Last Calib Update**	6/14/2023 11:06 AM	**Batch State**	Processed

Analysis Info

**Acq Time**	2023-06-13 18:08	**Data File**	WT_1b.d
**Position**	P1-A4	**Sample Name**	WT_1b
**Dilution**	1	**Sample Info**	
**Inj. Volume**	Per method	**Acq Method File**	NCF_LCMS_neg_IDH.m
**Sample Type**	Sample	**Comment**	

Sample Chromatogram

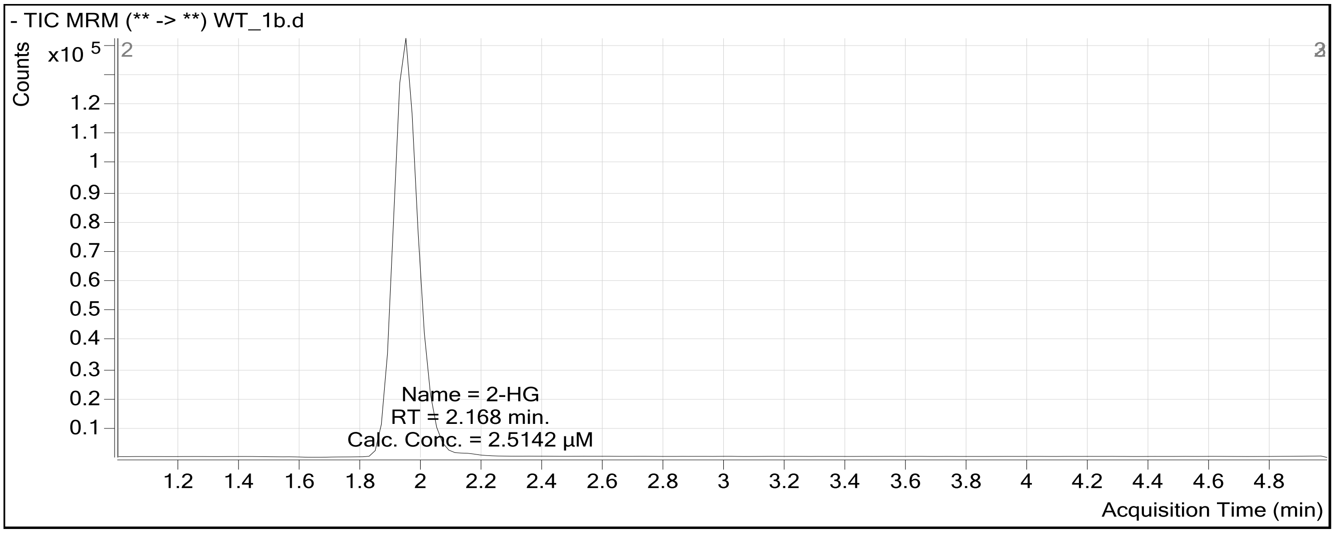

Quantitation Results

Compound	ISTD	RT	Response	ISTD Resp	RR	Conc.	Accuracy
2-HG		2.168	3232			2.51	

Compound Graphics

Target Compound *2-HG*

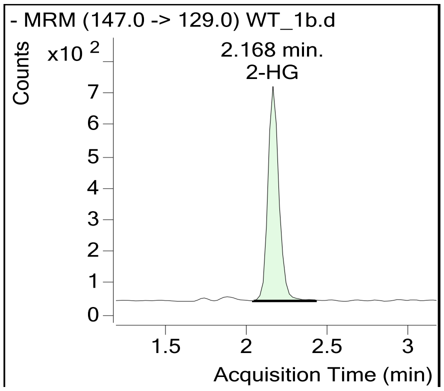

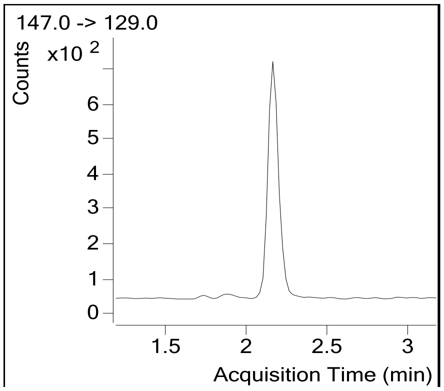

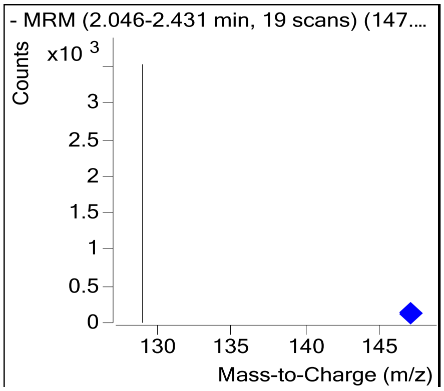

Quantitative Analysis Sample Report

**Batch Results**	D:\MassHunter\Data\Users\062023\Walstrom\LCMS6_13\QuantResults\2-HG_6_13analysis_batch.batch.bin		
**Analysis Time**	6/14/2023 11:06 AM	**Analyst Name**	admin
**Report Time**	6/14/2023 11:13 AM	**Reporter Name**	admin
**Last Calib Update**	6/14/2023 11:06 AM	**Batch State**	Processed

Analysis Info

**Acq Time**	2023-06-13 18:19	**Data File**	WT_2.d
**Position**	P1-A5	**Sample Name**	WT_2
**Dilution**	1	**Sample Info**	
**Inj. Volume**	Per method	**Acq Method File**	NCF_LCMS_neg_IDH.m
**Sample Type**	Sample	**Comment**	

Sample Chromatogram

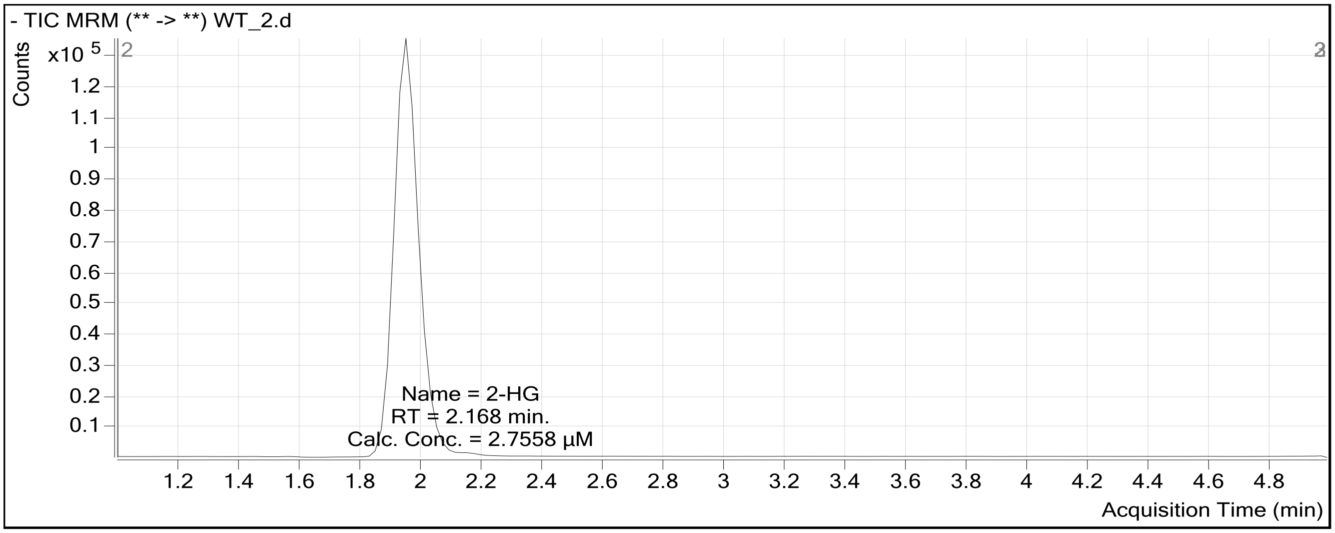

Quantitation Results

Compound	ISTD	RT	Response	ISTD Resp	RR	Conc.	Accuracy
2-HG		2.168	3837			2.76	

Compound Graphics

Target Compound *2-HG*

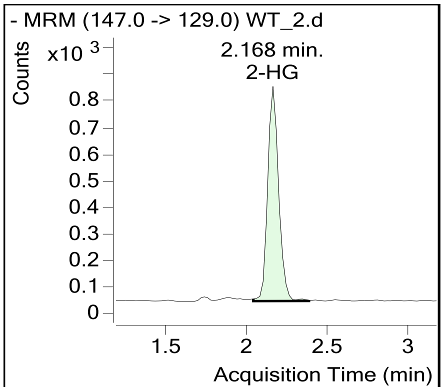

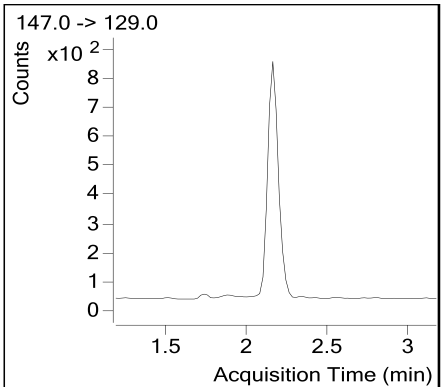

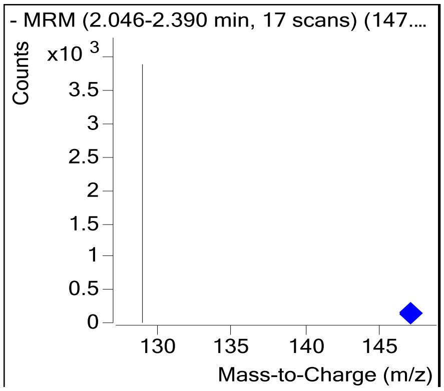

Quantitative Analysis Sample Report

**Batch Results**	D:\MassHunter\Data\Users\062023\Walstrom\LCMS6_13\QuantResults\2-HG_6_13analysis_batch.batch.bin		
**Analysis Time**	6/14/2023 11:06 AM	**Analyst Name**	admin
**Report Time**	6/14/2023 11:13 AM	**Reporter Name**	admin
**Last Calib Update**	6/14/2023 11:06 AM	**Batch State**	Processed

Analysis Info

**Acq Time**	2023-06-13 18:51	**Data File**	G98_1b.d
**Position**	P1-A8	**Sample Name**	G98_1b
**Dilution**	1	**Sample Info**	
**Inj. Volume**	Per method	**Acq Method File**	NCF_LCMS_neg_IDH.m
**Sample Type**	Sample	**Comment**	

Sample Chromatogram

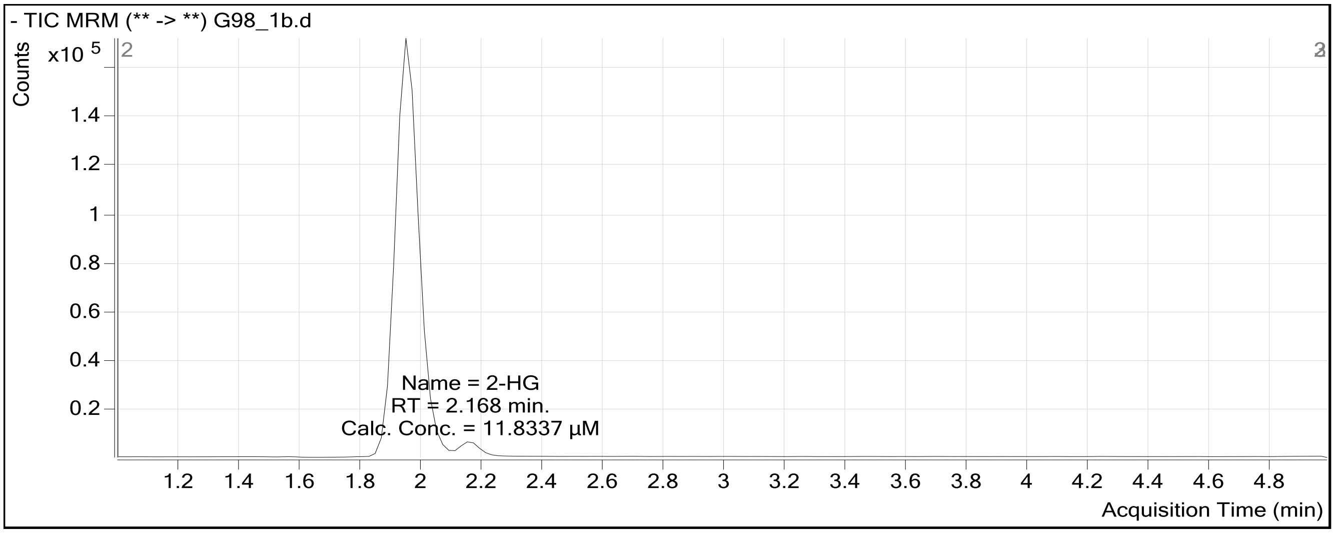

Quantitation Results

Compound	ISTD	RT	Response	ISTD Resp	RR	Conc.	Accuracy
2-HG		2.168	26613			11.83	

Compound Graphics

Target Compound *2-HG*

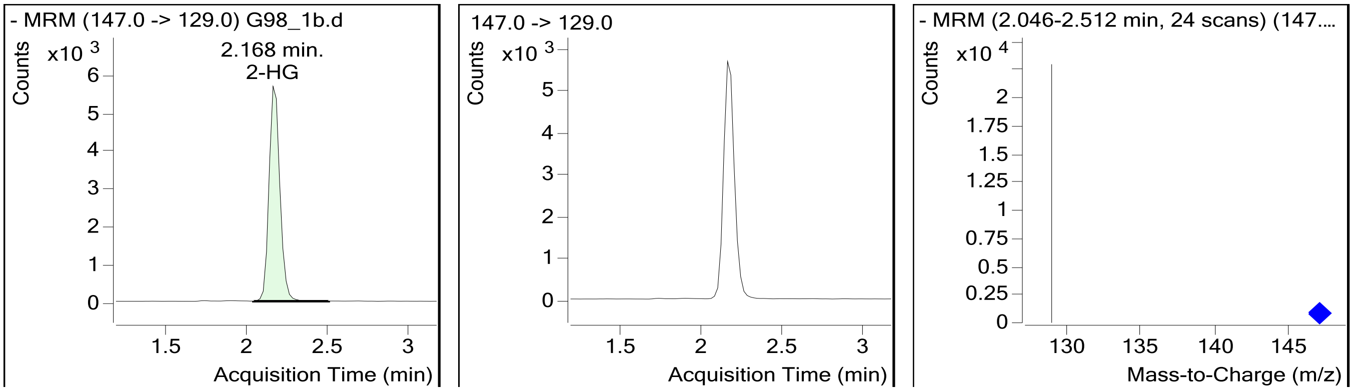

Quantitative Analysis Sample Report

**Batch Results**	D:\MassHunter\Data\Users\062023\Walstrom\LCMS6_13\QuantResults\2-HG_6_13analysis_batch.batch.bin		
**Analysis Time**	6/14/2023 11:06 AM	**Analyst Name**	admin
**Report Time**	6/14/2023 11:13 AM	**Reporter Name**	admin
**Last Calib Update**	6/14/2023 11:06 AM	**Batch State**	Processed

Analysis Info

**Acq Time**	2023-06-13 19:02	**Data File**	G98_2.d
**Position**	P1-B1	**Sample Name**	G98_2
**Dilution**	1	**Sample Info**	
**Inj. Volume**	Per method	**Acq Method File**	NCF_LCMS_neg_IDH.m
**Sample Type**	Sample	**Comment**	

Sample Chromatogram

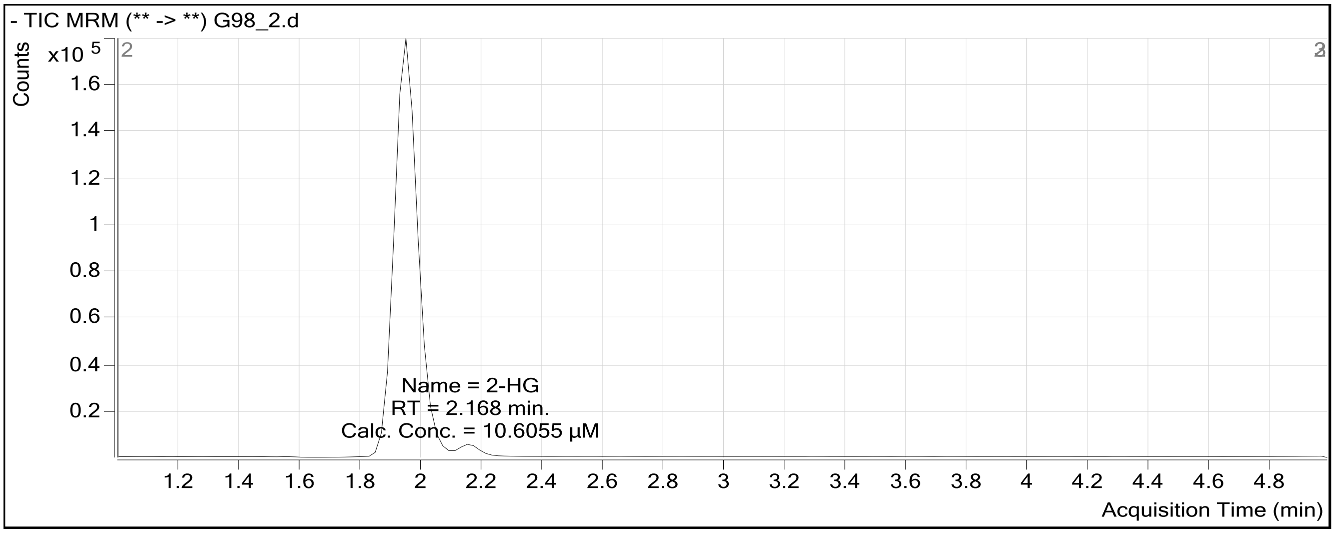

Quantitation Results

Compound	ISTD	RT	Response	ISTD Resp	RR	Conc.	Accuracy
2-HG		2.168	23532			10.61	

Compound Graphics

Target Compound *2-HG*

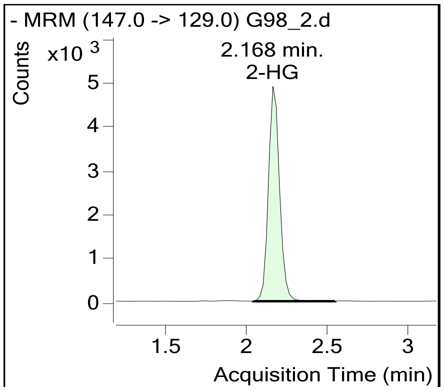

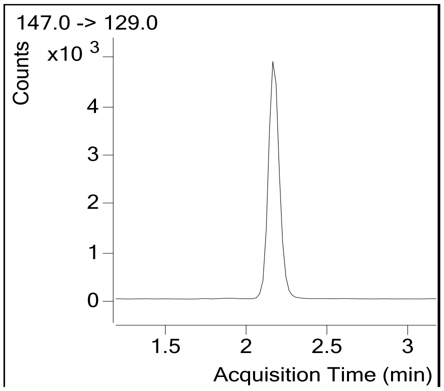

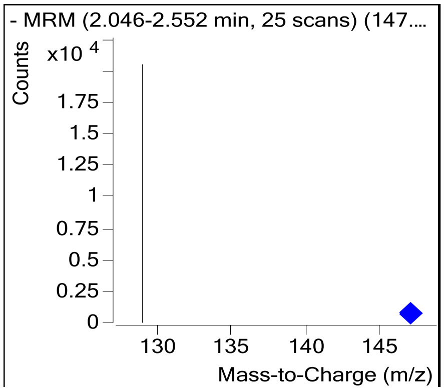

Quantitative Analysis Sample Report

**Batch Results**	D:\MassHunter\Data\Users\062023\Walstrom\LCMS6_13\QuantResults\2-HG_6_13analysis_batch.batch.bin		
**Analysis Time**	6/14/2023 11:06 AM	**Analyst Name**	admin
**Report Time**	6/14/2023 11:13 AM	**Reporter Name**	admin
**Last Calib Update**	6/14/2023 11:06 AM	**Batch State**	Processed

Analysis Info

**Acq Time**	2023-06-13 19:35	**Data File**	R133_1_2nd_run.d
**Position**	P1-B4	**Sample Name**	R133_1
**Dilution**	1	**Sample Info**	
**Inj. Volume**	Per method	**Acq Method File**	NCF_LCMS_neg_IDH.m
**Sample Type**	Sample	**Comment**	

Sample Chromatogram

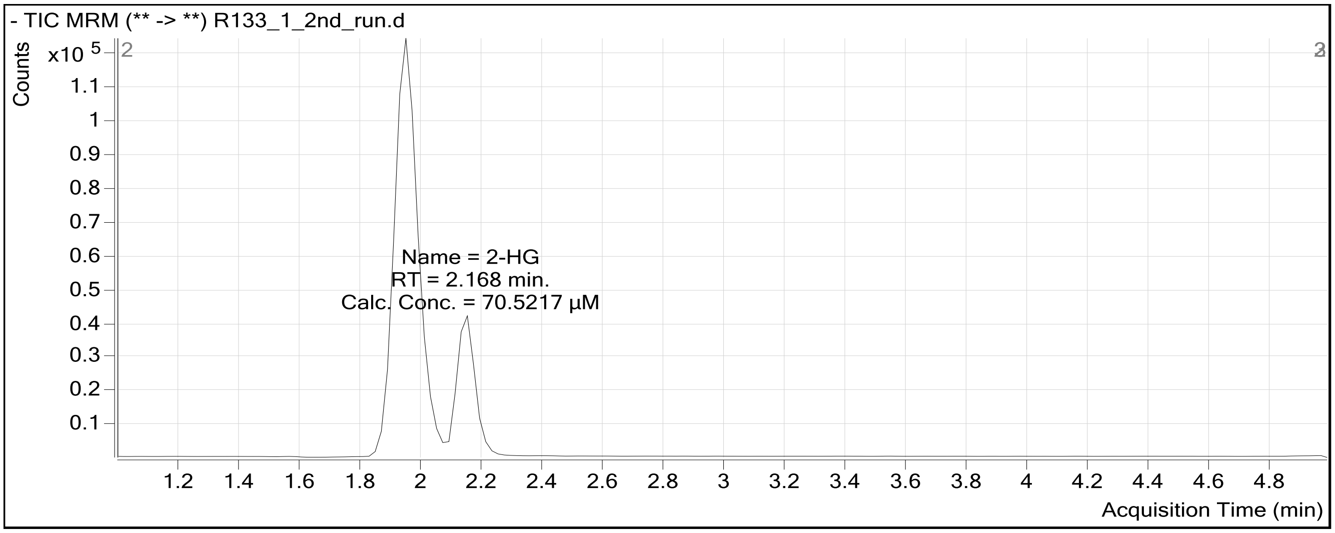

Quantitation Results

Compound	ISTD	RT	Response	ISTD Resp	RR	Conc.	Accuracy
2-HG		2.168	173856			70.52	

Compound Graphics

Target Compound *2-HG*

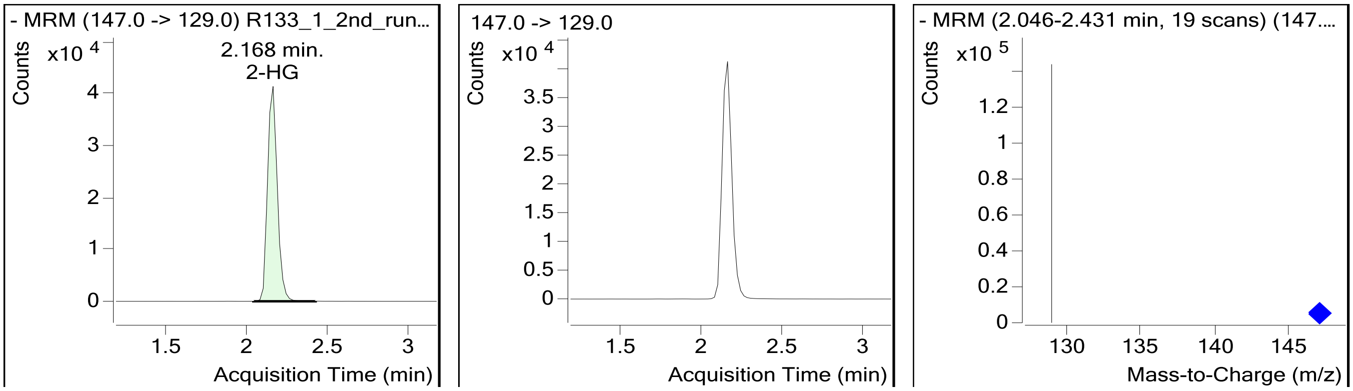

Quantitative Analysis Sample Report

**Batch Results**	D:\MassHunter\Data\Users\062023\Walstrom\LCMS6_13\QuantResults\2-HG_6_13analysis_batch.batch.bin		
**Analysis Time**	6/14/2023 11:06 AM	**Analyst Name**	admin
**Report Time**	6/14/2023 11:13 AM	**Reporter Name**	admin
**Last Calib Update**	6/14/2023 11:06 AM	**Batch State**	Processed

Analysis Info

**Acq Time**	2023-06-13 19:46	**Data File**	R133_2.d
**Position**	P1-B5	**Sample Name**	R133_2
**Dilution**	1	**Sample Info**	
**Inj. Volume**	Per method	**Acq Method File**	NCF_LCMS_neg_IDH.m
**Sample Type**	Sample	**Comment**	

Sample Chromatogram

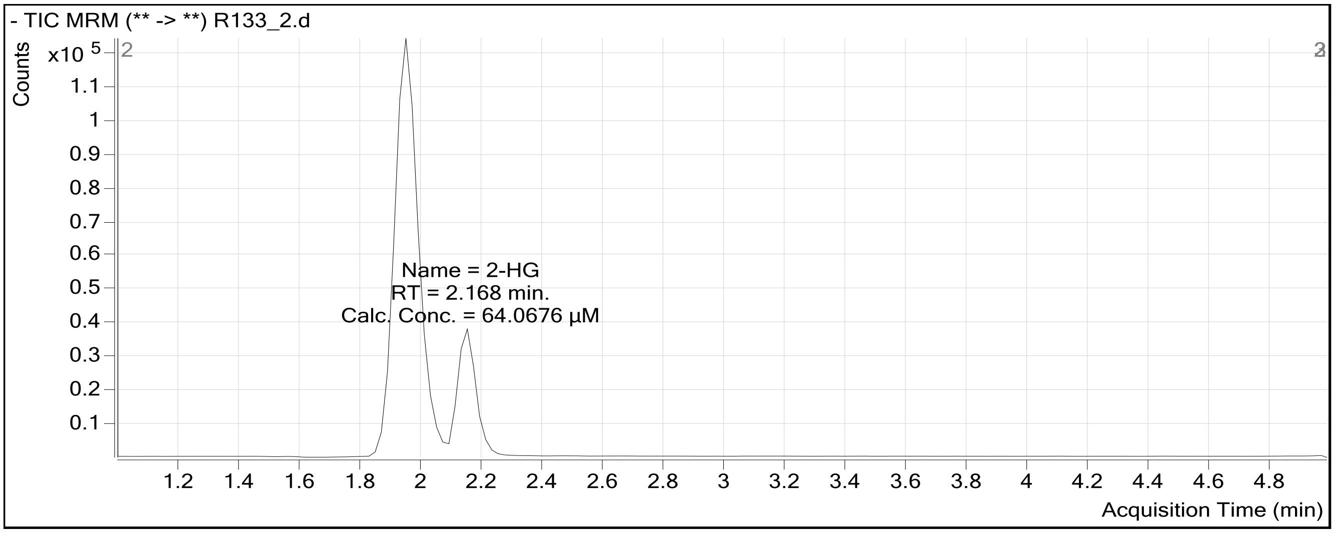

Quantitation Results

Compound	ISTD	RT	Response	ISTD Resp	RR	Conc.	Accuracy
2-HG		2.168	157663			64.07	

Compound Graphics

Target Compound *2-HG*

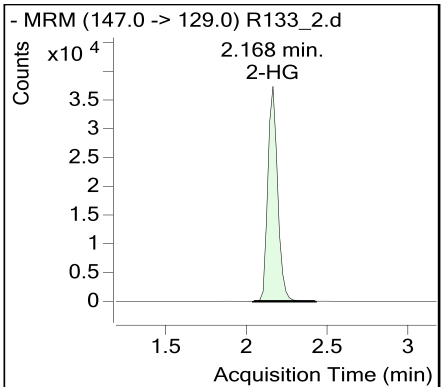

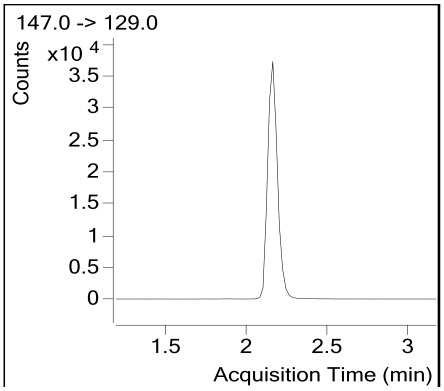

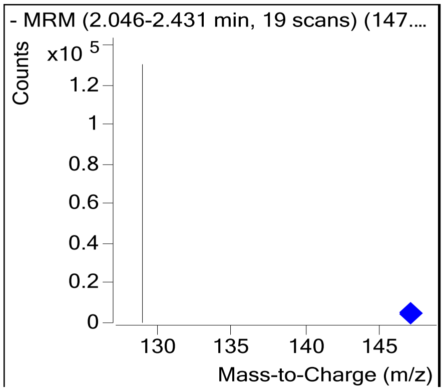

Quantitative Analysis Sample Report

**Batch Results**	D:\MassHunter\Data\Users\062023\Walstrom\LCMS6_13\QuantResults\2-HG_6_13analysis_batch.batch.bin		
**Analysis Time**	6/14/2023 11:06 AM	**Analyst Name**	admin
**Report Time**	6/14/2023 11:13 AM	**Reporter Name**	admin
**Last Calib Update**	6/14/2023 11:06 AM	**Batch State**	Processed

Analysis Info

**Acq Time**	2023-06-13 19:57	**Data File**	R133_3.d
**Position**	P1-B6	**Sample Name**	R133_3
**Dilution**	1	**Sample Info**	
**Inj. Volume**	Per method	**Acq Method File**	NCF_LCMS_neg_IDH.m
**Sample Type**	Sample	**Comment**	

Sample Chromatogram

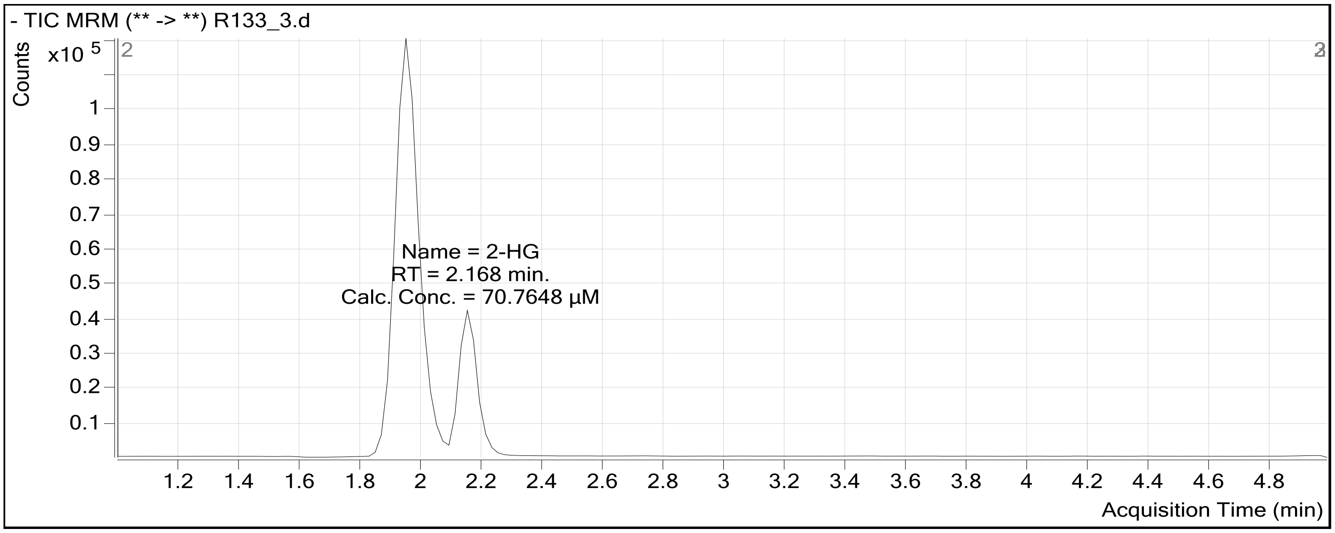

Quantitation Results

Compound	ISTD	RT	Response	ISTD Resp	RR	Conc.	Accuracy
2-HG		2.168	174466			70.76	

Compound Graphics

Target Compound *2-HG*

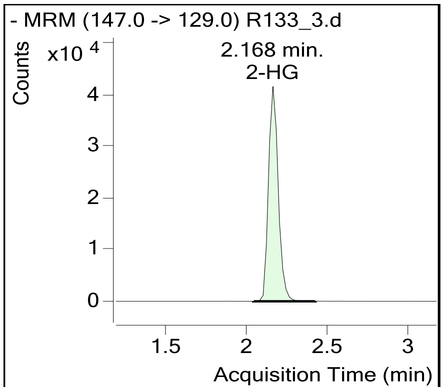

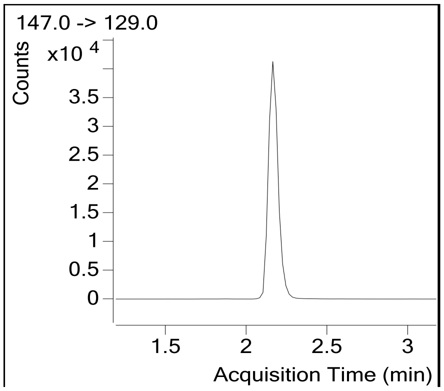

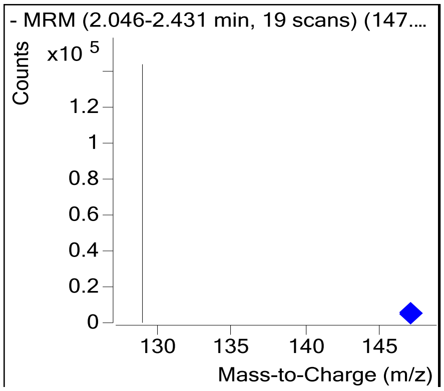

Quantitative Analysis Sample Report

**Batch Results**	D:\MassHunter\Data\Users\062023\Walstrom\LCMS6_13\QuantResults\2-HG_6_13analysis_batch.batch.bin		
**Analysis Time**	6/14/2023 11:06 AM	**Analyst Name**	admin
**Report Time**	6/14/2023 11:13 AM	**Reporter Name**	admin
**Last Calib Update**	6/14/2023 11:06 AM	**Batch State**	Processed

Analysis Info

**Acq Time**	2023-06-13 20:18	**Data File**	rxnmix_div_2.d
**Position**	P1-B8	**Sample Name**	rxn_mix_div_2
**Dilution**	1	**Sample Info**	
**Inj. Volume**	Per method	**Acq Method File**	NCF_LCMS_neg_IDH.m
**Sample Type**	Sample	**Comment**	

Sample Chromatogram

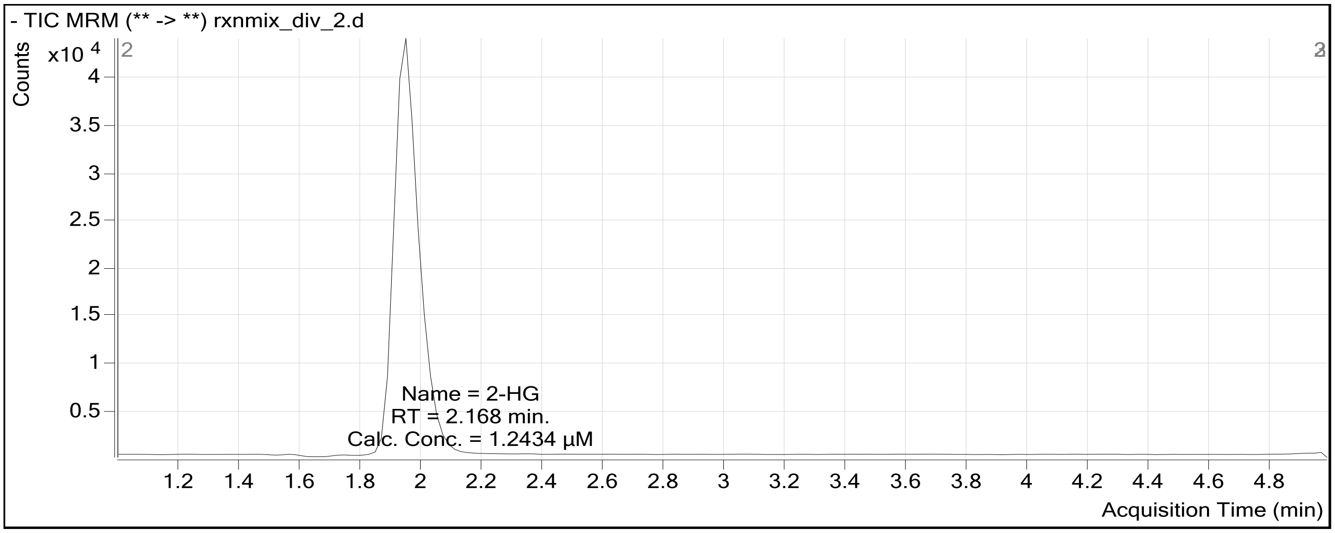

Quantitation Results

Compound	ISTD	RT	Response	ISTD Resp	RR	Conc.	Accuracy
2-HG		2.168	43			1.24	

Compound Graphics

Target Compound *2-HG*

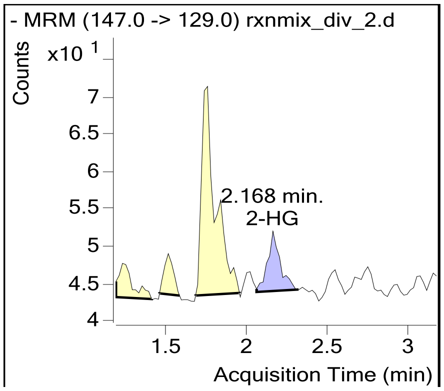

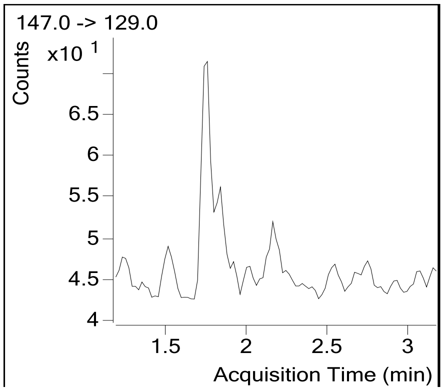

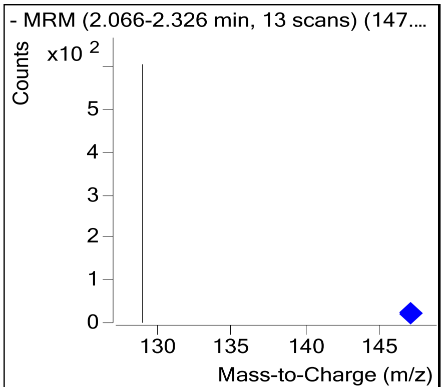

Supplement: Supplementary file 1 [file ijms-26-08238-s001.zip › FigureS5.pdf]
