# Supplementary material for: C. elegans Cytoplasmic Isocitrate Dehydrogenase Neomorphic G98N and R133H Mutants Produce the Oncometabolite 2-Hydroxyglutarate
Source: Int J Mol Sci. 2025 Aug 25;26(17):8238. doi: 10.3390/ijms26178238 (PMC12427979; doi:10.3390/ijms26178238)
Supplement: Supplementary file 1 [file ijms-26-08238-s001.zip › TableS1.pdf]

Table S1. Steady-state Enzyme Kinetic Fits and Statistics for IDH-1 at 25 °C

Enzyme	Varied substrate	Coefficient	Estimate	Std. Error*	t value	Pr(>|t|)
Wild-type IDH-1	ICT	k_{cat}, min^{-1}	2509	71	35.5	1.85E-25
		K_M, μM	6.9	0.8	8.3	3.57E-09
Wild-type IDH-1	$NADP^+$	k_{cat}, min^{-1}	1259	47	26.7	9.46E-18
		K_M, μM	3.3	0.5	6.2	3.45E-06
Wild-type IDH-1	Mn^{2+}	k_{cat}, min^{-1}	1591	59	27	8.43E-15
		K_M, μM	9	1.7	5.5	5.09E-05
		Co, μM	0.64	0.59	1.09	0.29
G98N	ICT	k_{cat}, min^{-1}	190	4.2	45	1.32E-16
		K_M, μM	13	1.0	12.9	3.51E-09
G98N	$NADP^+$	k_{cat}, min^{-1}	104	2.5	42	9.59E-18
		K_M, μM	2.8	0.3	9.3	7.74E-08
R133H	ICT	k_{cat}, min^{-1}	11.7	0.5	22.8	5.90E-10
		K_M, μM	2160	252	8.6	6.51E-06
R133H	αKG	k_{cat}, min^{-1}	18.1	0.6	29.5	2.28E-15
		K_M, μM	13.9	1.8	7.7	8.92E-07
R133H	NADPH	k_{cat}, min^{-1}	16.7	0.5	33.2	1.04E-14
		K_M, μM	1.7	0.19	8.8	4.56E-07

*The standard errors shown here are from the kinetic data fits only. The standard errors reported for k_{cat} in the manuscript include a propagation of errors from the error in the protein concentration measurements.
